# Supplementary material for: Reciprocal inhibition of NOTCH and SOX2 shapes tumor cell plasticity and therapeutic escape in triple-negative breast cancer
Source: EMBO Mol Med. 2024 Oct 30;16(12):9. doi: 10.1038/s44321-024-00161-8 (PMC11628624; doi:10.1038/s44321-024-00161-8)
Supplement: Supplementary file 1 — Table EV1 [file 44321_2024_161_MOESM1_ESM.docx]

Table EV 1: List of NOTCH target genes (77 genes).

| **Genes** |
| --- |
| *ACTBL2* |
| *ADAMTSL4* |
| *ANKRD1* |
| *ARRDC4* |
| *BMP6* |
| *CCND1* |
| *CDK5R1* |
| *CERS1* |
| *CPNE7* |
| *CXCL2* |
| *CYP1B1* |
| *DSG3* |
| *DUSP5* |
| *E2F2* |
| *ENC1* |
| *FAT2* |
| *FBLN7* |
| *FGFBP1* |
| *FLT1* |
| *GADD45A* |
| *GAS5* |
| *HES1* |
| *HES2* |
| *HES4* |
| *HES5* |
| *HEY1* |
| *HEY2* |
| *HEYL* |
| *ID3* |
| *IER3* |
| *IFFO2* |
| *IGFBP3* |
| *IL12A* |
| *IL20RA* |
| *INHBB* |
| *JAG1* |
| *JPH2* |
| *KCNG1* |
| *KCNK5* |
| *KRT5* |
| *KRT6A* |
| *KRT6B* |
| *KRT6C* |
| *KRT75* |
| *LAMA4* |
| *LURAP1L* |
| *LYPD5* |
| *MMP7* |
| *MT1X* |
| *MYC* |
| *MYCL* |
| *MYEOV* |
| *NCR3LG1* |
| *NRARP* |
| *OLFM4* |
| *P2RY6* |
| *PAPPA* |
| *PDZD2* |
| *PI3* |
| *PLK2* |
| *PMAIP1* |
| *PRR5L* |
| *RAB11FIP1* |
| *RHOV* |
| *SAT1* |
| *SCD5* |
| *SERPINA3* |
| *SERPINB9* |
| *SNAI1* |
| *STARD4* |
| *TFRC* |
| *TGFB2* |
| *XK* |
| *YPEL2* |
| *ZNF469* |
| *ZNF750* |
