# Supplementary material for: Reciprocal inhibition of NOTCH and SOX2 shapes tumor cell plasticity and therapeutic escape in triple-negative breast cancer
Source: EMBO Mol Med. 2024 Oct 30;16(12):9. doi: 10.1038/s44321-024-00161-8 (PMC11628624; doi:10.1038/s44321-024-00161-8)
Supplement: Supplementary file 2 — Table EV2 [file 44321_2024_161_MOESM2_ESM.docx]

Table EV2: List qPCR primers.

| qPCR Primers | | |
| --- | --- | --- |
| Gene | Forward | Reverse |
| *CCND1* | TCT ACA CCG ACA ACT CCA TCC G | TCT GGC ATT TTG GAG AGG AAG TG |
| *CD44* | CCA GAA GGA ACA GTG GTT TGG C | ACT GTC CTC TGG GCT TGG TGT T |
| *CDH1* | GCC TCC TGA AAA GAG AGT GGA AG | TGG CAG TGT CTC TCC AAA TCC G |
| *CDH2* | CCT CCA GAG TTT ACT GCC ATG AC | GTA GGA TCT CCG CCA CTG ATT C |
| *CLDN3* | GCC TTC ATC GGC AGC AAC ATC A | AGC GAG TCG TAC ACC TTG CAC T |
| *CLDN4* | AGT GCA AGG TGT ACG ACT CGC T | CGC TTT CAT CCT CCA GGC AGT T |
| *CLDN7* | GGA GAC GAC AAA GTG AAG AAG GC | AAA AGT CTG TGA CAA TCT GAT GGC |
| *GAPDH* | CAT CCA TGA CAA CTT TGG TAT CGT | CCA TCA CGC CAC AGT TTC C |
| *HES1* | CAA GAC CAA AGC GGA AAG AA | GGA TCC TGT GTG ATC CCT AGG C |
| *HES4* | GAG CGC GTA TTA ACG AGA GCC T | CTC ACG GTC ATC TCC AGG ATG T |
| *HES5* | TCC TGG AGA TGG CTG TCA GCT A | CGT GGA GCG TCA GGA ACT GCA |
| *HEY2* | TGA GAA GAC TTG TGC CAA CTG CT | CCC TGT TGC CTG AAG CAT CTT C |
| *HEYL* | TGG AGA AAG CCG AGG TCT TGC A | ACC TGA TGA CCT CAG TGA GGC A |
| *HPRT* | TGA CAC TGG CAA AAC AAT GCA | GGT CCT TTT CAC CAG CAA GCT |
| *ITGA6* | CGA AAC CAA GGT TCT GAG CCC A | CTT GGA TCT CCA CTG AGG CAG T |
| *MUC1* | CCT ACC ATC CTA TGA GCG AGT AC | GCT GGG TTT GTG TAA GAG AGG C |
| *MYC* | CTT CTC TCC GTC CTC GGA TTC | GAA GGT GAT CCA GAC TCT GAC CTT |
| *NOTCH1* | TGT GTC TGC CGA CGC ACA AGG T | AGG CGG GCA GCC AGG ATC AGT |
| *SOX2* | GCT ACA GCA TGA TGC AGG ACC A | TCT GCG AGC TGG TCA TGG AGT T |
| *TBP* | GCC CGA AAC GCC GAA TAT A | CGT GGC TCT CTT ATC CTC ATG A |
| *SNAI2* | ATC TGC GGC AAG GCG TTT TCC A | GAG CCC TCA GAT TTG ACC TGT C |
| *ZEB2* | AAT GCA CAG AGT GTG GCA AGG C | CTG CTG ATG TGC GAA CTG TAG G |
|  |  |  |

| ChIP-qPCR Primers | | |
| --- | --- | --- |
| Peak | Forward | Reverse |
| *HES1 peak1* | CCT AGG GGA CAA AGG GGA GT | AC CGC ATC ACC TGT TTG CT |
| *HES1 peak2* | CTT TCG GCC GAG TGA AAA CC | CAG GCC AAG GTC AGC TCT TC |
| *NOTCH1 peak1* | GAC ACC CAA TAC CTG CCT CC | CTT CCG AAT GGG GGA CCT TC |
| *NOTCH1 peak2* | TCT GGG AGA GTG GCC TAG C | TCG ATC CTC TGG ACG CCT AA |
