# Supplementary material for: Reciprocal inhibition of NOTCH and SOX2 shapes tumor cell plasticity and therapeutic escape in triple-negative breast cancer
Source: EMBO Mol Med. 2024 Oct 30;16(12):9. doi: 10.1038/s44321-024-00161-8 (PMC11628624; doi:10.1038/s44321-024-00161-8)
Supplement: Supplementary file 3 — Appendix [file 44321_2024_161_MOESM3_ESM.pdf]

Appendix

Table of content

Page 1	Table of content
Page 2	Appendix Figure S1
Page 3	Appendix Figure S1
Page 4	Appendix Figure S2

Appendix Figure S1

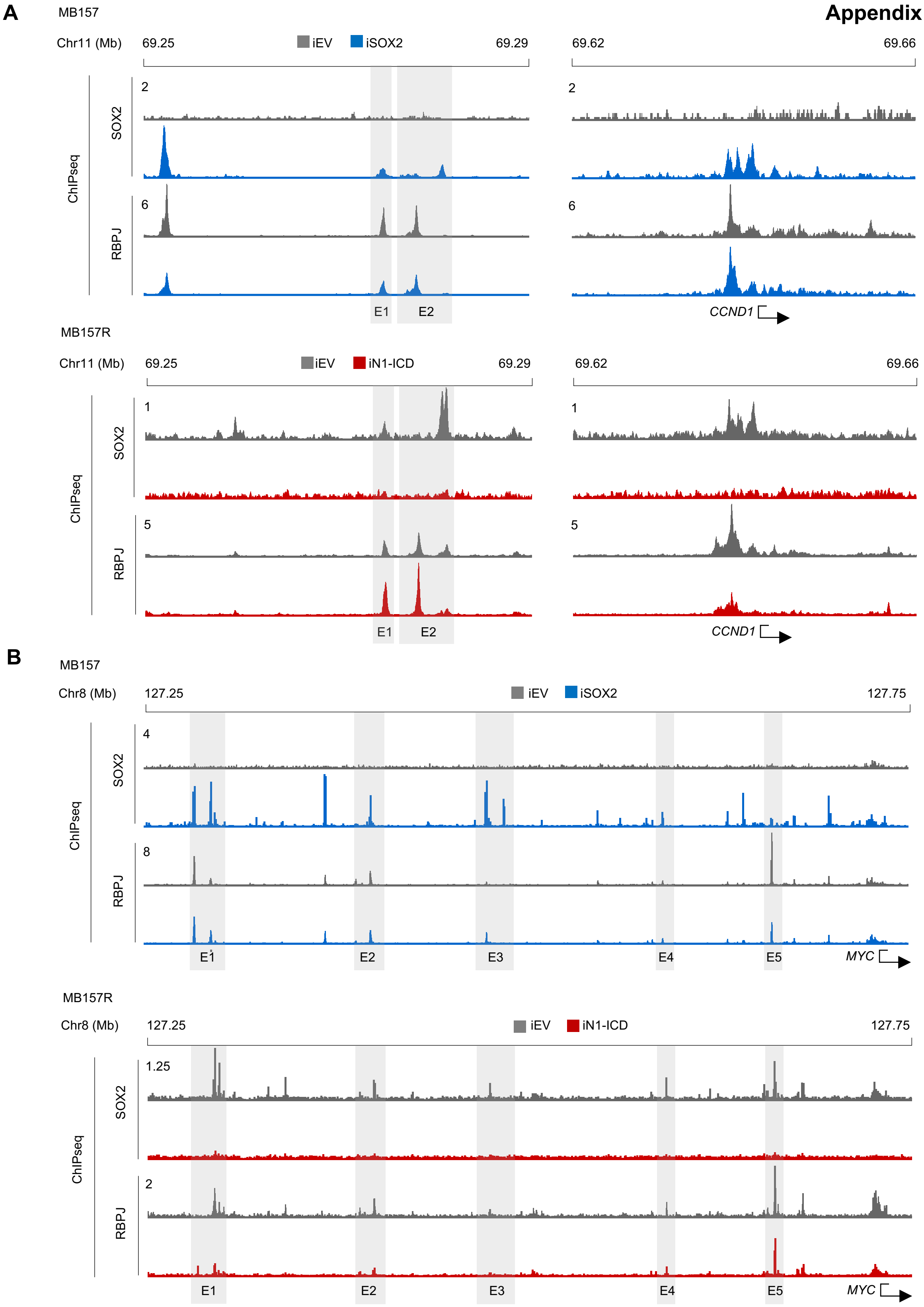

Appendix Figure S1: SOX2 interferes with the RBP-J activation complex and represses NOTCH1 and NOTCH target genes

(A) ChIP peaks for HA-tagged SOX2 or SOX2 and RBPJ on CCND1 enhancers and promoter and (B) on MYC enhancers and promoter in iSOX2 or iEV control MB157 and iN1-ICD or iEV control MB157R cells, 72 hours after DOX induction. The y-axis represents reads per million mapped reads.

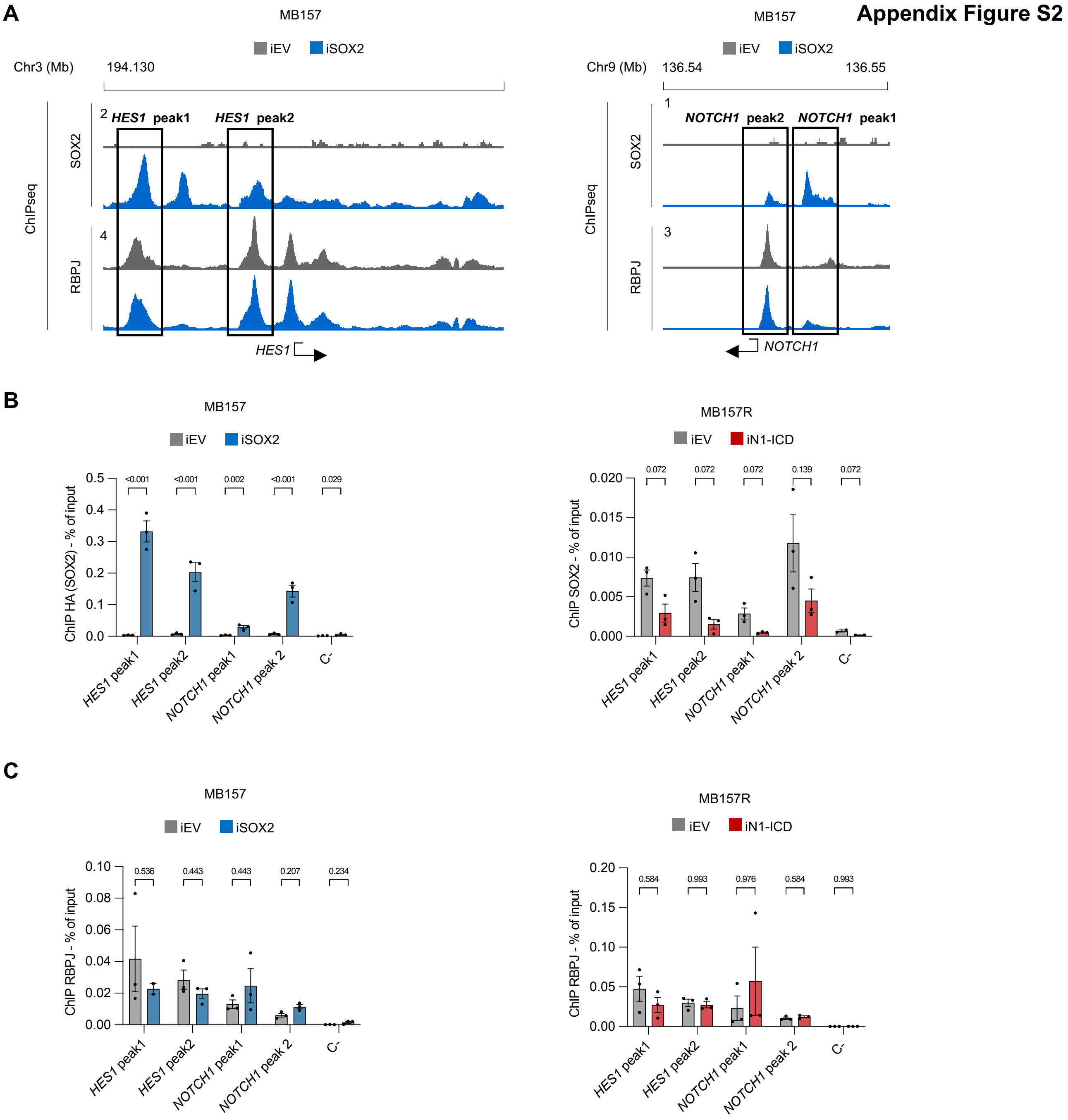

Appendix Figure S2: SOX2 interferes with the RBP-J activation complex and represses NOTCH1 and NOTCH target genes

(A) ChIPseq for HA-tagged SOX2 or SOX2 and RBPJ on HES1 and NOTCH1 with annotations of the peaks validated, (B) ChIPqpcr for HA-tagged SOX2 or SOX2 and RBPJ on HES1 and (C) on NOTCH1 promoter in iSOX2 or iEV control MB157 and iN1-ICD or iEV control MB157R cells, 72 hours after DOX induction.
