# Supplementary figures and images for: Reciprocal inhibition of NOTCH and SOX2 shapes tumor cell plasticity and therapeutic escape in triple-negative breast cancer
Source: EMBO Mol Med. 2024 Oct 30;16(12):9. doi: 10.1038/s44321-024-00161-8 (PMC11628624; doi:10.1038/s44321-024-00161-8)

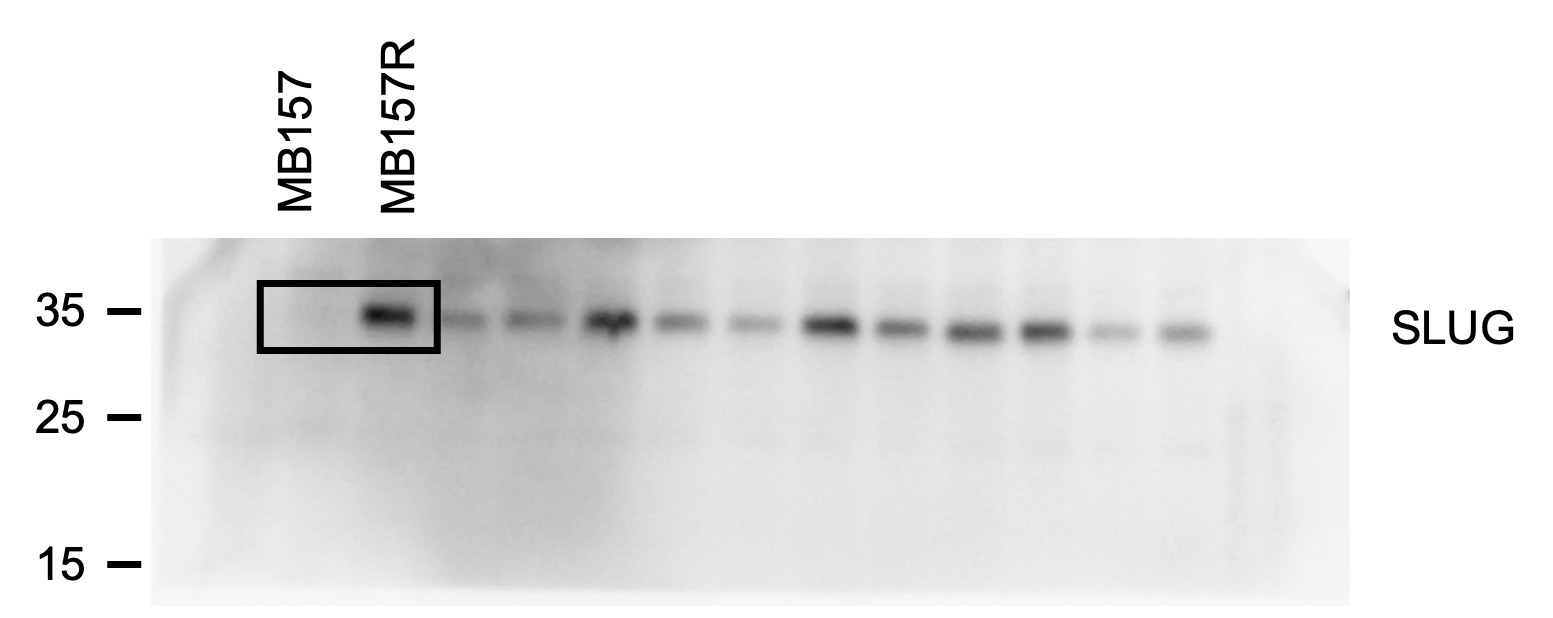

Supplement: Supplementary file 9 — Source data Fig. 1 [file 44321_2024_161_MOESM9_ESM.zip › Fig1/1L/western MB157 MB157R SLUG.jpg]

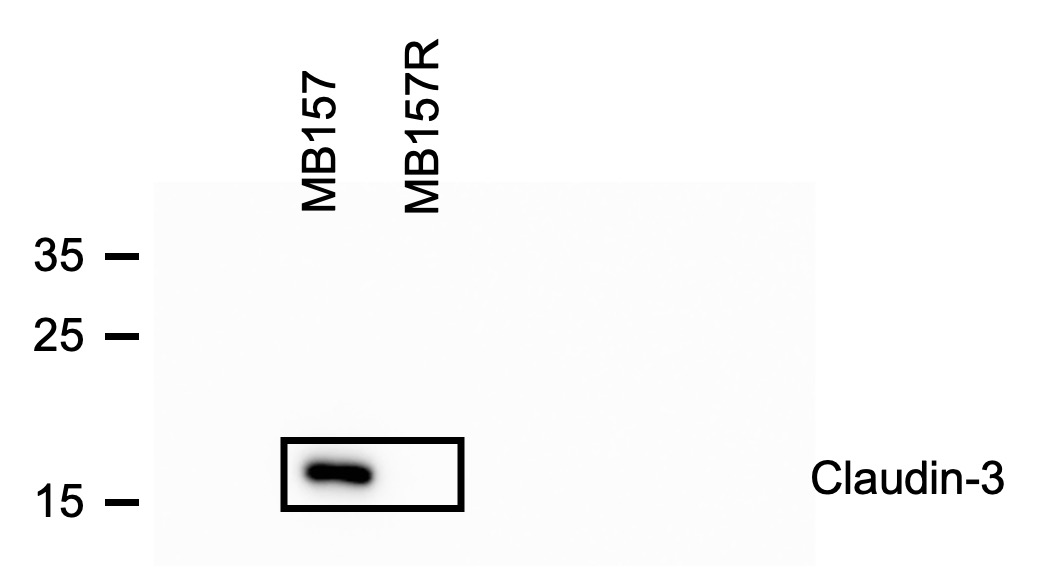

Supplement: Supplementary file 9 — Source data Fig. 1 [file 44321_2024_161_MOESM9_ESM.zip › Fig1/1L/western MB157 MB157R Claudin-3.jpg]

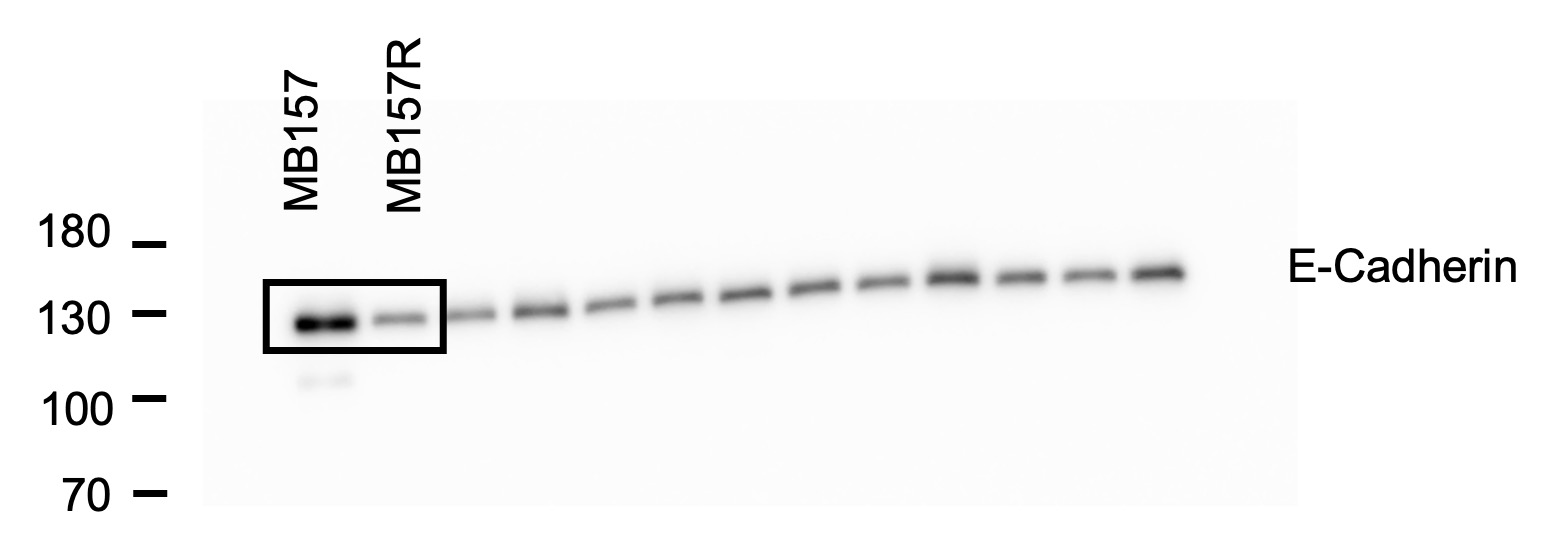

Supplement: Supplementary file 9 — Source data Fig. 1 [file 44321_2024_161_MOESM9_ESM.zip › Fig1/1L/western MB157 MB157R E-cadherin.jpg]

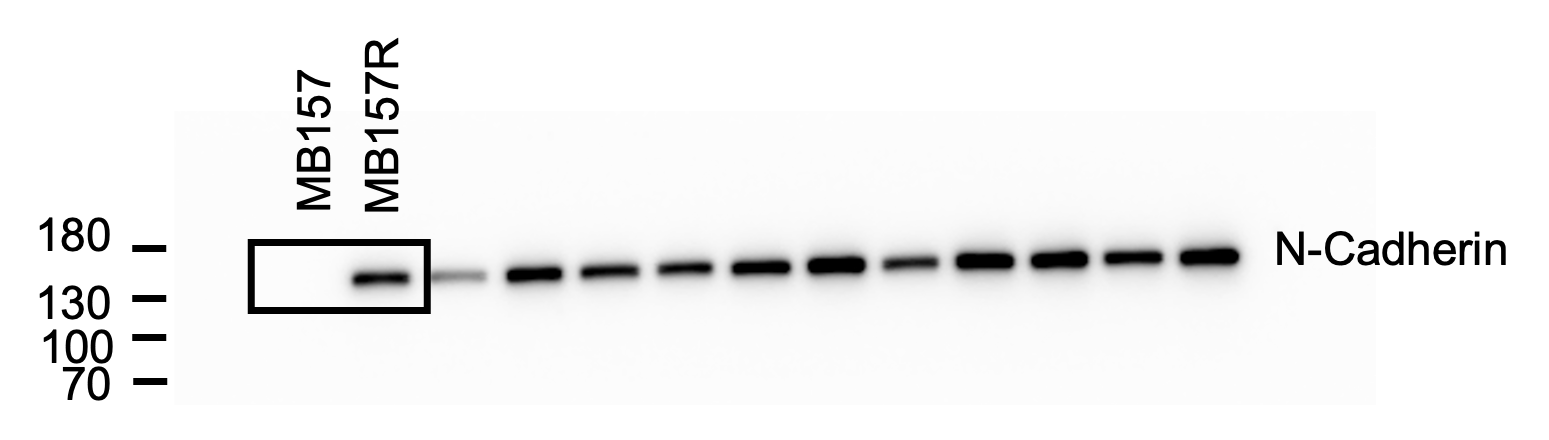

Supplement: Supplementary file 9 — Source data Fig. 1 [file 44321_2024_161_MOESM9_ESM.zip › Fig1/1L/western MB157 MB157R N-cadherin.png]

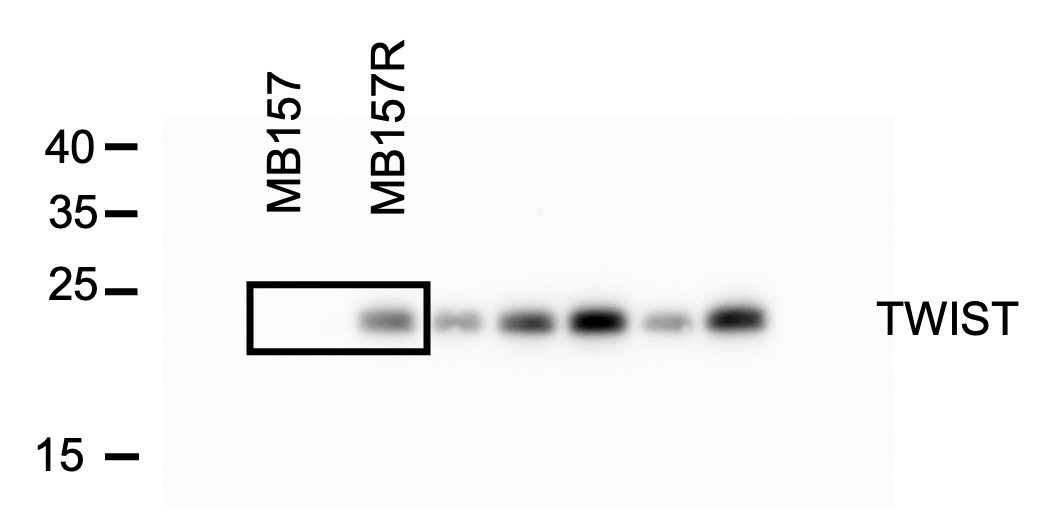

Supplement: Supplementary file 9 — Source data Fig. 1 [file 44321_2024_161_MOESM9_ESM.zip › Fig1/1L/western MB157 MB157R TWIST.jpg]

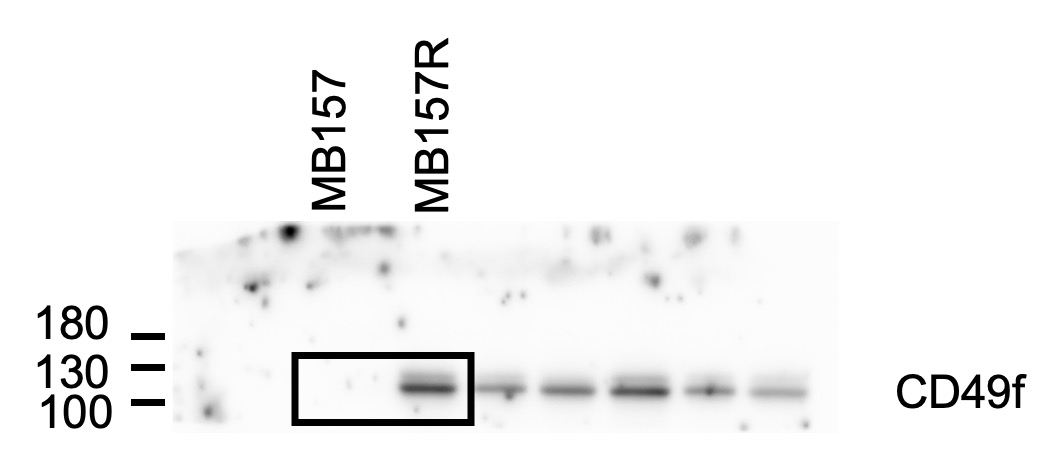

Supplement: Supplementary file 9 — Source data Fig. 1 [file 44321_2024_161_MOESM9_ESM.zip › Fig1/1L/western MB157 MB157R CD49f.jpg]

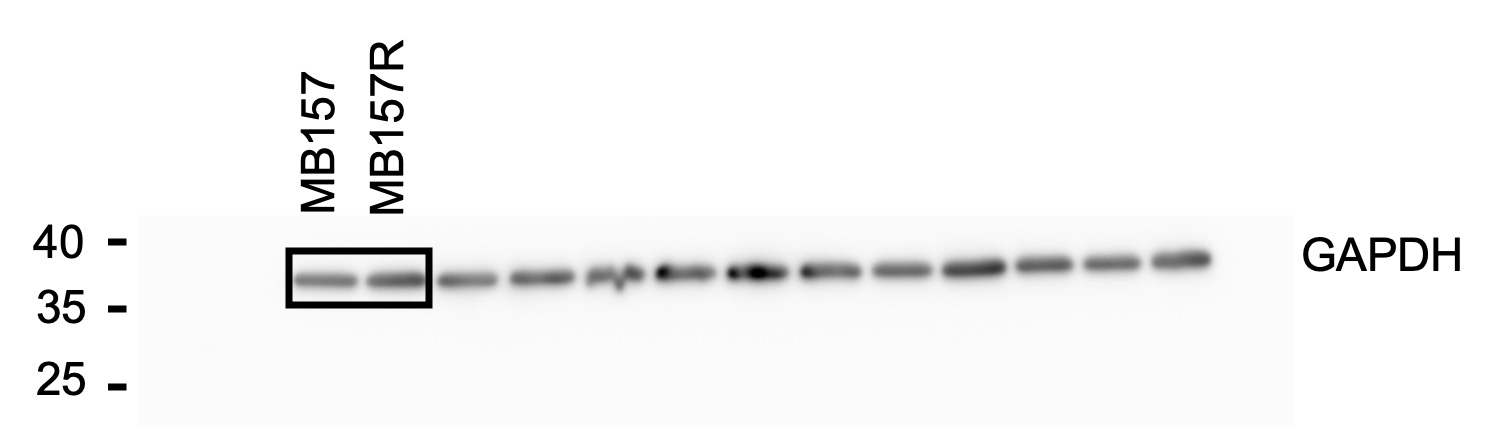

Supplement: Supplementary file 9 — Source data Fig. 1 [file 44321_2024_161_MOESM9_ESM.zip › Fig1/1L/western MB157 MB157R GAPDH.jpg]

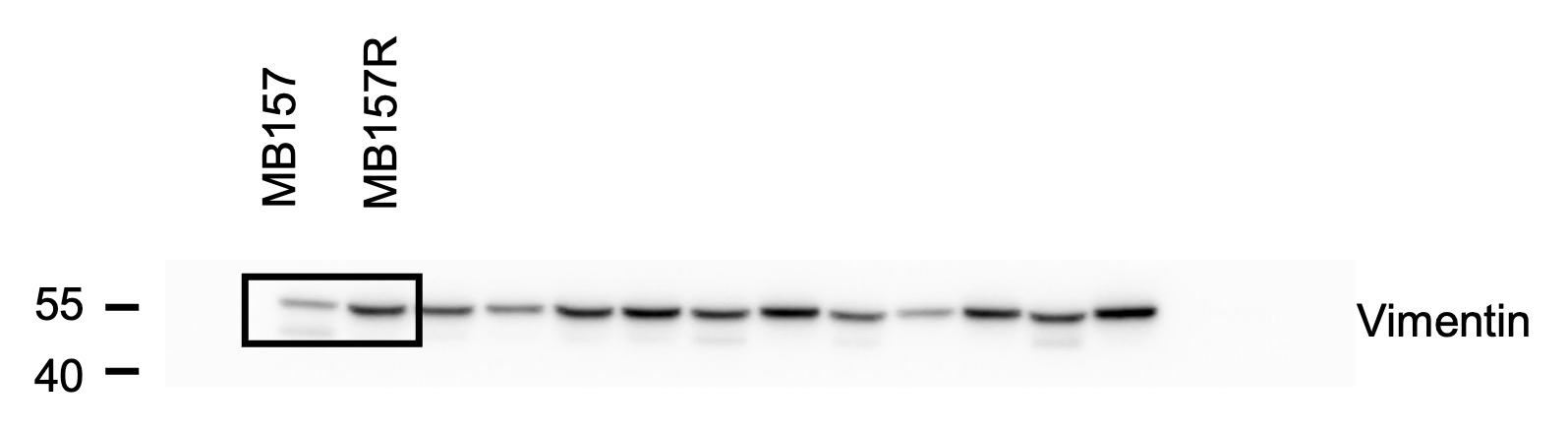

Supplement: Supplementary file 9 — Source data Fig. 1 [file 44321_2024_161_MOESM9_ESM.zip › Fig1/1L/western MB157 MB157R Vimentin.jpg]

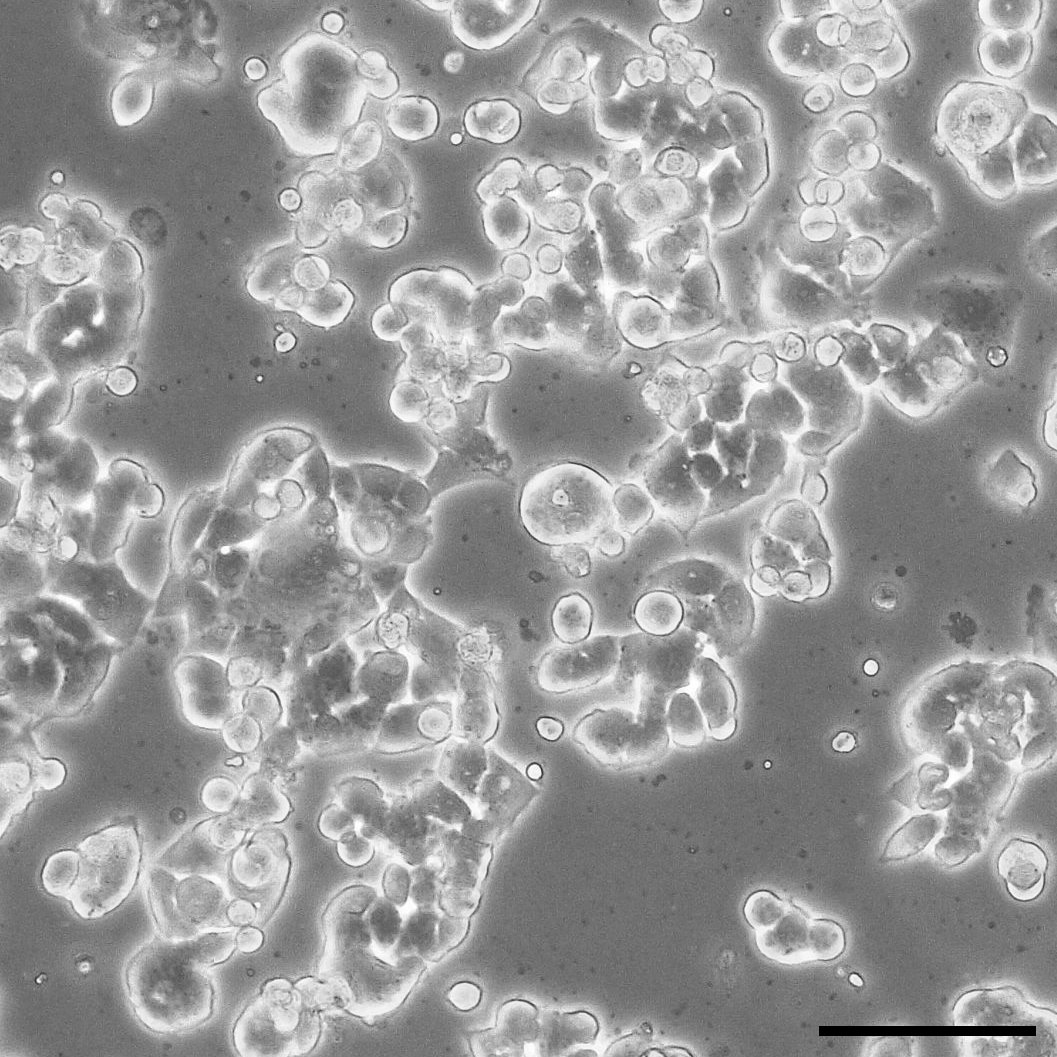

Supplement: Supplementary file 9 — Source data Fig. 1 [file 44321_2024_161_MOESM9_ESM.zip › Fig1/1J/cell pictures MB157.jpg]

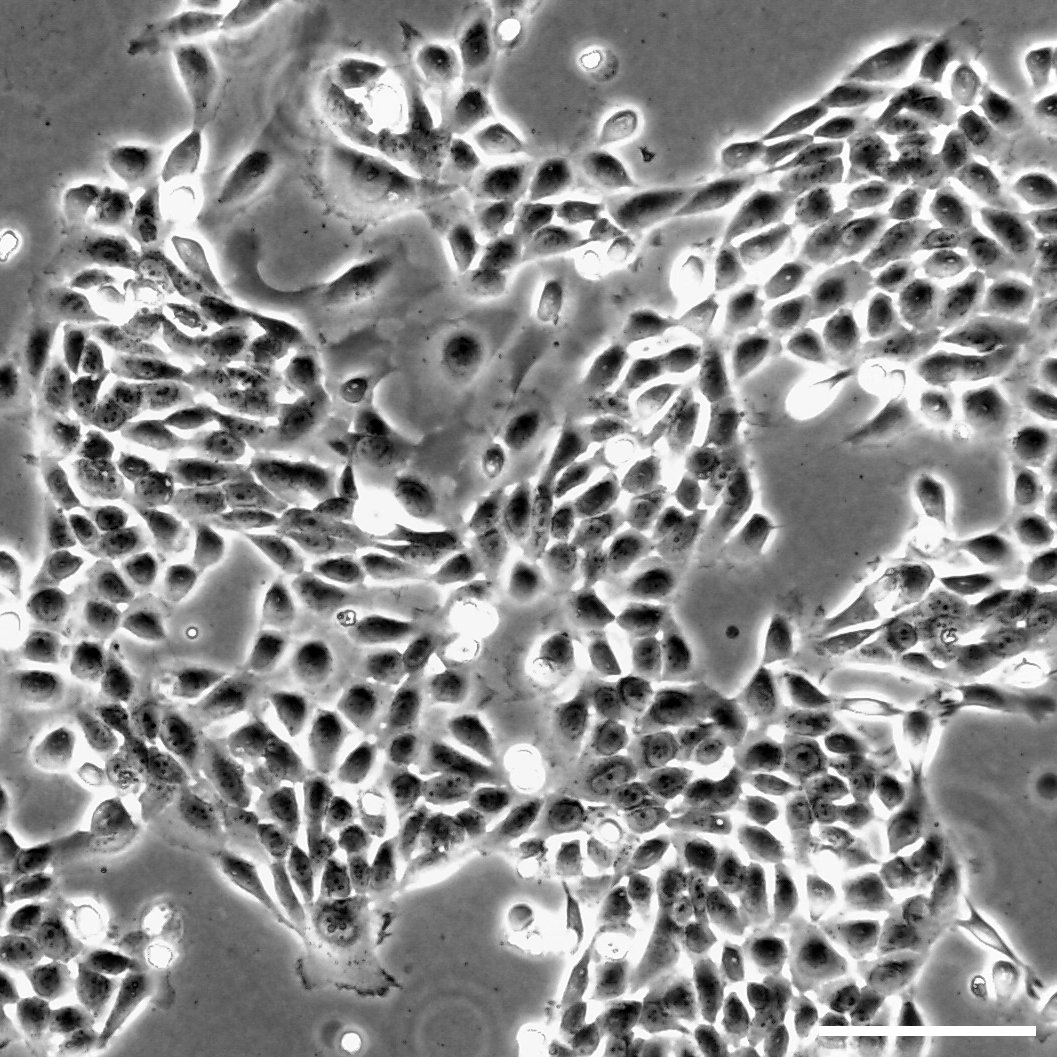

Supplement: Supplementary file 9 — Source data Fig. 1 [file 44321_2024_161_MOESM9_ESM.zip › Fig1/1J/cell pictures MB157R.jpg]

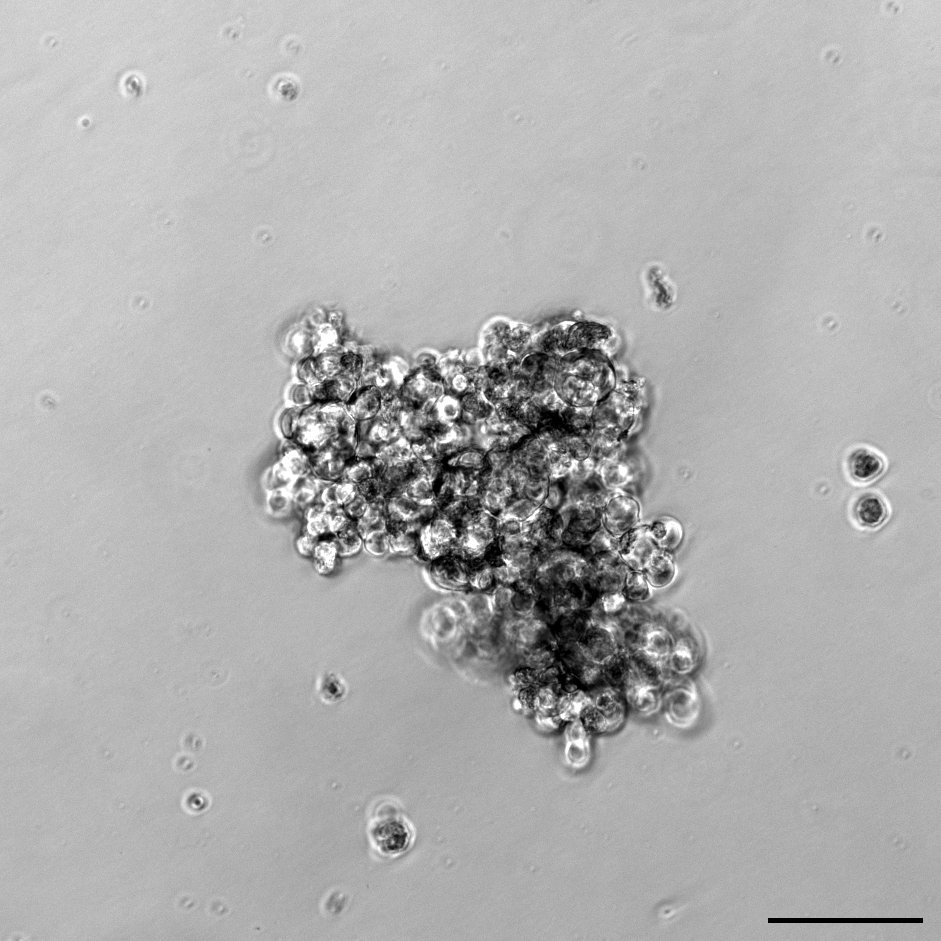

Supplement: Supplementary file 9 — Source data Fig. 1 [file 44321_2024_161_MOESM9_ESM.zip › Fig1/1N/Tumorsphere MB157.jpg]

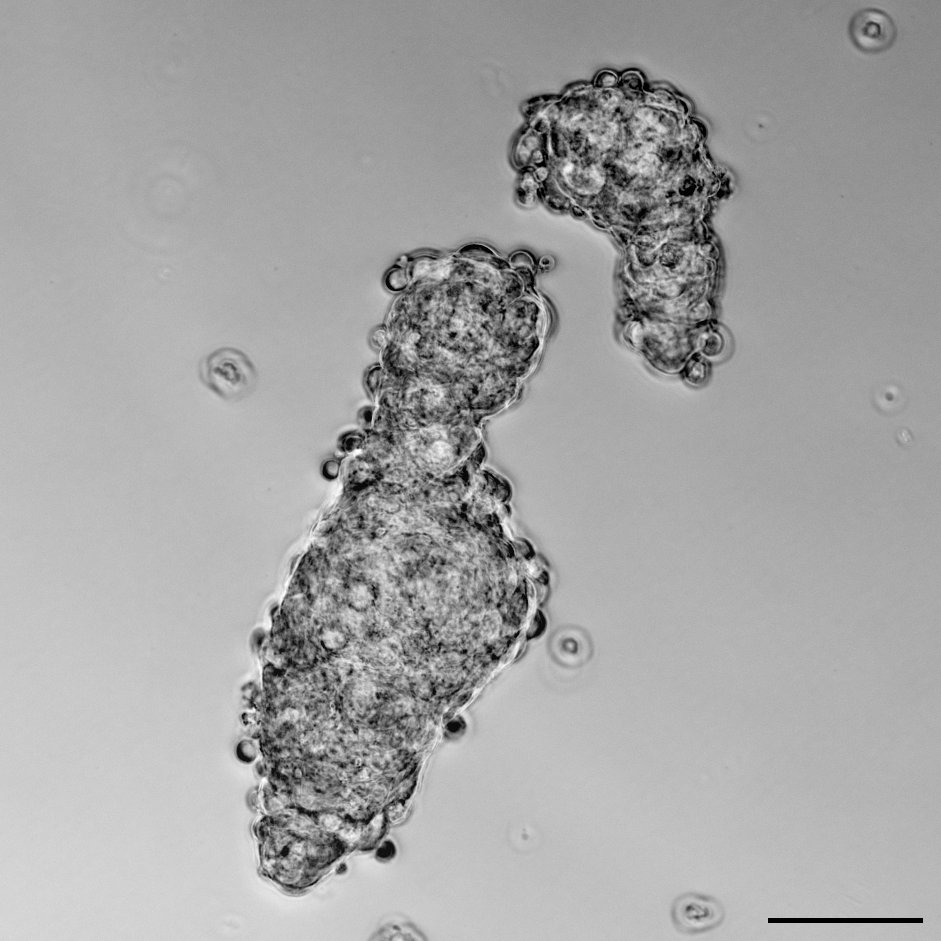

Supplement: Supplementary file 9 — Source data Fig. 1 [file 44321_2024_161_MOESM9_ESM.zip › Fig1/1N/Tumorsphere MB157R.jpg]

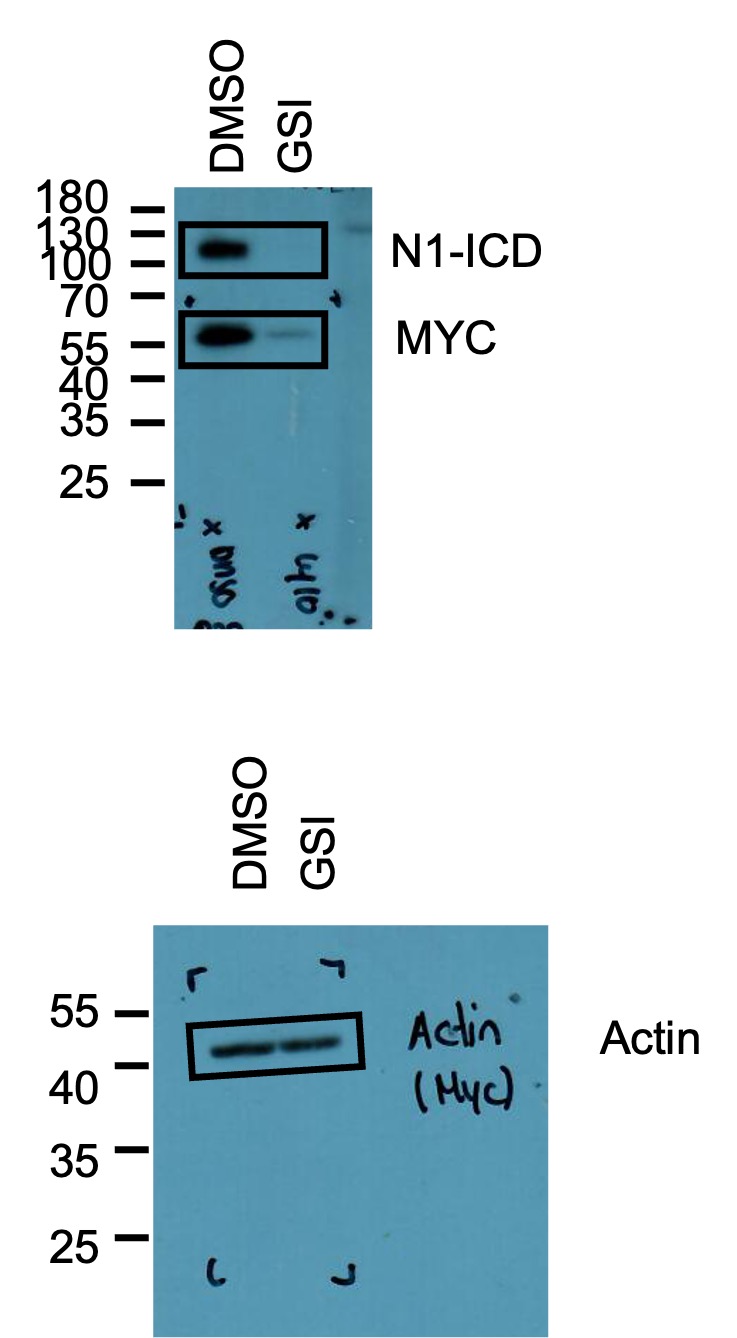

Supplement: Supplementary file 9 — Source data Fig. 1 [file 44321_2024_161_MOESM9_ESM.zip › Fig1/1E/western MB157 N1-ICD MYC Actin.jpg]

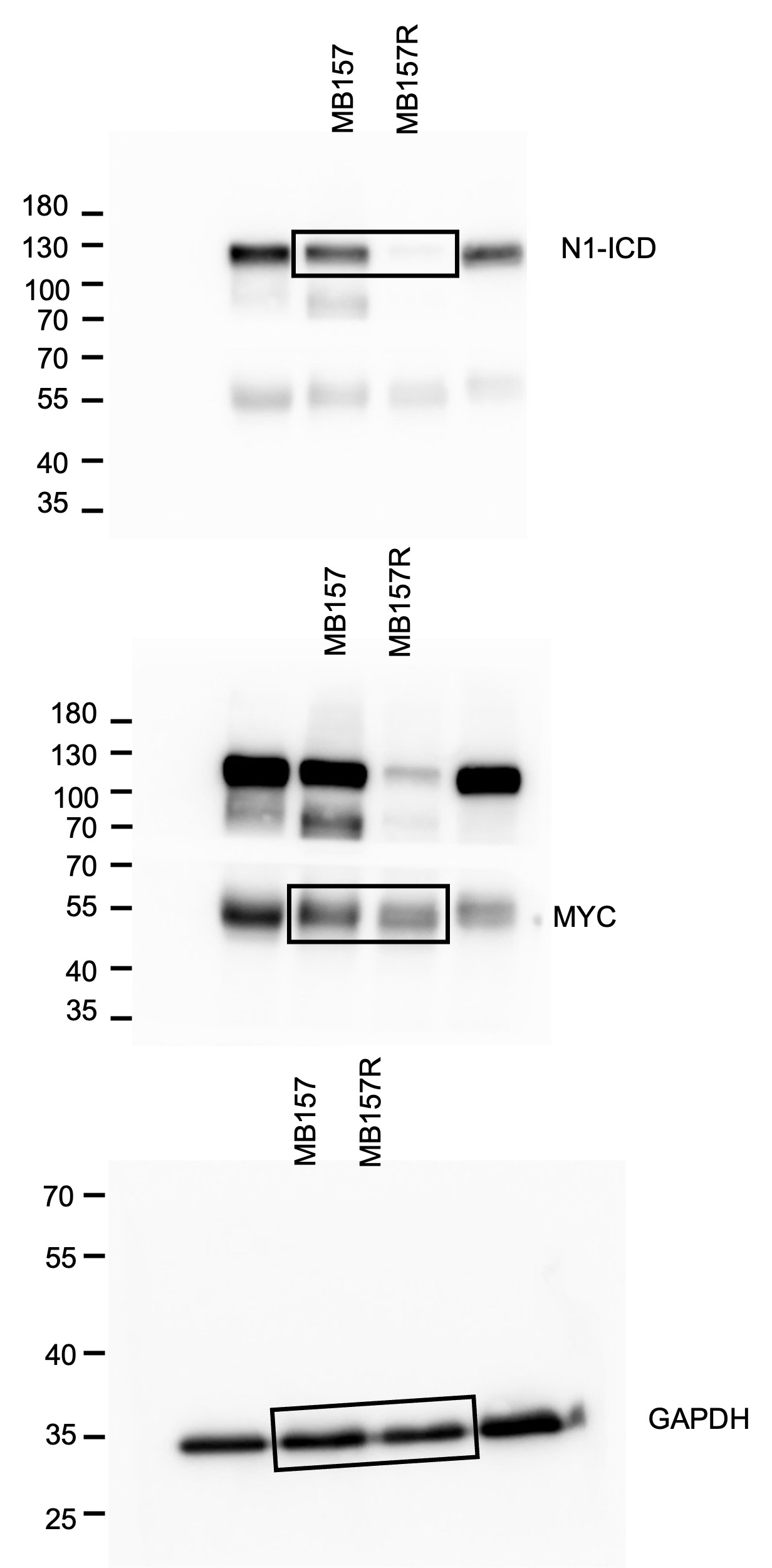

Supplement: Supplementary file 9 — Source data Fig. 1 [file 44321_2024_161_MOESM9_ESM.zip › Fig1/1I/western MB157 MB157R N1-ICd MYC GAPDH.jpg]

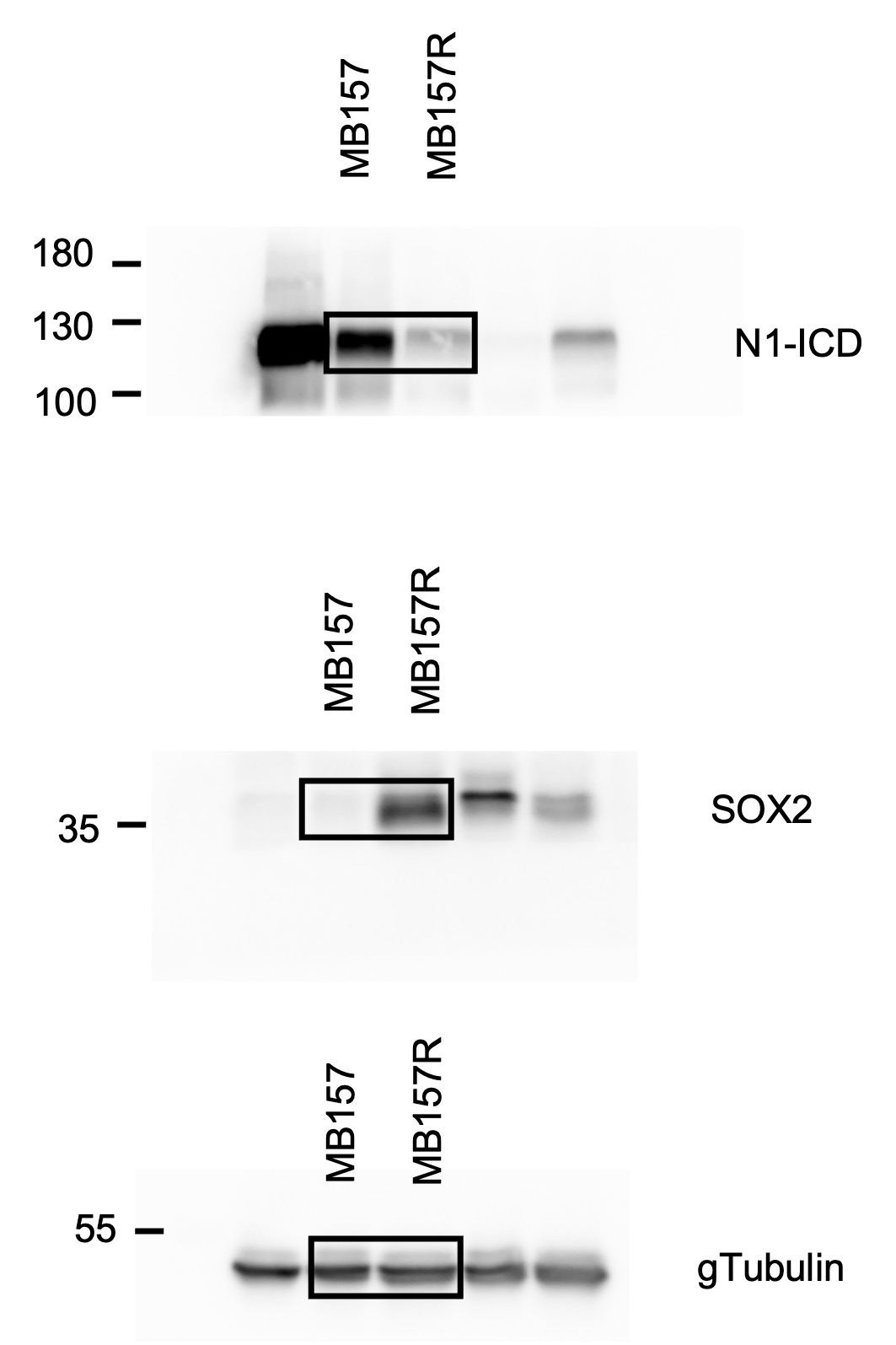

Supplement: Supplementary file 10 — Source data Fig. 2 [file 44321_2024_161_MOESM10_ESM.zip › Fig2/2C/western Mb157 MB157R N1-ICD SOX2 gTubulin.jpg]

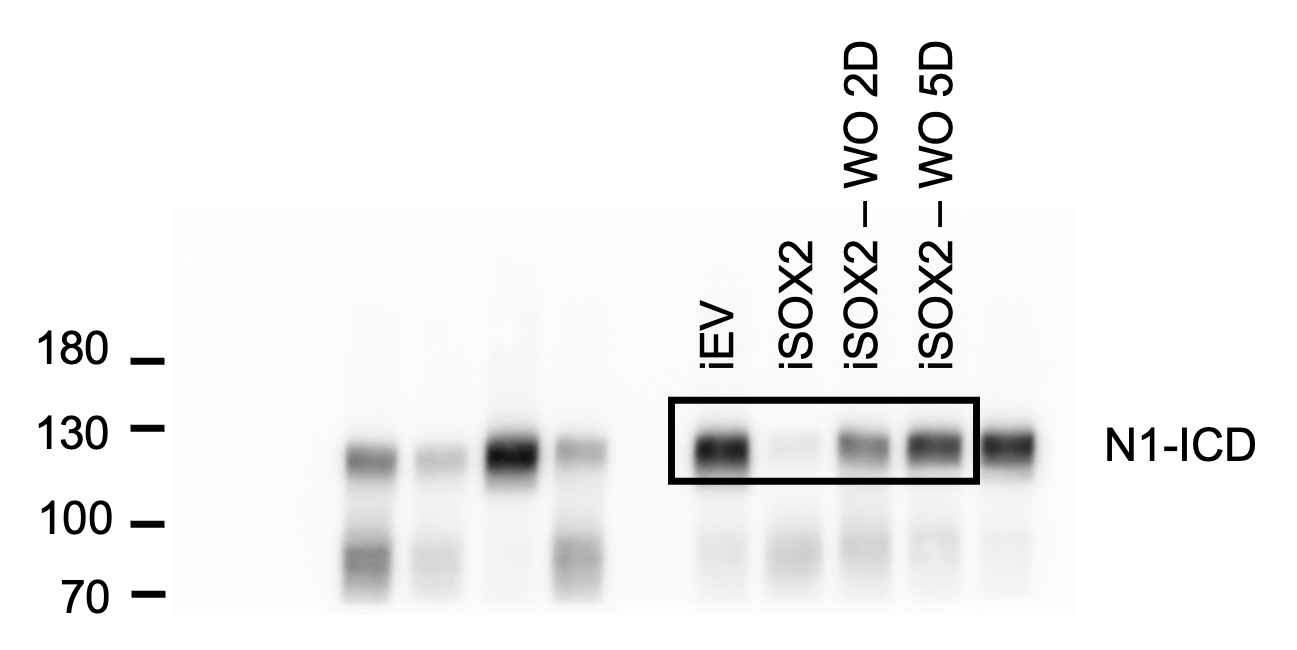

Supplement: Supplementary file 10 — Source data Fig. 2 [file 44321_2024_161_MOESM10_ESM.zip › Fig2/2F/Western MB157 iSOX2 N1-ICD.jpg]

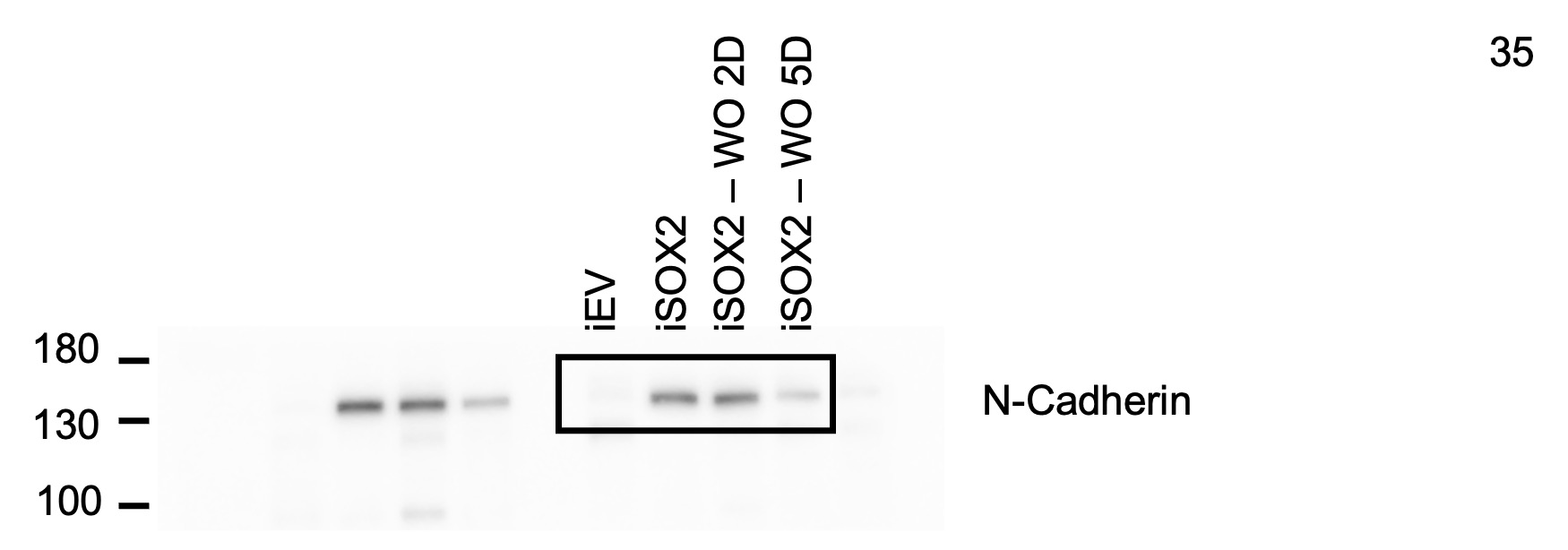

Supplement: Supplementary file 10 — Source data Fig. 2 [file 44321_2024_161_MOESM10_ESM.zip › Fig2/2F/Western MB157 iSOX2 N-cadherin.jpg]

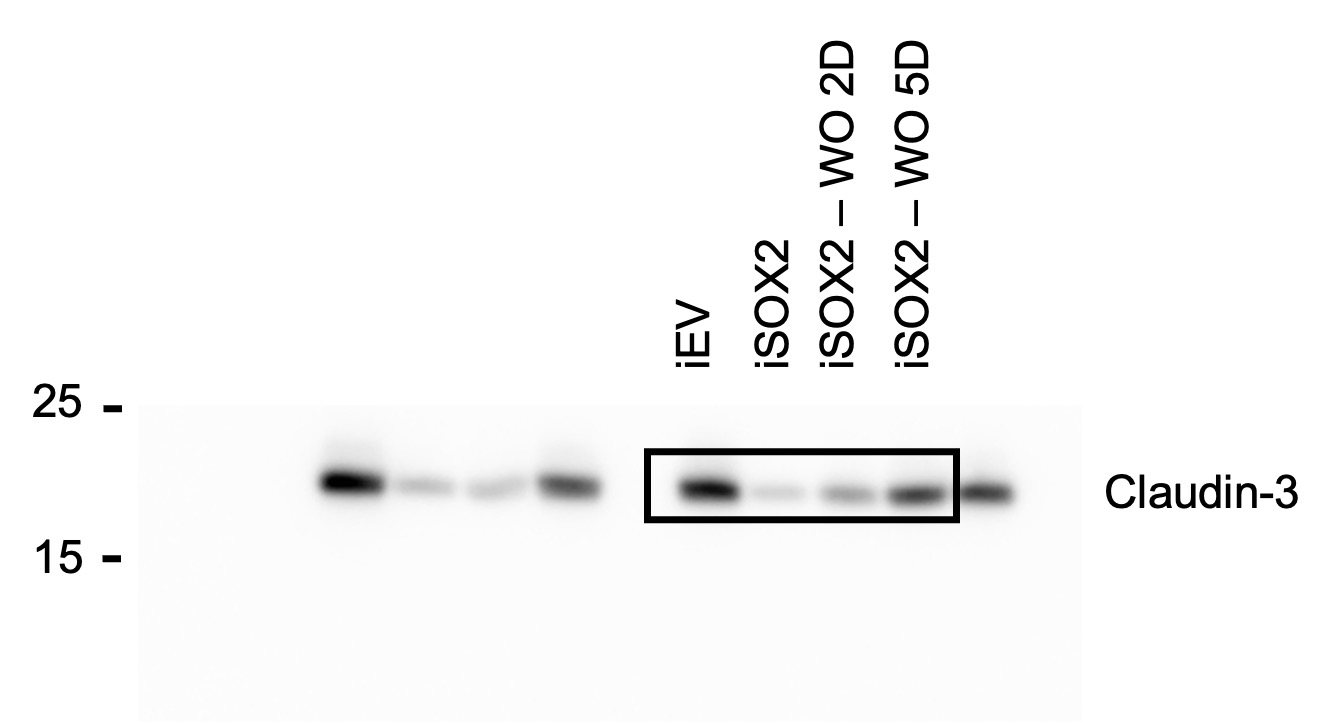

Supplement: Supplementary file 10 — Source data Fig. 2 [file 44321_2024_161_MOESM10_ESM.zip › Fig2/2F/Western MB157 iSOX2 Claudin-3.jpg]

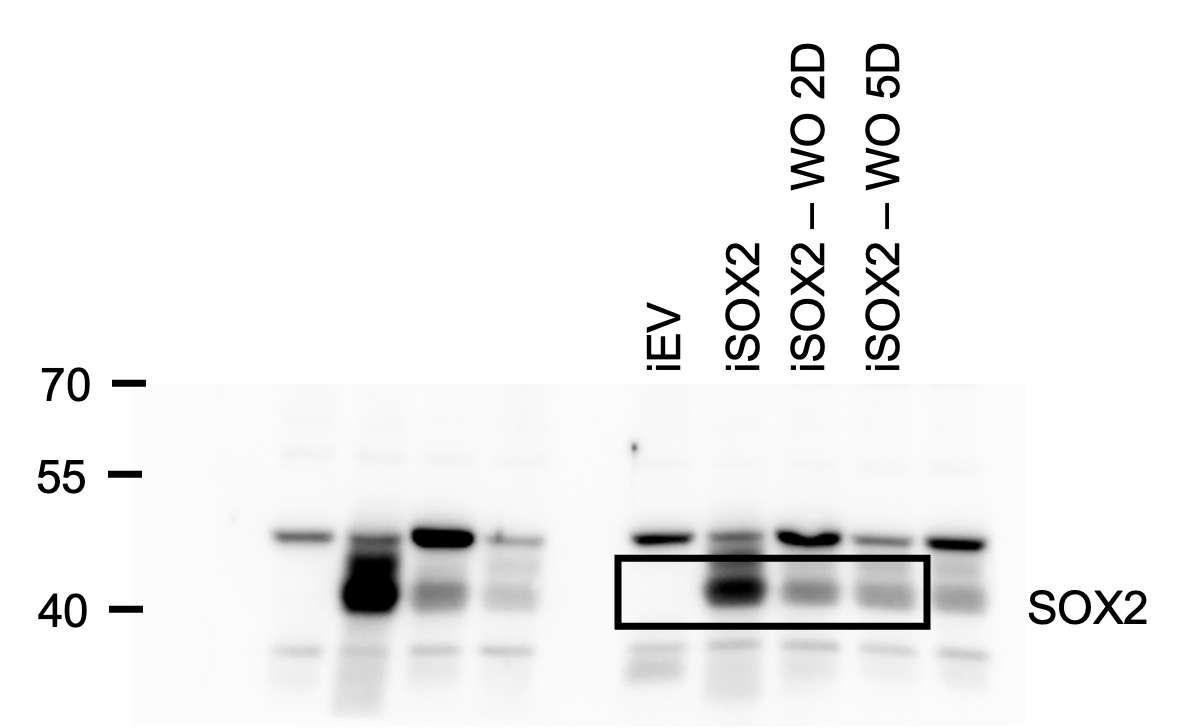

Supplement: Supplementary file 10 — Source data Fig. 2 [file 44321_2024_161_MOESM10_ESM.zip › Fig2/2F/Western MB157 iSOX2 SOX2.jpg]

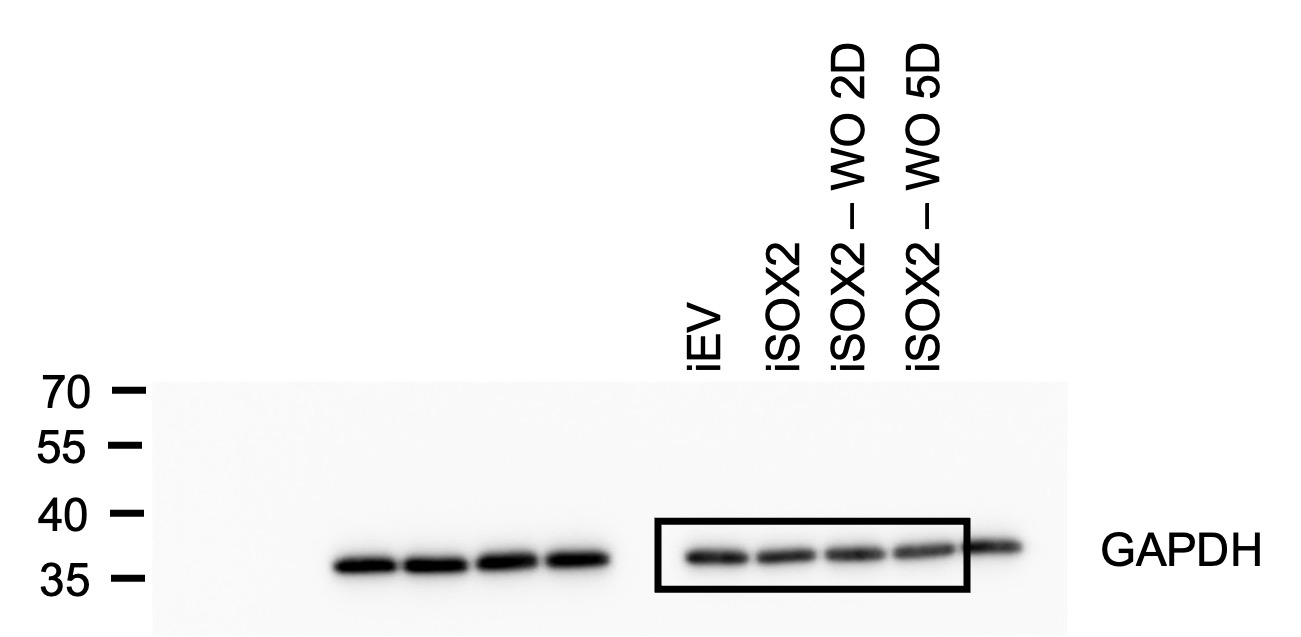

Supplement: Supplementary file 10 — Source data Fig. 2 [file 44321_2024_161_MOESM10_ESM.zip › Fig2/2F/Western MB157 iSOX2 GAPDH.jpg]

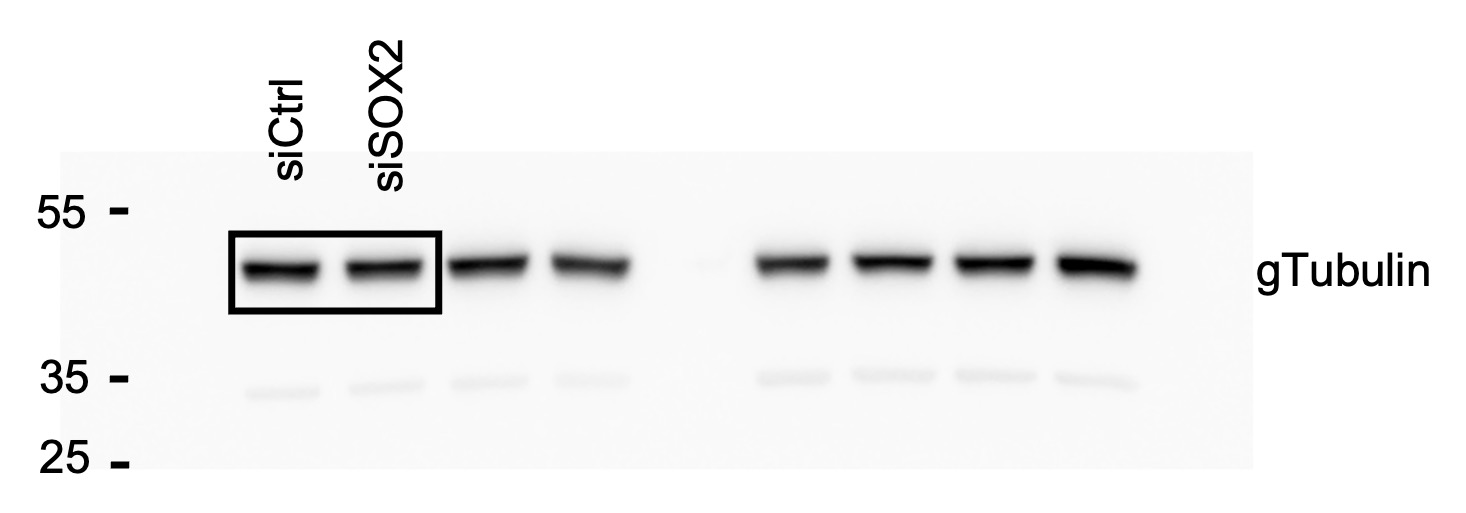

Supplement: Supplementary file 10 — Source data Fig. 2 [file 44321_2024_161_MOESM10_ESM.zip › Fig2/2E/western MB157R siSOX2 gTubulin.jpg]

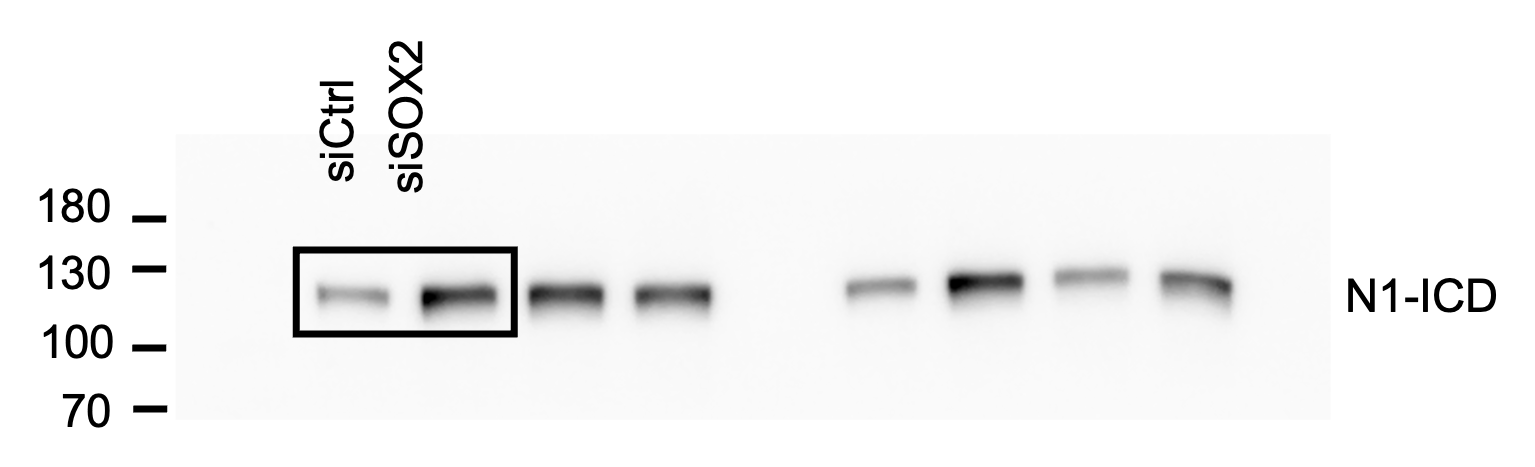

Supplement: Supplementary file 10 — Source data Fig. 2 [file 44321_2024_161_MOESM10_ESM.zip › Fig2/2E/western MB157R siSOX2 N1-ICD.png]

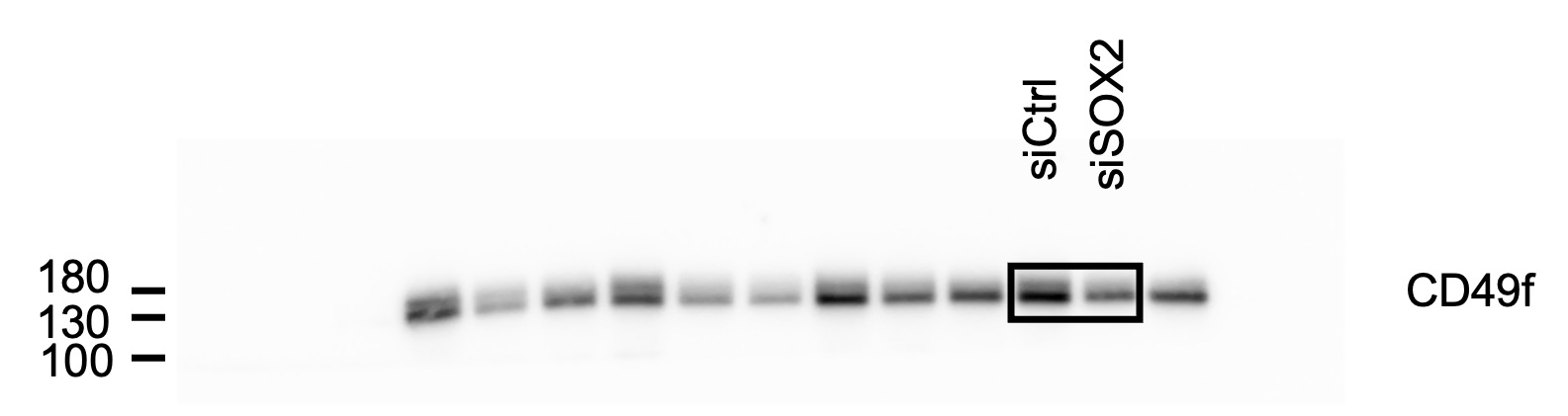

Supplement: Supplementary file 10 — Source data Fig. 2 [file 44321_2024_161_MOESM10_ESM.zip › Fig2/2E/western MB157R siSOX2 CD49f.jpg]

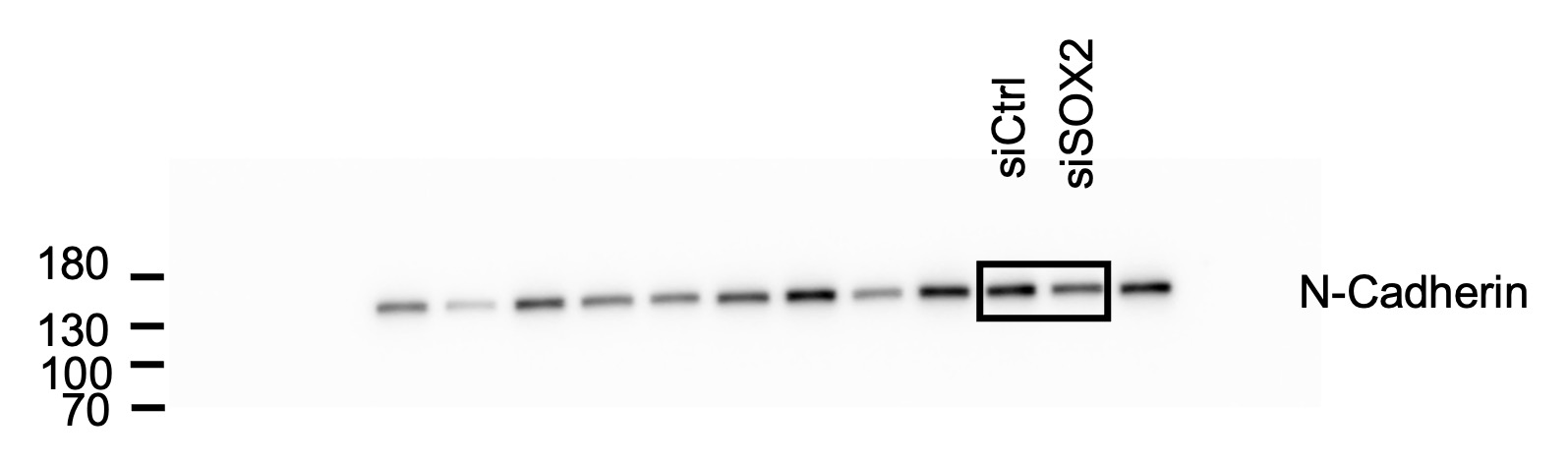

Supplement: Supplementary file 10 — Source data Fig. 2 [file 44321_2024_161_MOESM10_ESM.zip › Fig2/2E/western MB157R siSOX2 N-Cadherin.jpg]

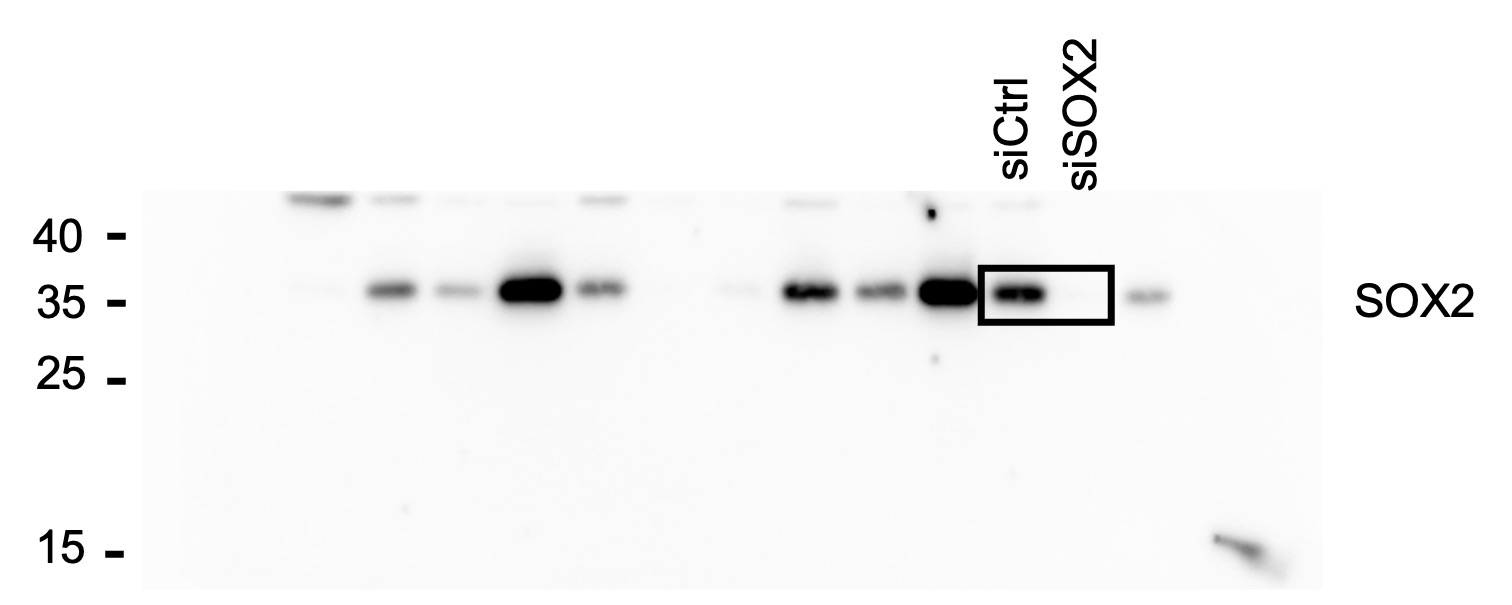

Supplement: Supplementary file 10 — Source data Fig. 2 [file 44321_2024_161_MOESM10_ESM.zip › Fig2/2E/western MB157R siSOX2 SOX2.jpg]

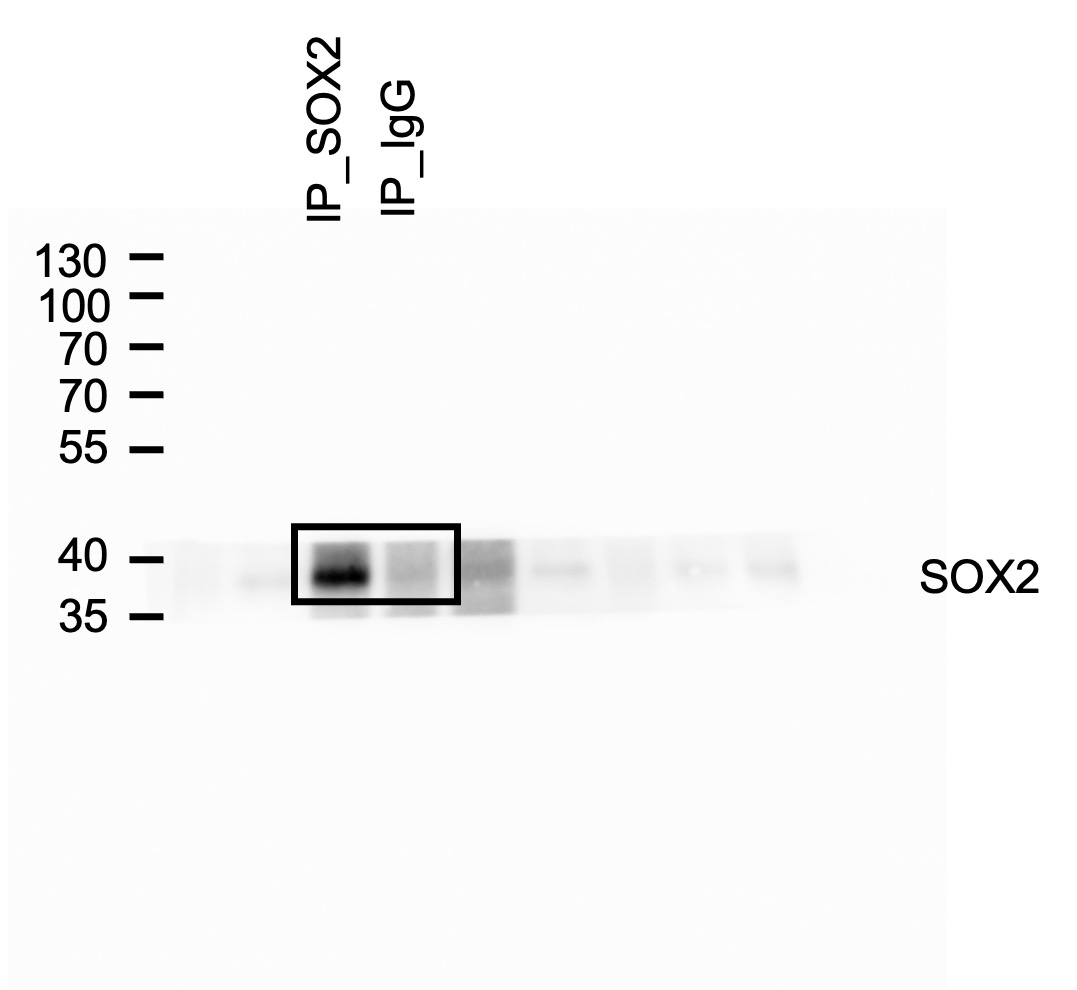

Supplement: Supplementary file 11 — Source data Fig. 3 [file 44321_2024_161_MOESM11_ESM.zip › Fig3/3E/IP_SOX2 MB157R SOX2.jpg]

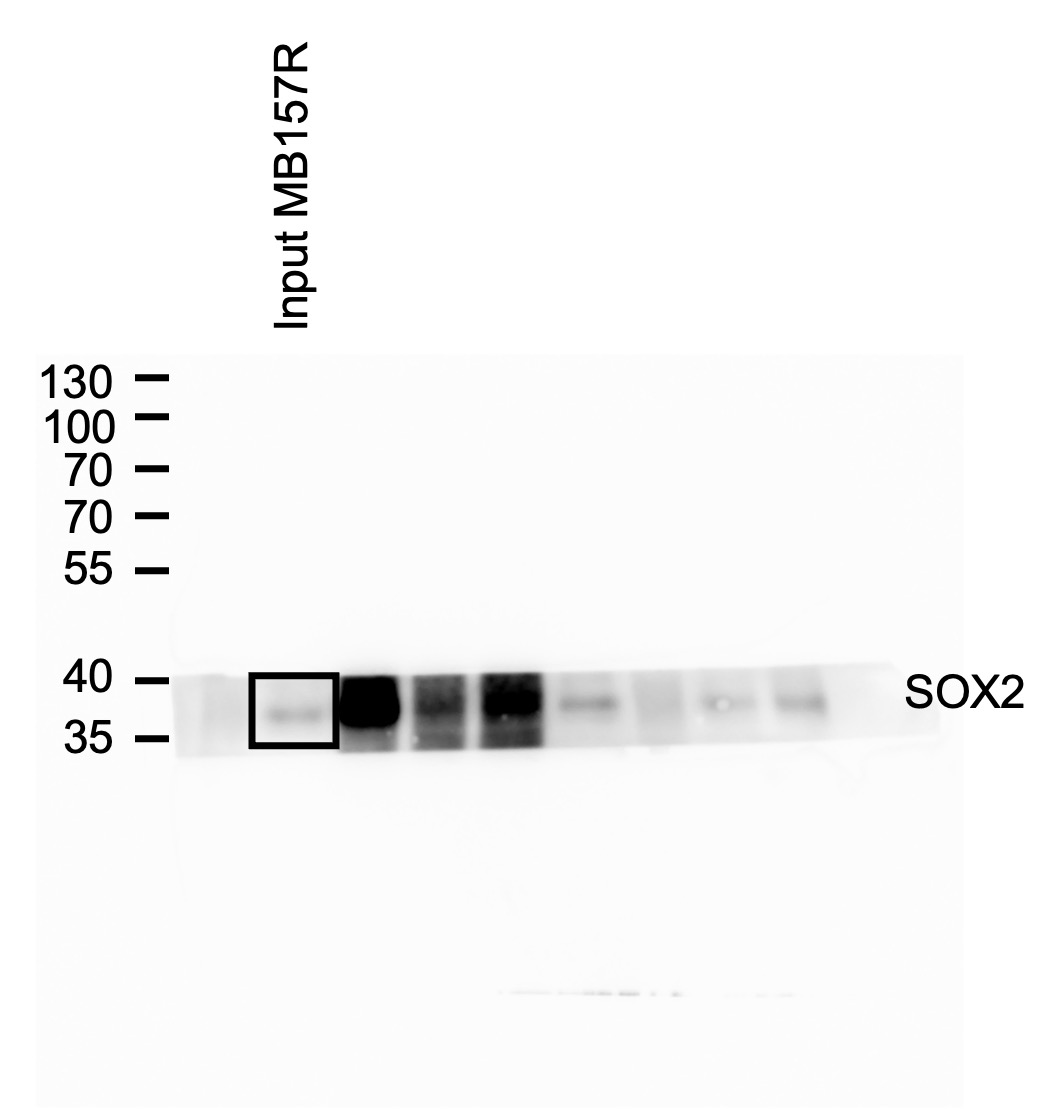

Supplement: Supplementary file 11 — Source data Fig. 3 [file 44321_2024_161_MOESM11_ESM.zip › Fig3/3E/IP_input MB157R SOX2.jpg]

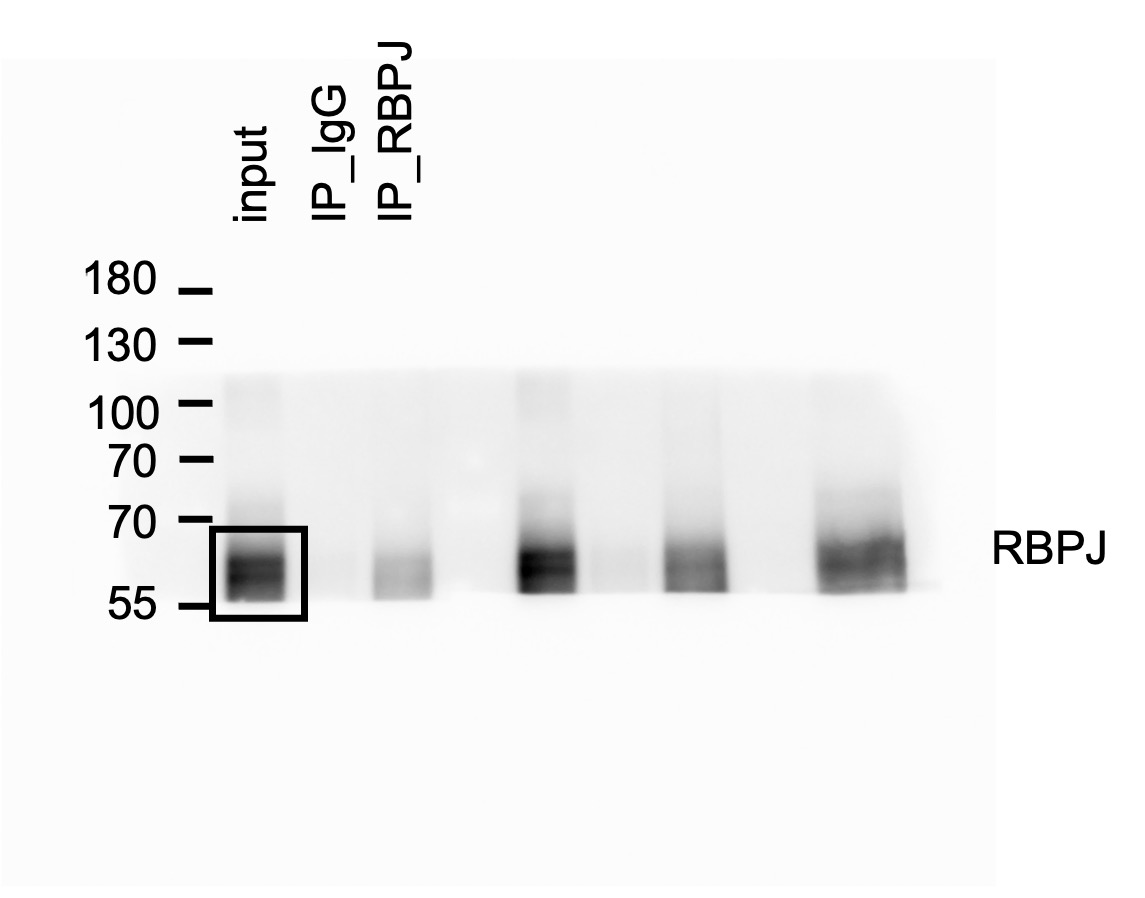

Supplement: Supplementary file 11 — Source data Fig. 3 [file 44321_2024_161_MOESM11_ESM.zip › Fig3/3E/IP_input MB157iSOX2 RBPJ.jpg]

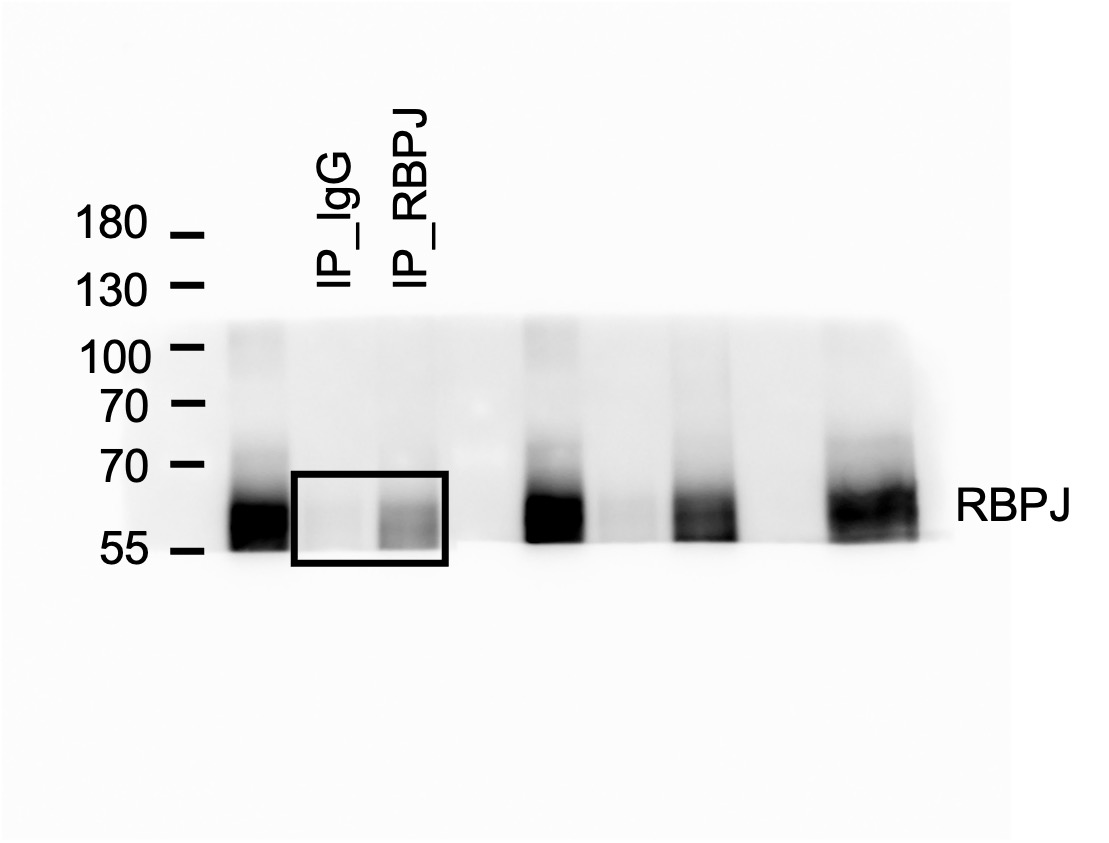

Supplement: Supplementary file 11 — Source data Fig. 3 [file 44321_2024_161_MOESM11_ESM.zip › Fig3/3E/IP_RBPJ MB157iSOX2 RBPJ.jpg]

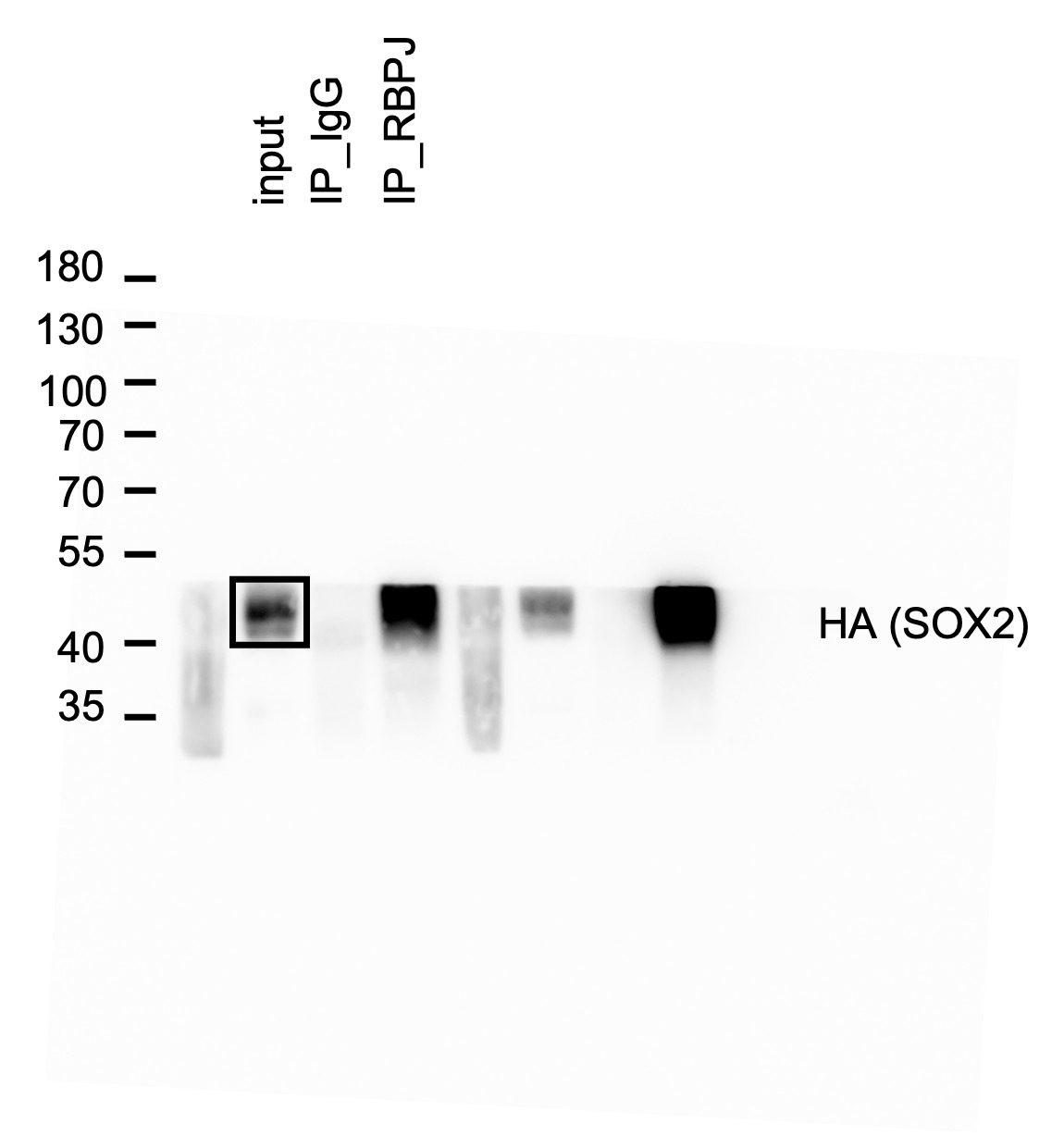

Supplement: Supplementary file 11 — Source data Fig. 3 [file 44321_2024_161_MOESM11_ESM.zip › Fig3/3E/IP_RBPJ MB157iSOX2 HA(SOX2).jpg]

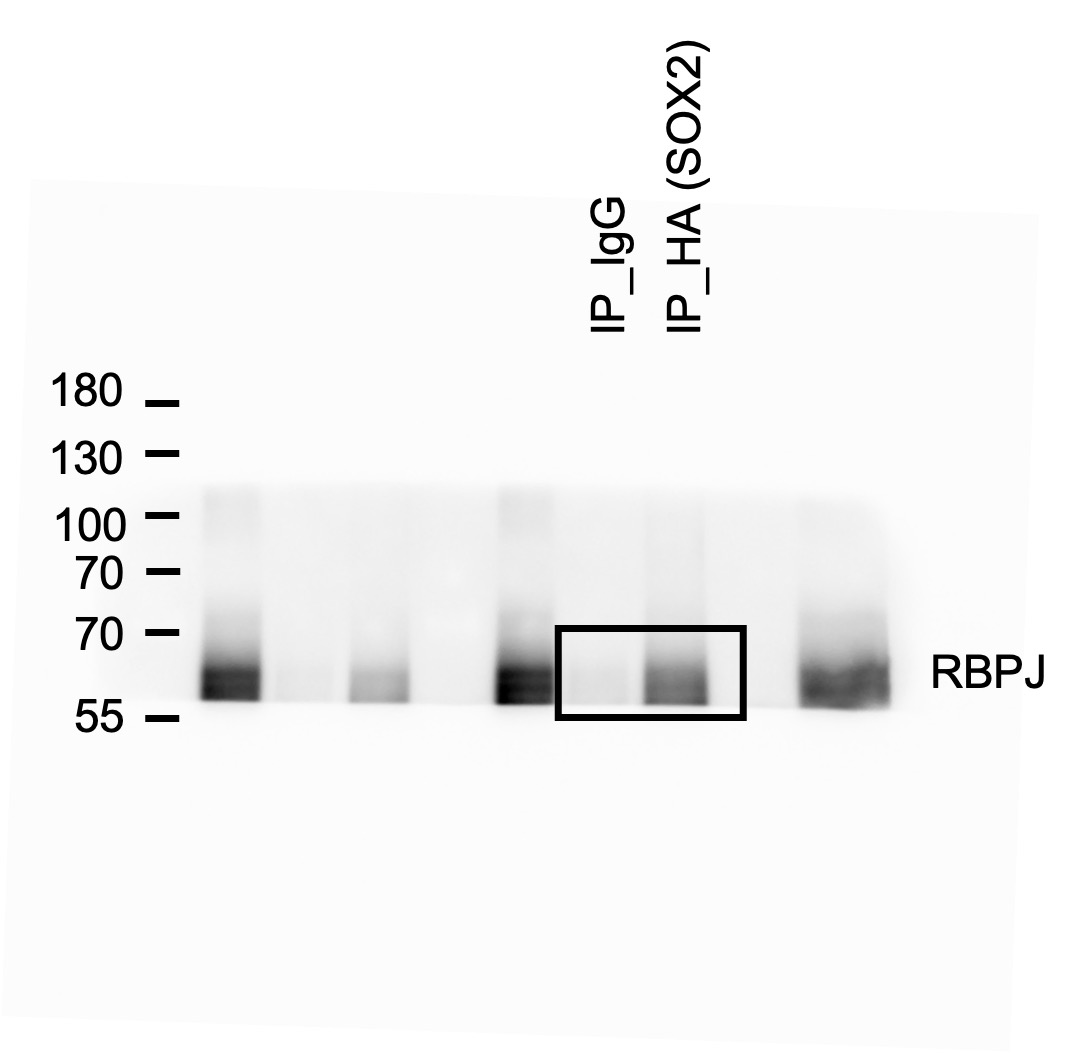

Supplement: Supplementary file 11 — Source data Fig. 3 [file 44321_2024_161_MOESM11_ESM.zip › Fig3/3E/IP_HA(SOX2) MB157iSOX2 RBPJ.jpg]

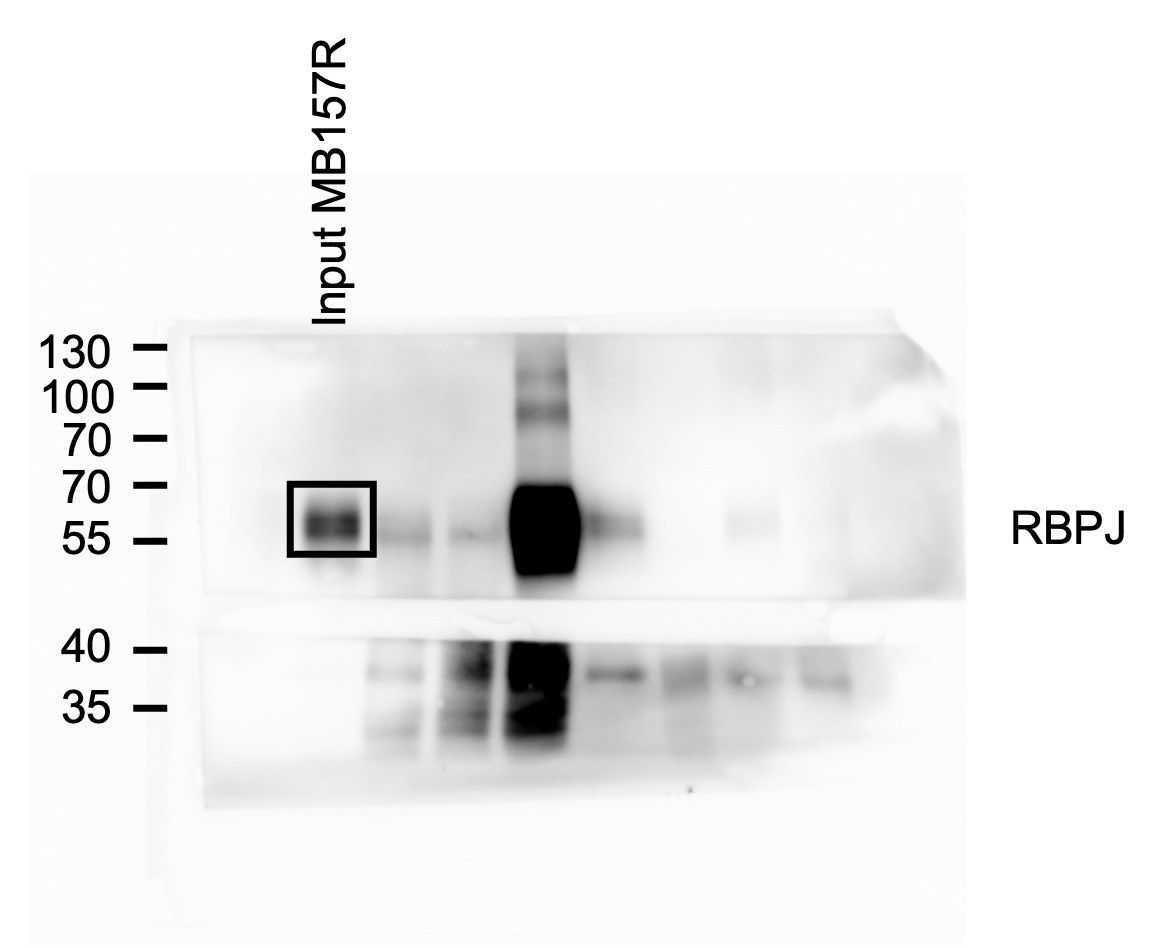

Supplement: Supplementary file 11 — Source data Fig. 3 [file 44321_2024_161_MOESM11_ESM.zip › Fig3/3E/IP_input MB157R RBPJ.jpg]

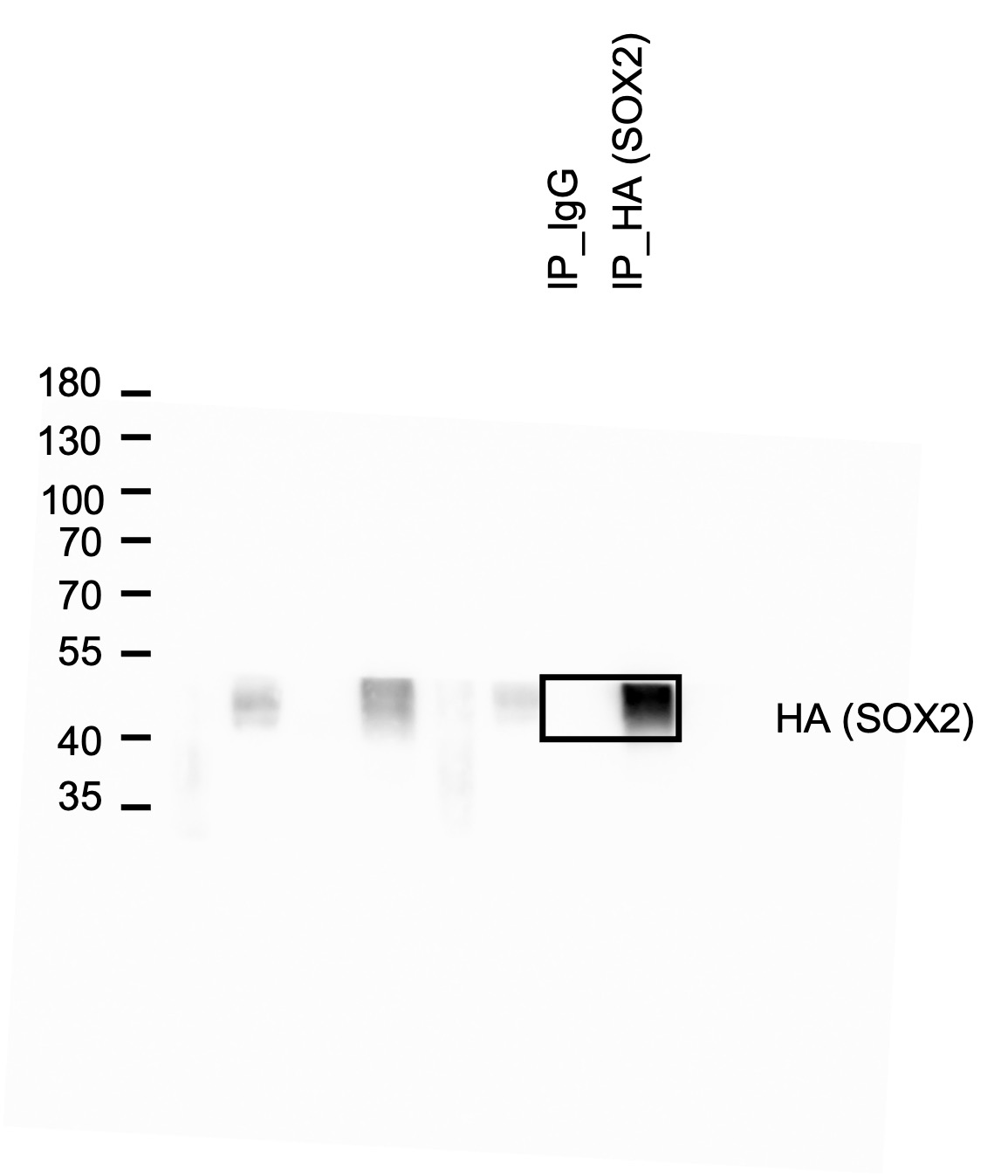

Supplement: Supplementary file 11 — Source data Fig. 3 [file 44321_2024_161_MOESM11_ESM.zip › Fig3/3E/IP_HA(SOX2) MB157iSOX2 HA(SOX2).jpg]

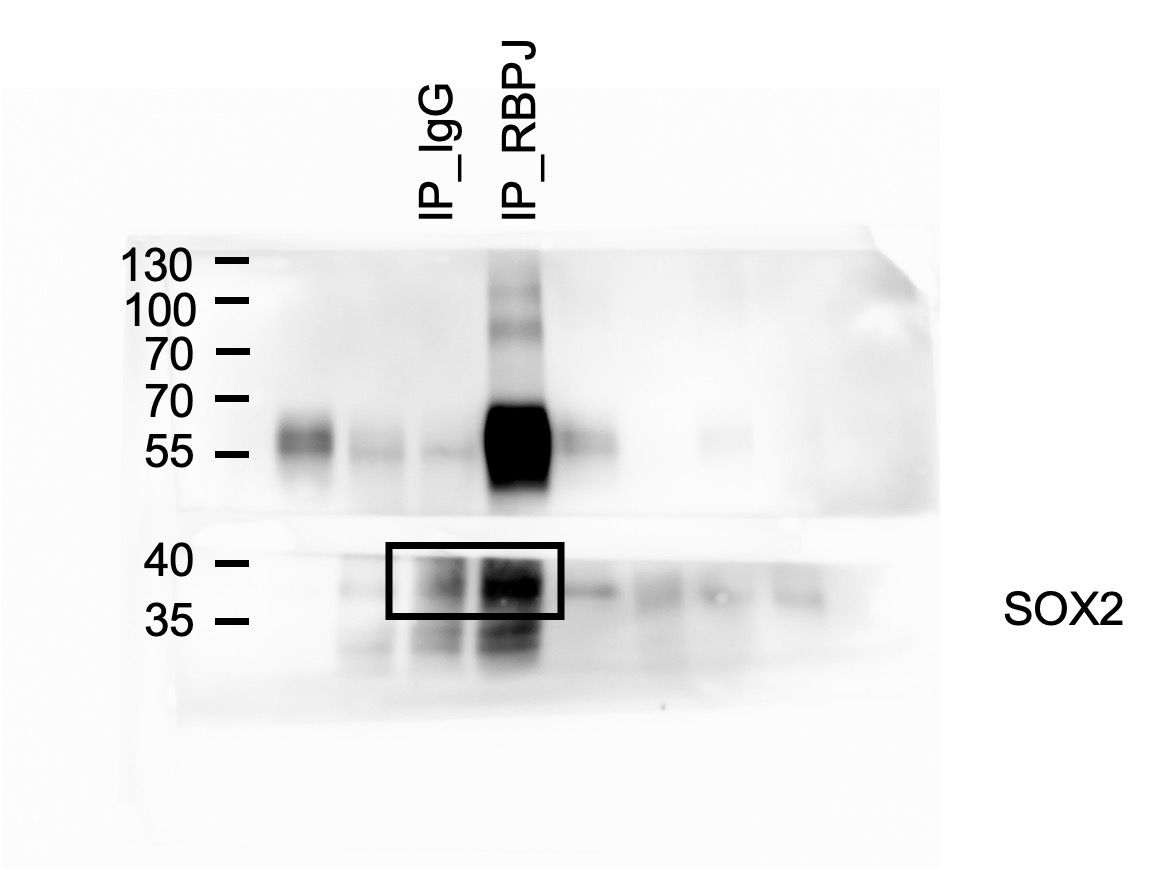

Supplement: Supplementary file 11 — Source data Fig. 3 [file 44321_2024_161_MOESM11_ESM.zip › Fig3/3E/IP_RBPJ MB157R SOX2.jpg]

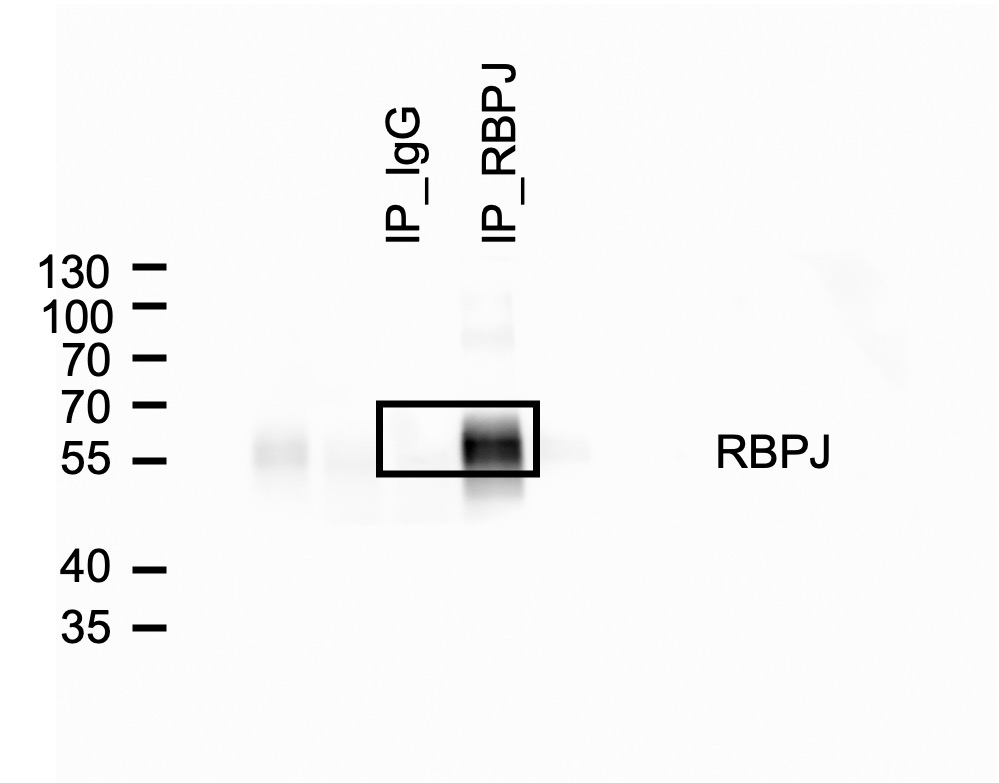

Supplement: Supplementary file 11 — Source data Fig. 3 [file 44321_2024_161_MOESM11_ESM.zip › Fig3/3E/IP_RBPJ MB157R RBPJ.jpg]

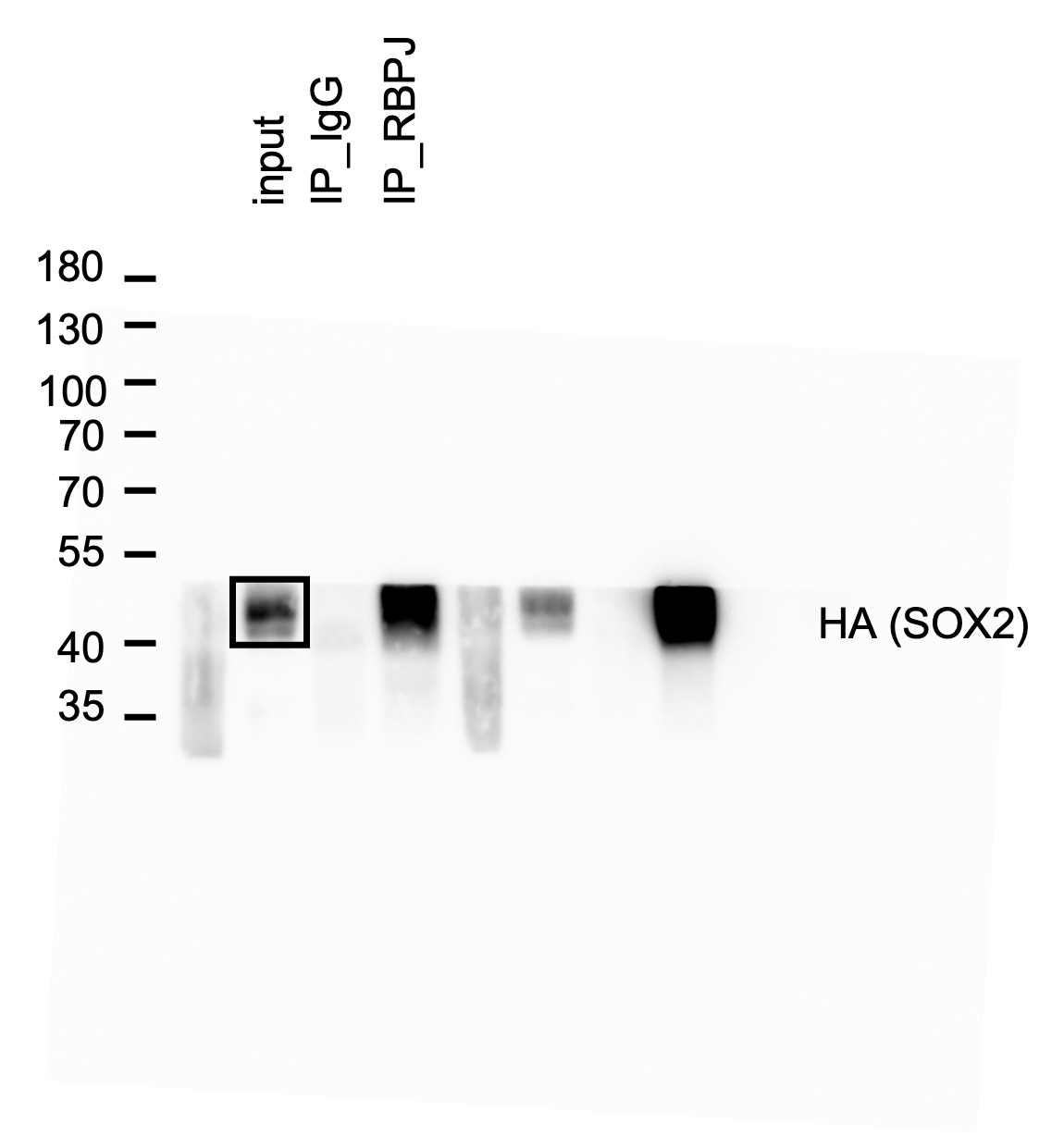

Supplement: Supplementary file 11 — Source data Fig. 3 [file 44321_2024_161_MOESM11_ESM.zip › Fig3/3E/IP_input MB157iSOX2 HA(SOX2).jpg]

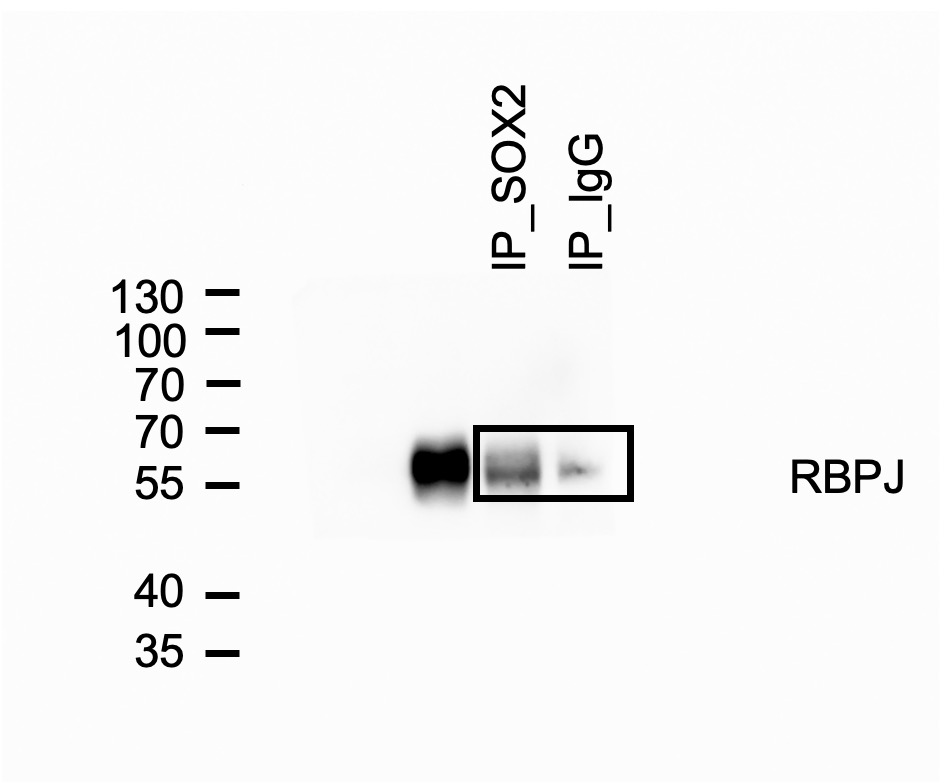

Supplement: Supplementary file 11 — Source data Fig. 3 [file 44321_2024_161_MOESM11_ESM.zip › Fig3/3E/IP_SOX2 MB157R RBPJ.jpg]

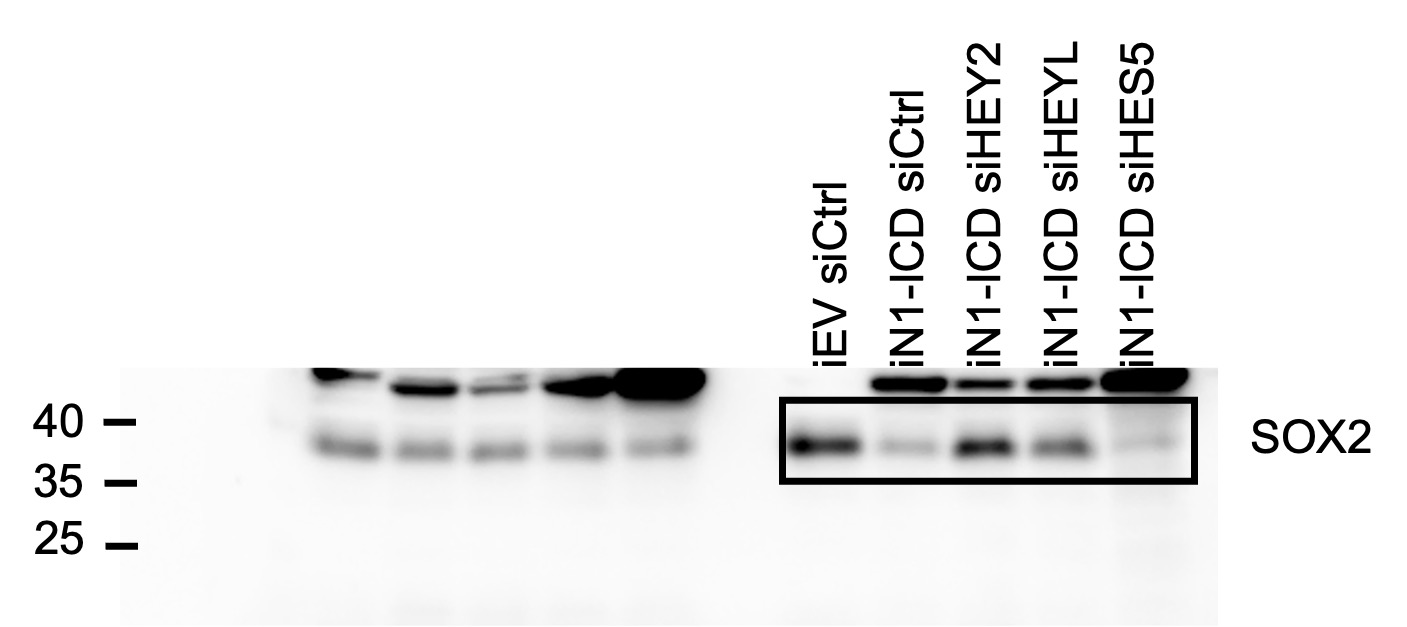

Supplement: Supplementary file 12 — Source data Fig. 4 [file 44321_2024_161_MOESM12_ESM.zip › Fig4/4E/Western MB157R iNICD siHEY SOX2.jpg]

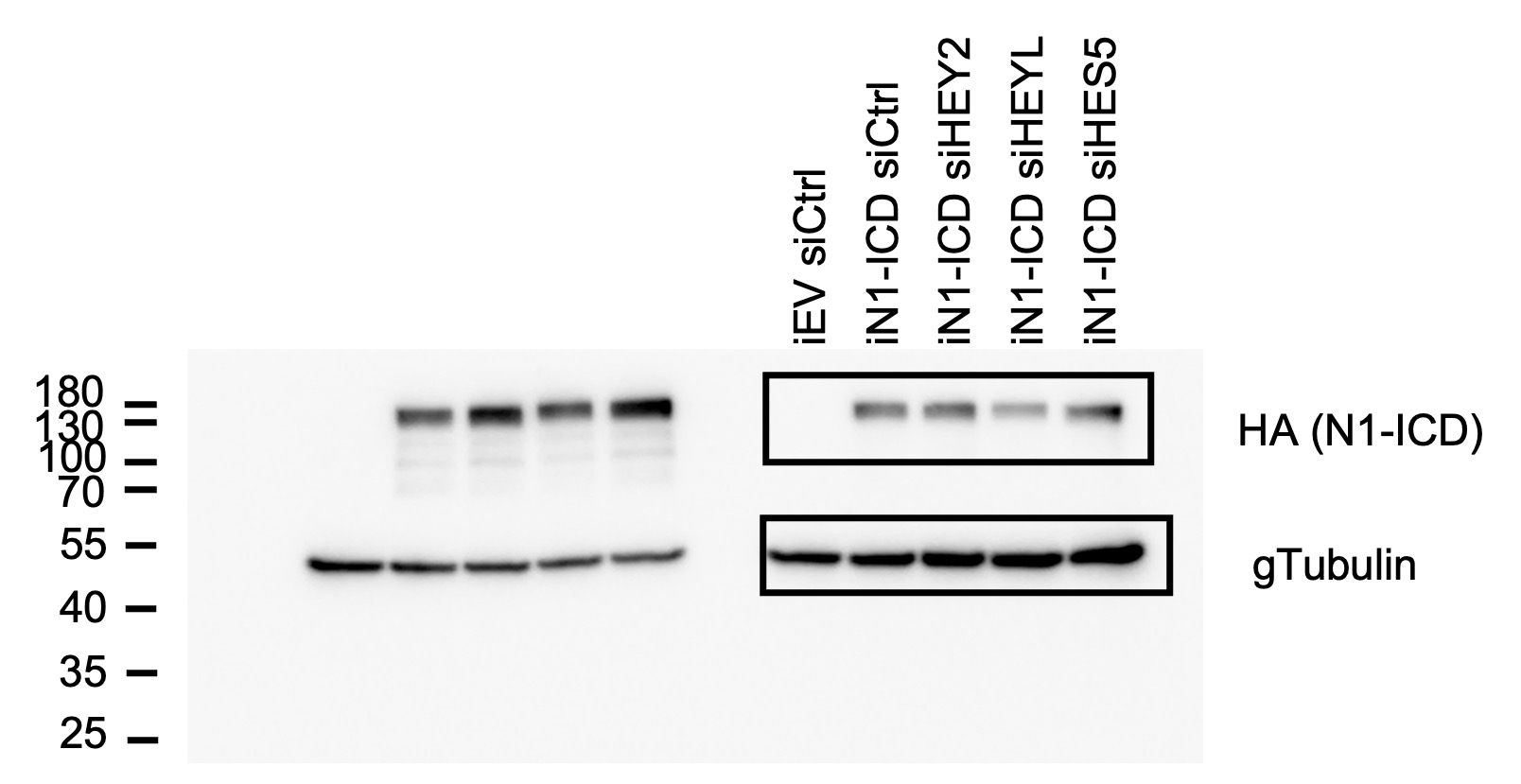

Supplement: Supplementary file 12 — Source data Fig. 4 [file 44321_2024_161_MOESM12_ESM.zip › Fig4/4E/Western MB157R iNICD siHEY HA gTubulin.jpg]

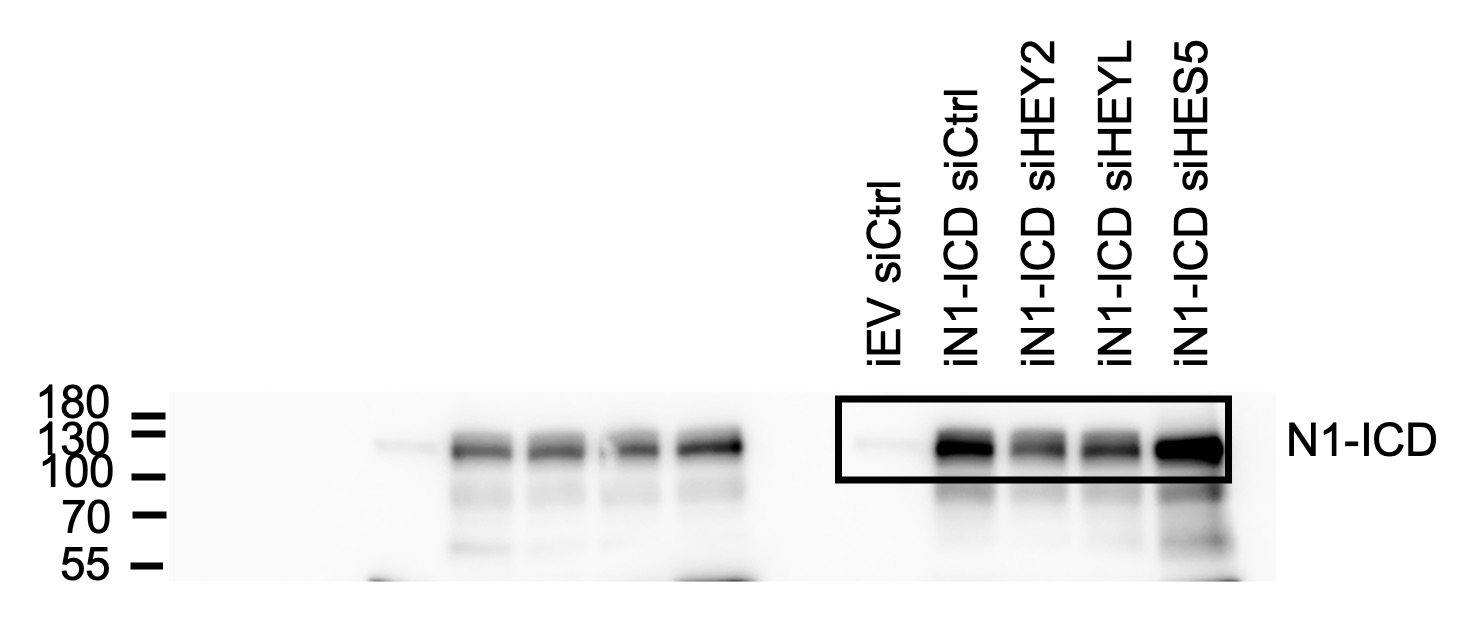

Supplement: Supplementary file 12 — Source data Fig. 4 [file 44321_2024_161_MOESM12_ESM.zip › Fig4/4E/Western MB157R iNICD siHEY N1-ICD.jpg]

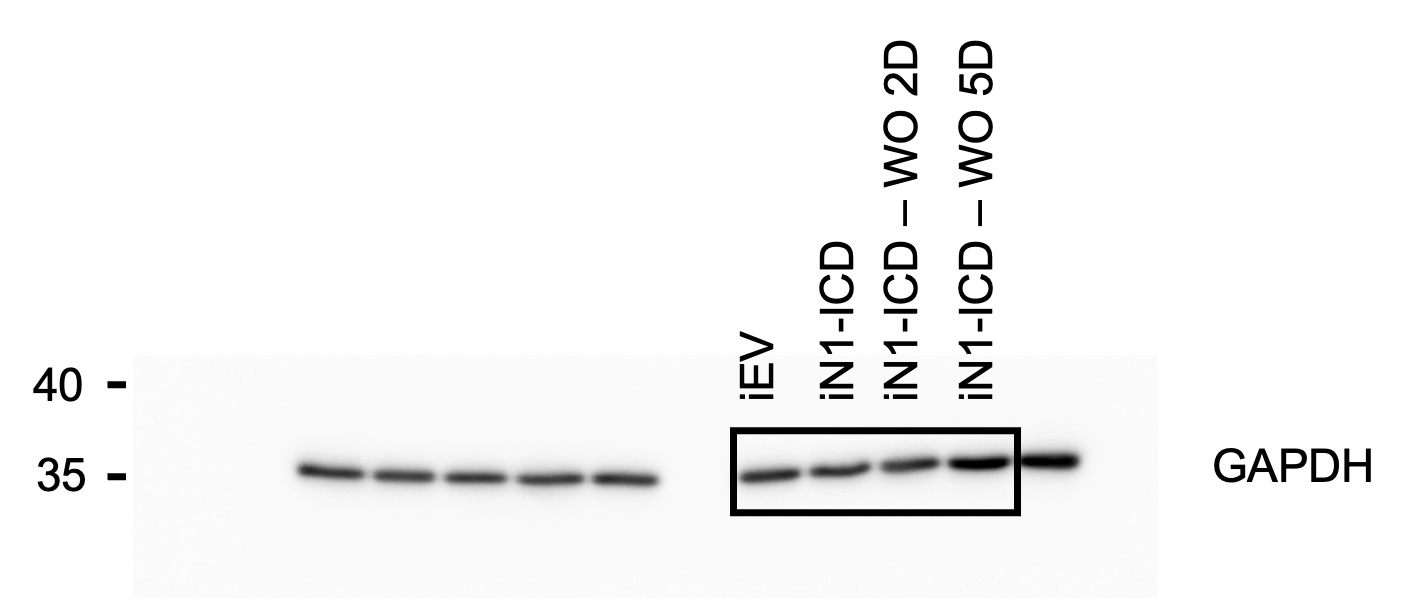

Supplement: Supplementary file 12 — Source data Fig. 4 [file 44321_2024_161_MOESM12_ESM.zip › Fig4/4A/Western MB157R iNICD GAPDH.jpg]

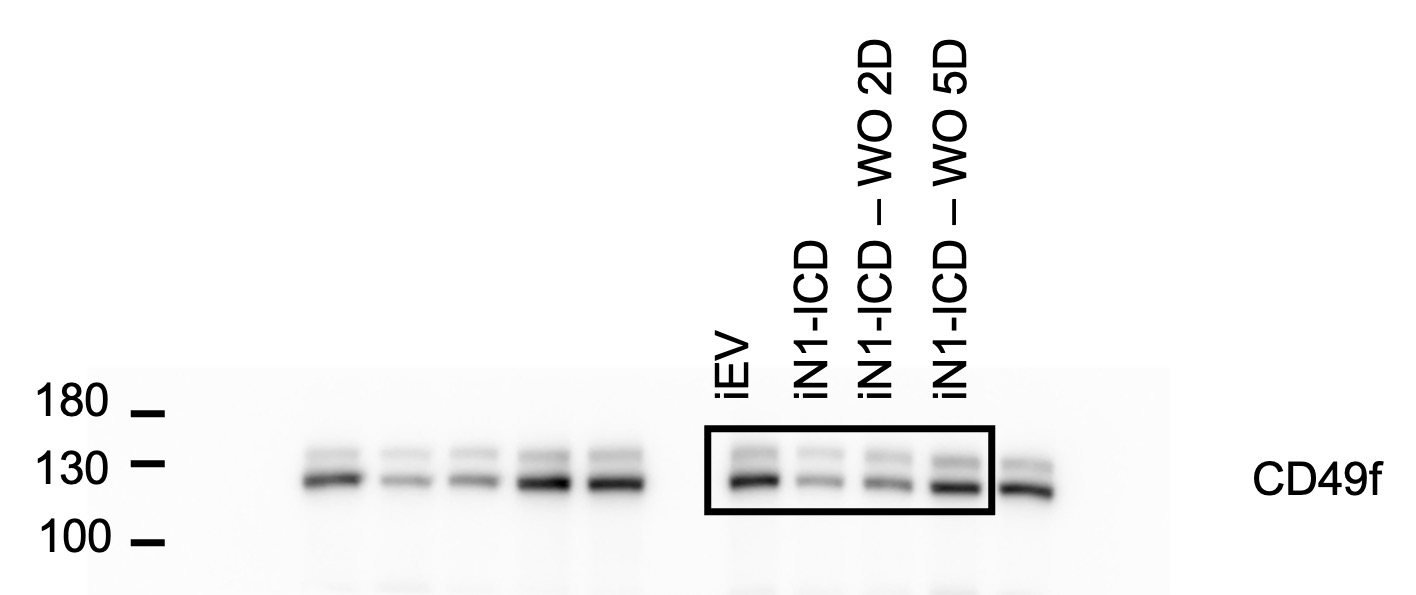

Supplement: Supplementary file 12 — Source data Fig. 4 [file 44321_2024_161_MOESM12_ESM.zip › Fig4/4A/Western MB157R iNICD CD49f.jpg]

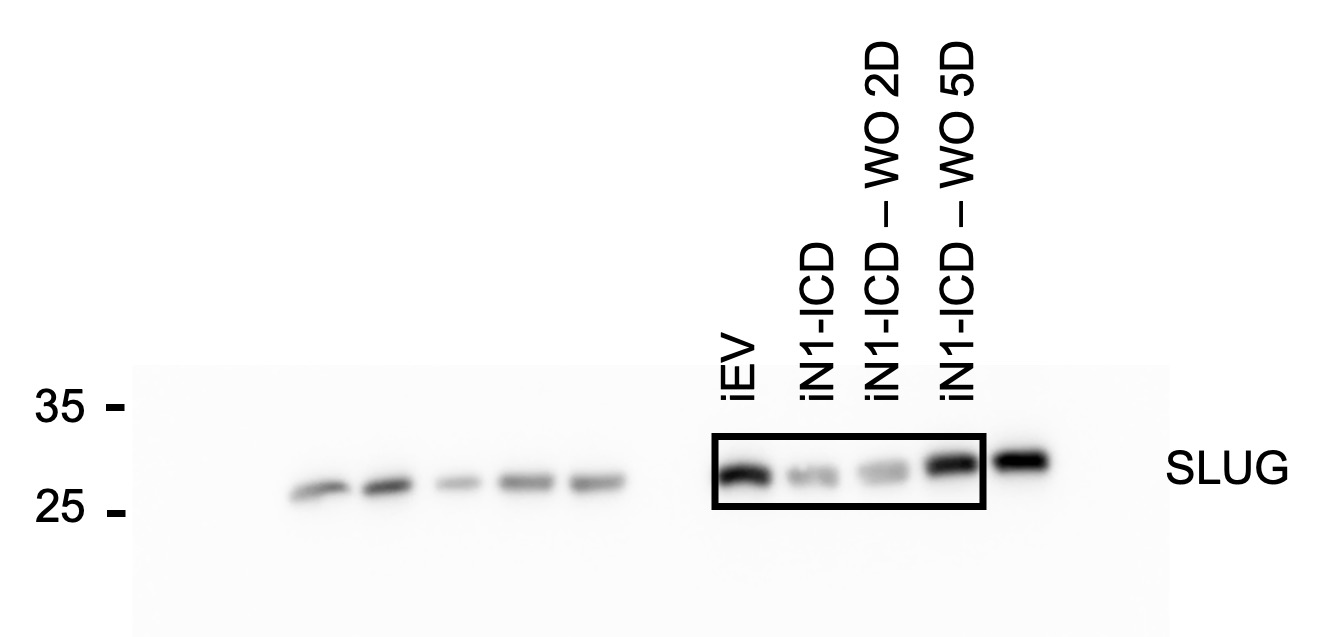

Supplement: Supplementary file 12 — Source data Fig. 4 [file 44321_2024_161_MOESM12_ESM.zip › Fig4/4A/Western MB157R iNICD SLUG.jpg]

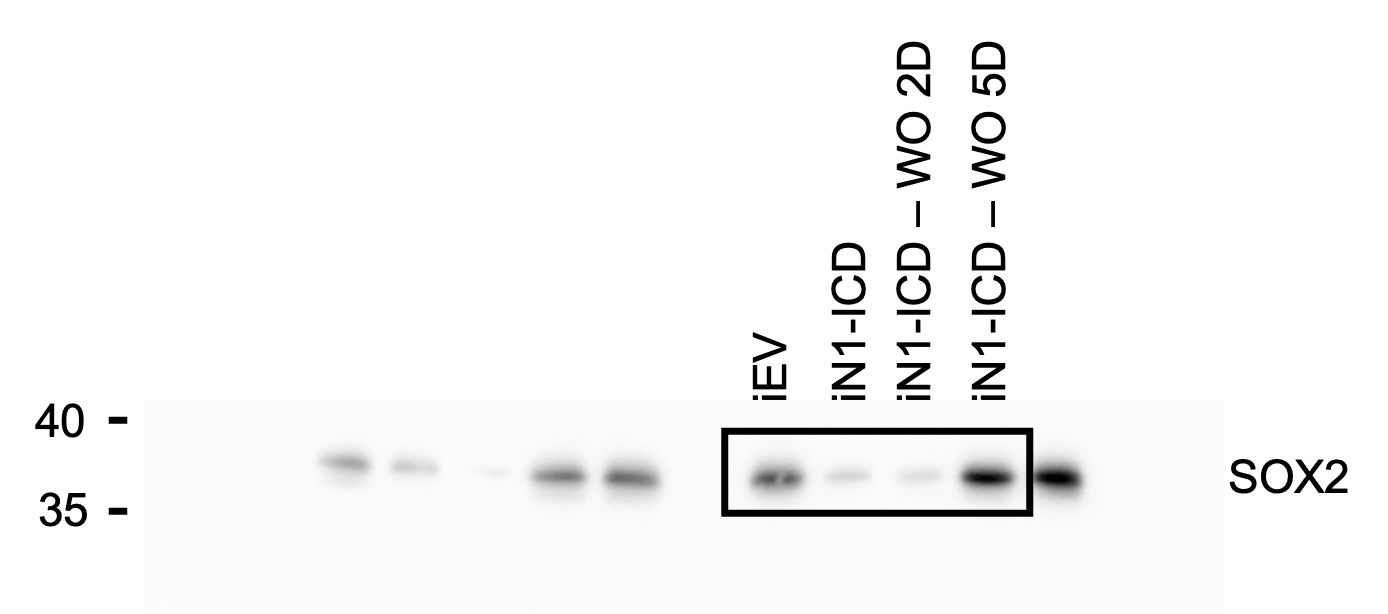

Supplement: Supplementary file 12 — Source data Fig. 4 [file 44321_2024_161_MOESM12_ESM.zip › Fig4/4A/Western MB157R iNICD SOX2.jpg]

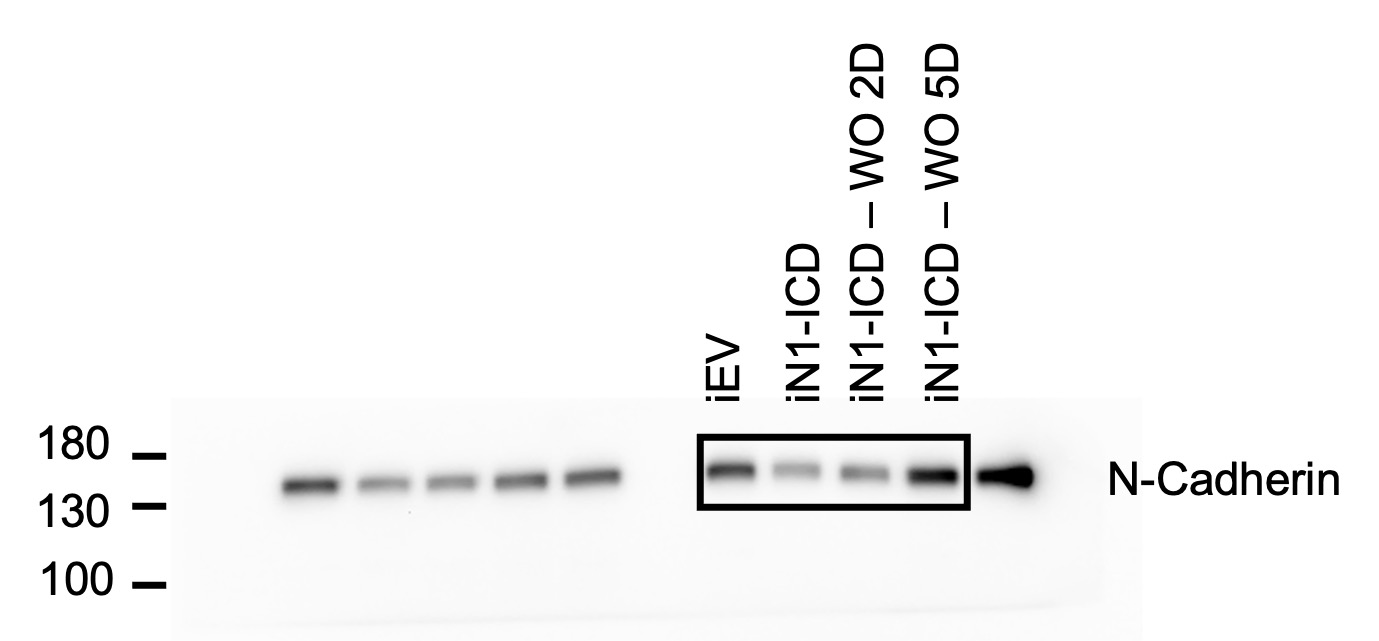

Supplement: Supplementary file 12 — Source data Fig. 4 [file 44321_2024_161_MOESM12_ESM.zip › Fig4/4A/Western MB157R iNICD N-cadherin.jpg]

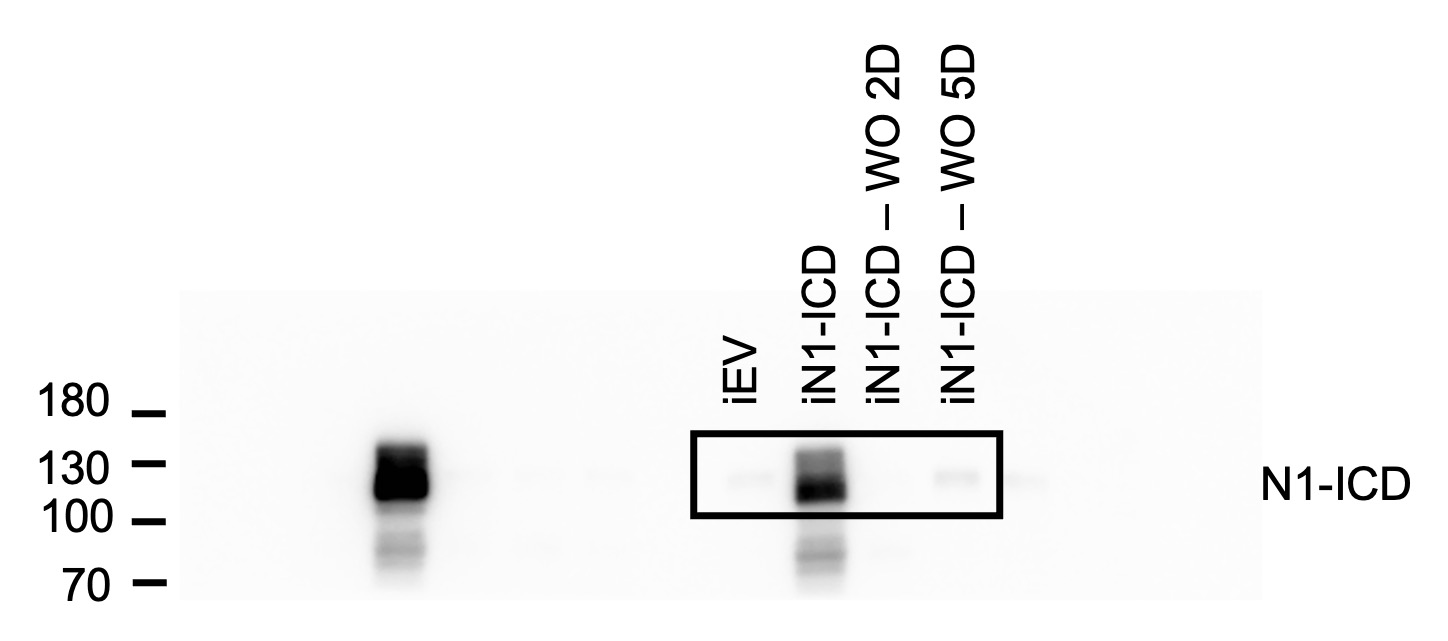

Supplement: Supplementary file 12 — Source data Fig. 4 [file 44321_2024_161_MOESM12_ESM.zip › Fig4/4A/Western MB157R iNICD N1-ICD.jpg]

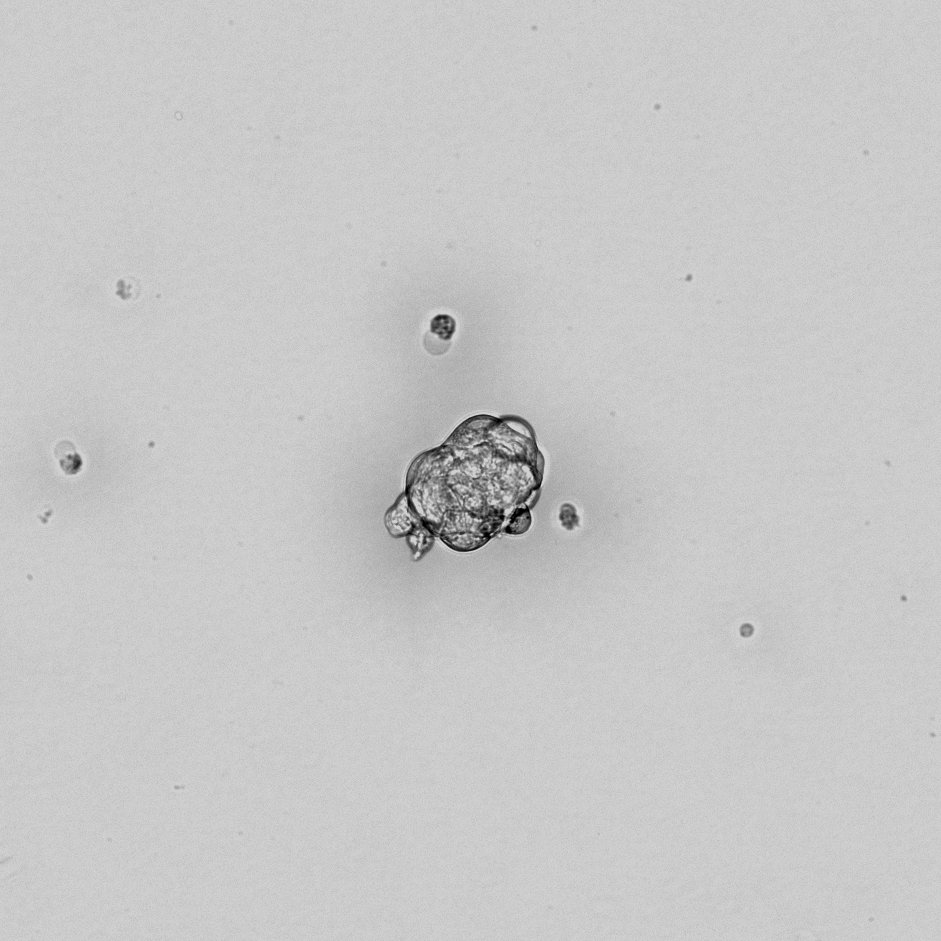

Supplement: Supplementary file 12 — Source data Fig. 4 [file 44321_2024_161_MOESM12_ESM.zip › Fig4/4C/Tumorsphere MB157R iNICD.jpg]

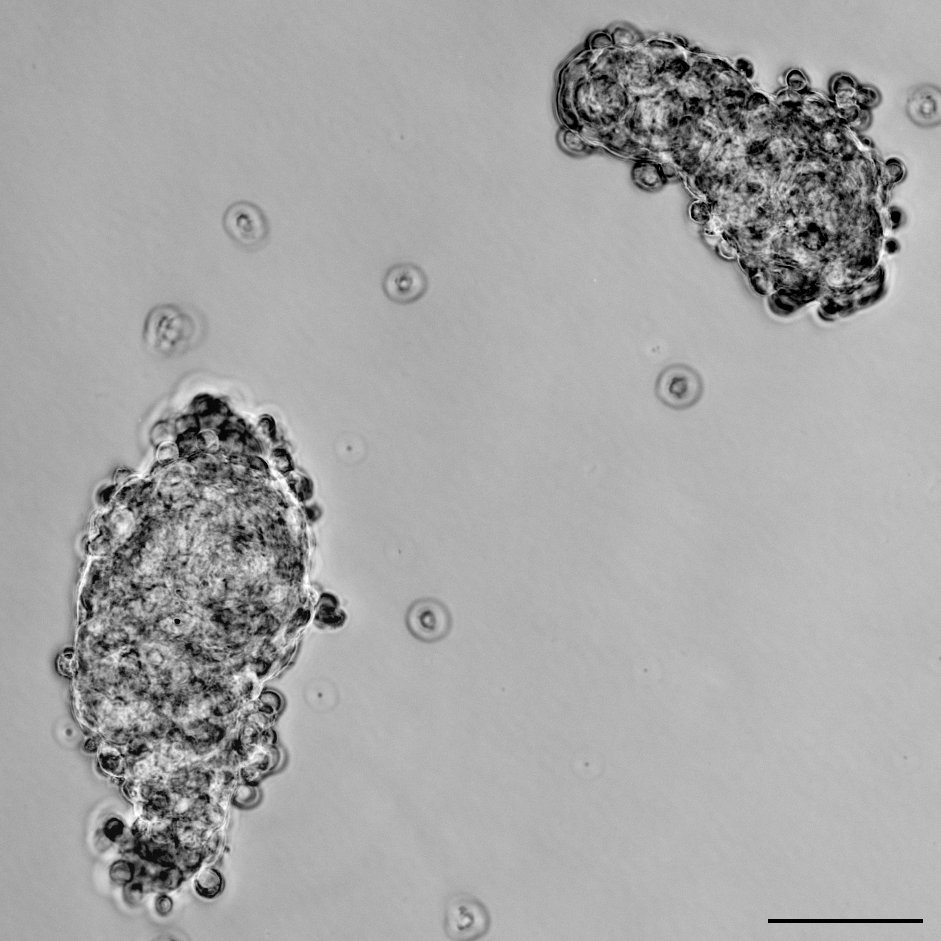

Supplement: Supplementary file 12 — Source data Fig. 4 [file 44321_2024_161_MOESM12_ESM.zip › Fig4/4C/Tumorsphere MB157R iEV.jpg]

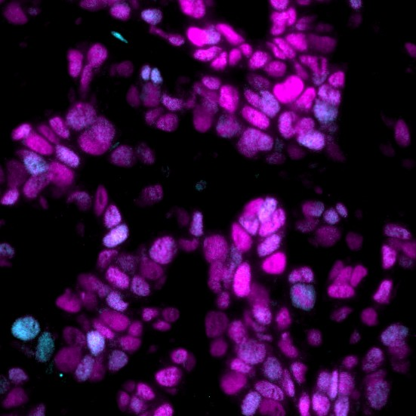

Supplement: Supplementary file 13 — Source data Fig. 5 [file 44321_2024_161_MOESM13_ESM.zip › Fig5/5C/IF_HCC1806_NICD_SOX2_1.tiff]

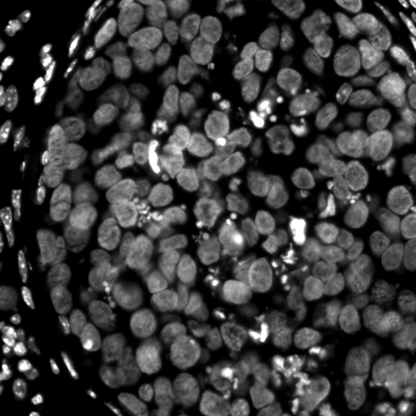

Supplement: Supplementary file 13 — Source data Fig. 5 [file 44321_2024_161_MOESM13_ESM.zip › Fig5/5C/IF_HCC1599_DAPI.tiff]

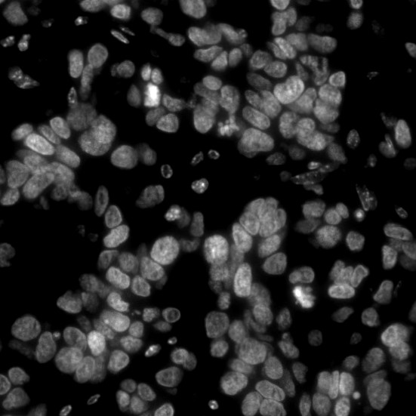

Supplement: Supplementary file 13 — Source data Fig. 5 [file 44321_2024_161_MOESM13_ESM.zip › Fig5/5C/IF_HCC1806_DAPI.tiff]

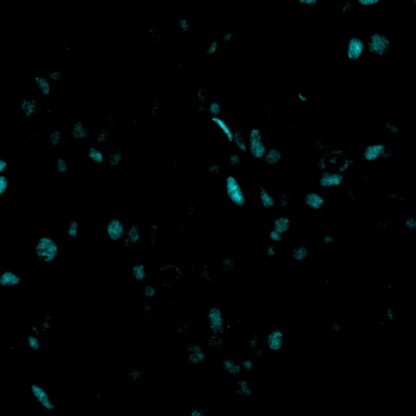

Supplement: Supplementary file 13 — Source data Fig. 5 [file 44321_2024_161_MOESM13_ESM.zip › Fig5/5C/IF_MB157R_NICD.tiff]

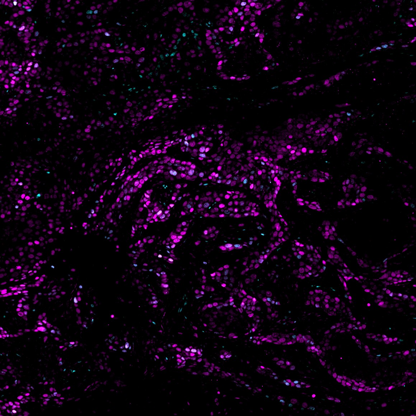

Supplement: Supplementary file 13 — Source data Fig. 5 [file 44321_2024_161_MOESM13_ESM.zip › Fig5/5C/IF_MB157R_NICD_SOX2_2.tiff]

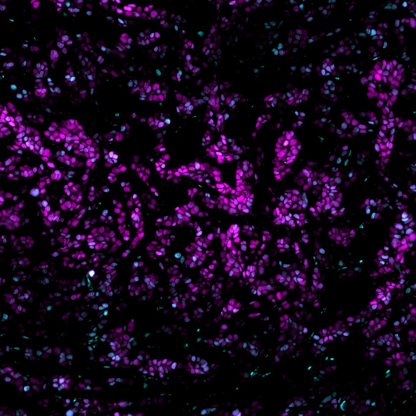

Supplement: Supplementary file 13 — Source data Fig. 5 [file 44321_2024_161_MOESM13_ESM.zip › Fig5/5C/IF_HCC1806_NICD_SOX2_2.tiff]

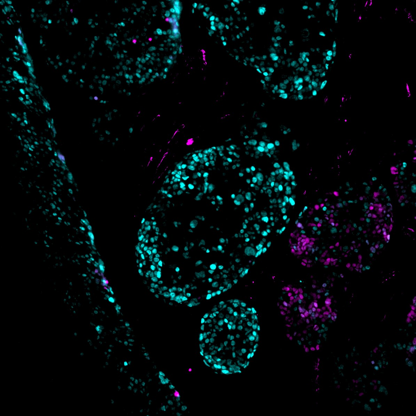

Supplement: Supplementary file 13 — Source data Fig. 5 [file 44321_2024_161_MOESM13_ESM.zip › Fig5/5C/IF_MB157_NICD_SOX2_1.tiff]

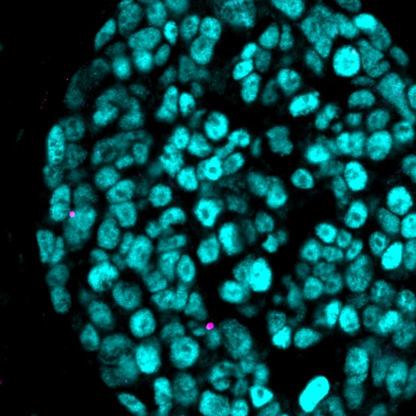

Supplement: Supplementary file 13 — Source data Fig. 5 [file 44321_2024_161_MOESM13_ESM.zip › Fig5/5C/IF_HCC1599_NICD_SOX2_1.tiff]

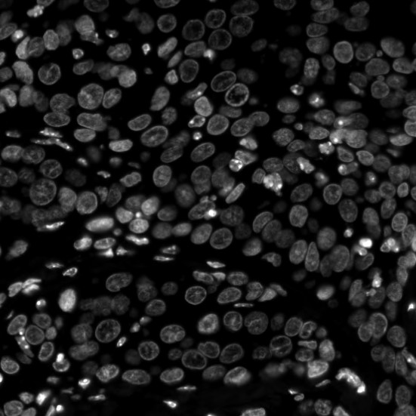

Supplement: Supplementary file 13 — Source data Fig. 5 [file 44321_2024_161_MOESM13_ESM.zip › Fig5/5C/IF_MB157R_DAPI.tiff]

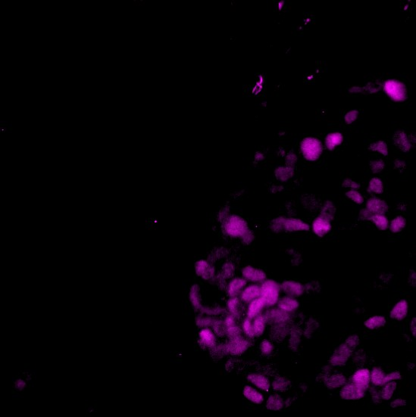

Supplement: Supplementary file 13 — Source data Fig. 5 [file 44321_2024_161_MOESM13_ESM.zip › Fig5/5C/IF_MB157_SOX2.tiff]

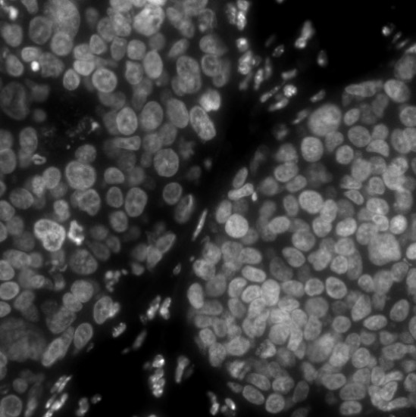

Supplement: Supplementary file 13 — Source data Fig. 5 [file 44321_2024_161_MOESM13_ESM.zip › Fig5/5C/IF_MB157_DAPI.tiff]

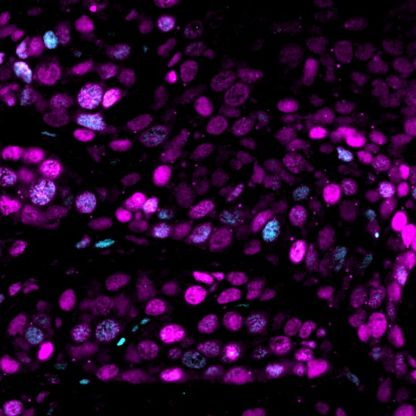

Supplement: Supplementary file 13 — Source data Fig. 5 [file 44321_2024_161_MOESM13_ESM.zip › Fig5/5C/IF_MB157R_NICD_SOX2_1.tiff]

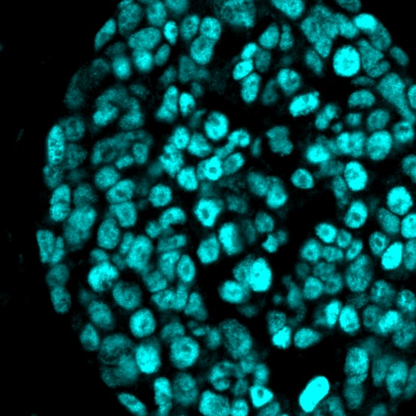

Supplement: Supplementary file 13 — Source data Fig. 5 [file 44321_2024_161_MOESM13_ESM.zip › Fig5/5C/IF_HCC1599_NICD.tiff]

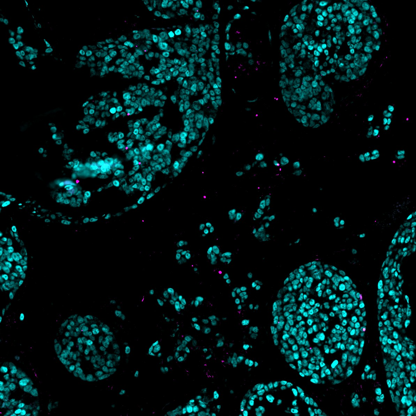

Supplement: Supplementary file 13 — Source data Fig. 5 [file 44321_2024_161_MOESM13_ESM.zip › Fig5/5C/IF_HCC1599_NICD_SOX2_2.tiff]

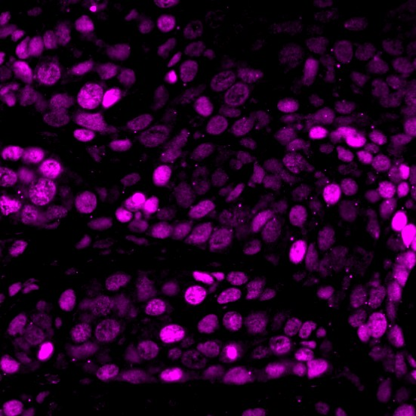

Supplement: Supplementary file 13 — Source data Fig. 5 [file 44321_2024_161_MOESM13_ESM.zip › Fig5/5C/IF_MB157R_SOX2.tiff]

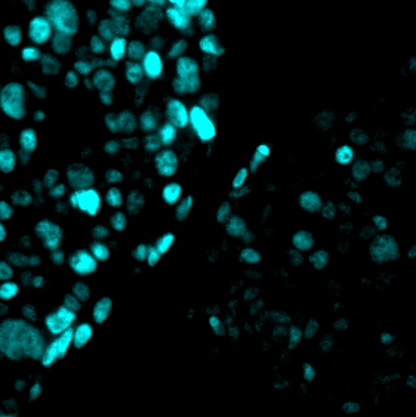

Supplement: Supplementary file 13 — Source data Fig. 5 [file 44321_2024_161_MOESM13_ESM.zip › Fig5/5C/IF_MB157_NICD.tiff]

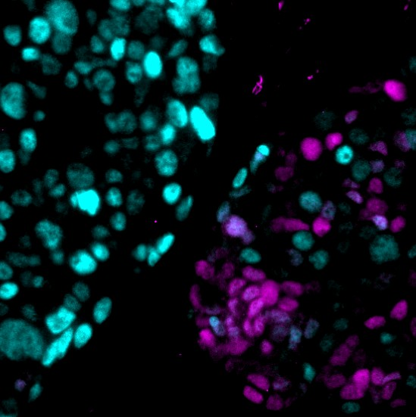

Supplement: Supplementary file 13 — Source data Fig. 5 [file 44321_2024_161_MOESM13_ESM.zip › Fig5/5C/IF_MB157_NICD_SOX2_2.tiff]

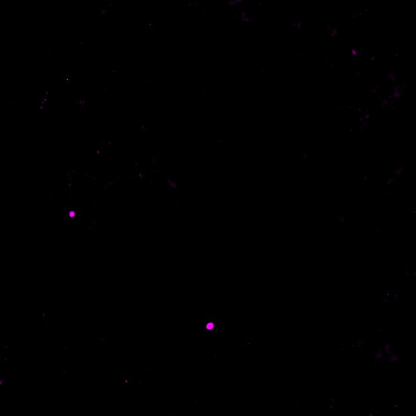

Supplement: Supplementary file 13 — Source data Fig. 5 [file 44321_2024_161_MOESM13_ESM.zip › Fig5/5C/IF_HCC1599_SOX2.tiff]

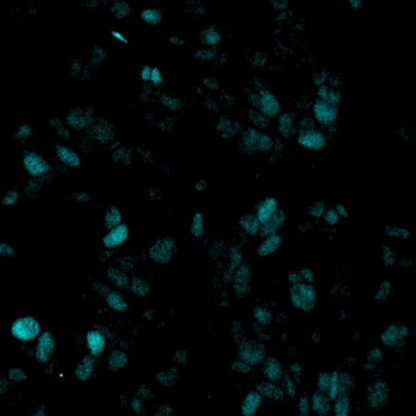

Supplement: Supplementary file 13 — Source data Fig. 5 [file 44321_2024_161_MOESM13_ESM.zip › Fig5/5C/IF_HCC1806_NICD.tiff]

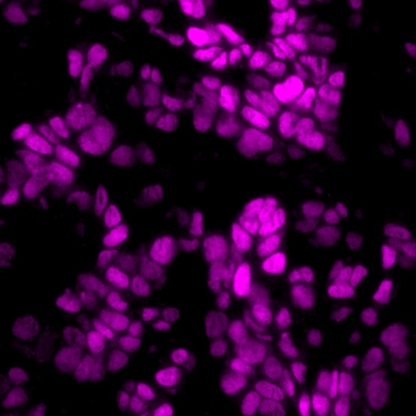

Supplement: Supplementary file 13 — Source data Fig. 5 [file 44321_2024_161_MOESM13_ESM.zip › Fig5/5C/IF_HCC1806_SOX2.tiff]

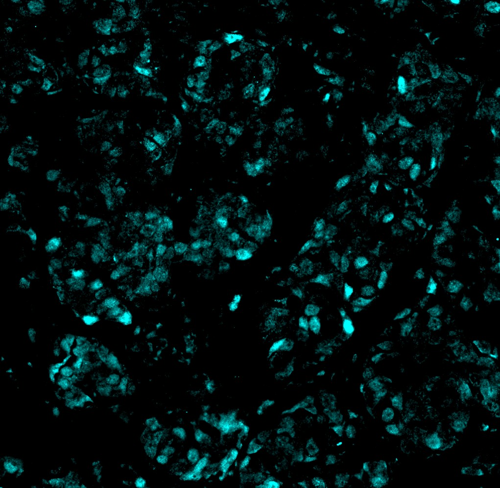

Supplement: Supplementary file 13 — Source data Fig. 5 [file 44321_2024_161_MOESM13_ESM.zip › Fig5/5E/Br931 TMA_2 IF NICD.tiff]

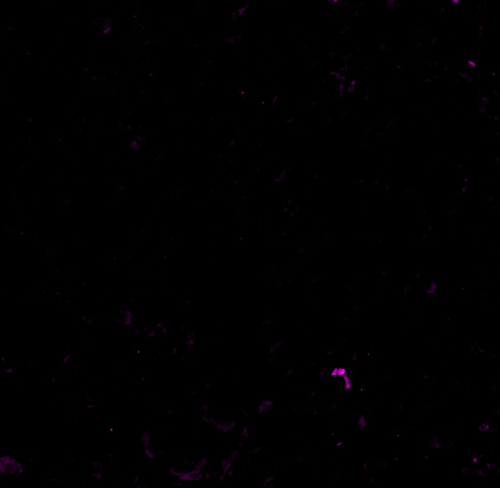

Supplement: Supplementary file 13 — Source data Fig. 5 [file 44321_2024_161_MOESM13_ESM.zip › Fig5/5E/Br931 TMA_2 IF SOX2.tiff]

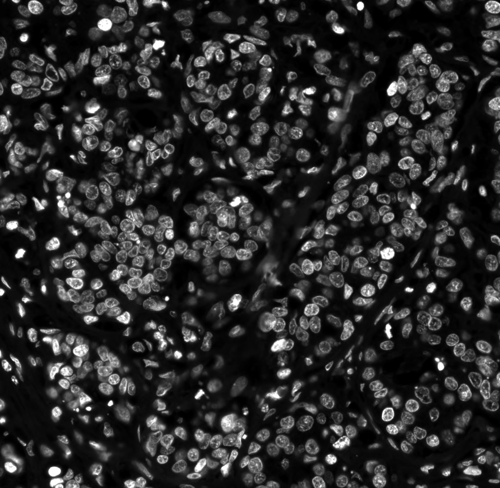

Supplement: Supplementary file 13 — Source data Fig. 5 [file 44321_2024_161_MOESM13_ESM.zip › Fig5/5E/Br931 TMA_2 IF DAPI.tiff]

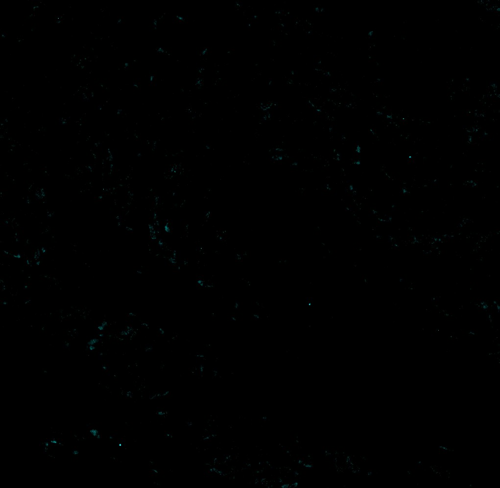

Supplement: Supplementary file 13 — Source data Fig. 5 [file 44321_2024_161_MOESM13_ESM.zip › Fig5/5E/Br931 TMA_1 IF NICD.tiff]

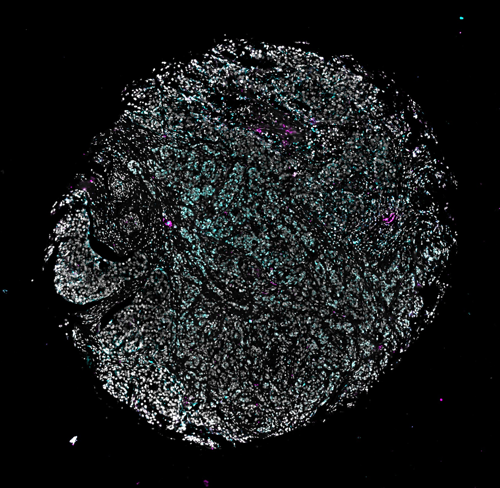

Supplement: Supplementary file 13 — Source data Fig. 5 [file 44321_2024_161_MOESM13_ESM.zip › Fig5/5E/Br931 TMA_2 IF NICD SOX2 DAPI.tiff]

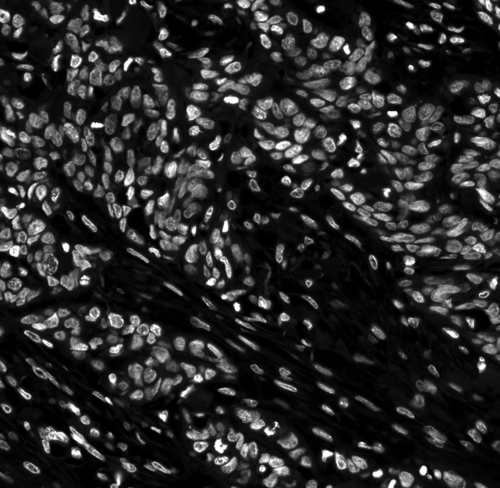

Supplement: Supplementary file 13 — Source data Fig. 5 [file 44321_2024_161_MOESM13_ESM.zip › Fig5/5E/Br931 TMA_1 IF DAPI.tiff]

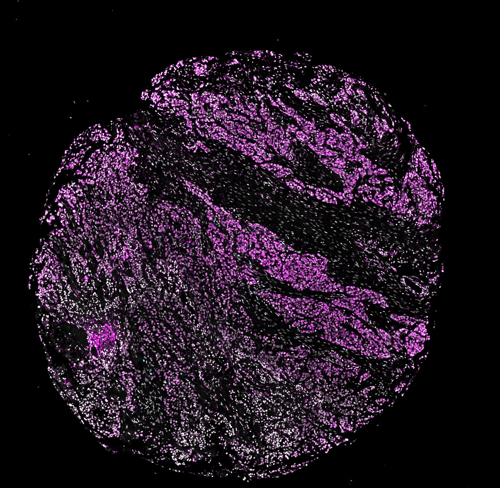

Supplement: Supplementary file 13 — Source data Fig. 5 [file 44321_2024_161_MOESM13_ESM.zip › Fig5/5E/Br931 TMA_1 IF NICD SOX2 DAPI.tiff]

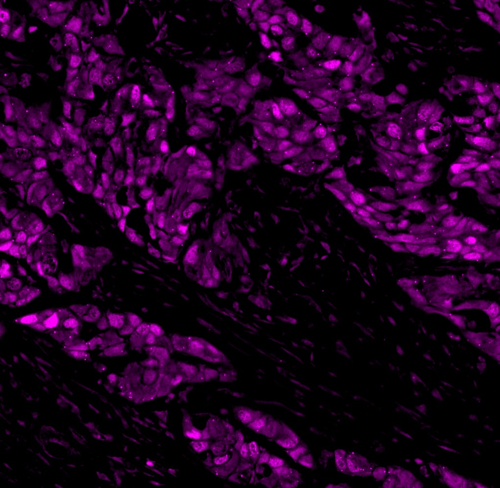

Supplement: Supplementary file 13 — Source data Fig. 5 [file 44321_2024_161_MOESM13_ESM.zip › Fig5/5E/Br931 TMA_1 IF SOX2.tiff]

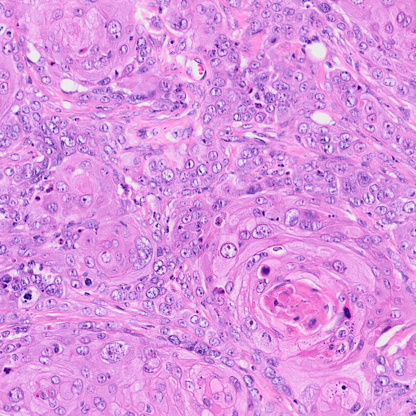

Supplement: Supplementary file 13 — Source data Fig. 5 [file 44321_2024_161_MOESM13_ESM.zip › Fig5/5B/HE_MB157R_2.tiff]

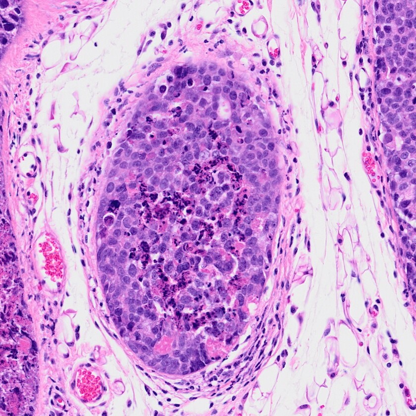

Supplement: Supplementary file 13 — Source data Fig. 5 [file 44321_2024_161_MOESM13_ESM.zip › Fig5/5B/HE_MB157_2.tiff]

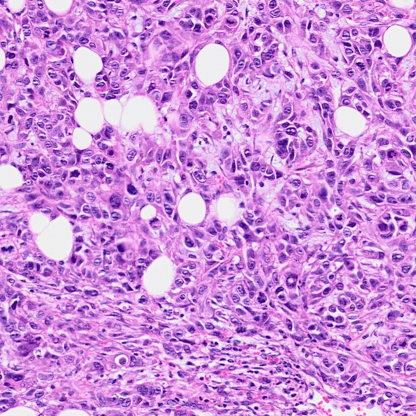

Supplement: Supplementary file 13 — Source data Fig. 5 [file 44321_2024_161_MOESM13_ESM.zip › Fig5/5B/HE_HCC1806_2.tiff]

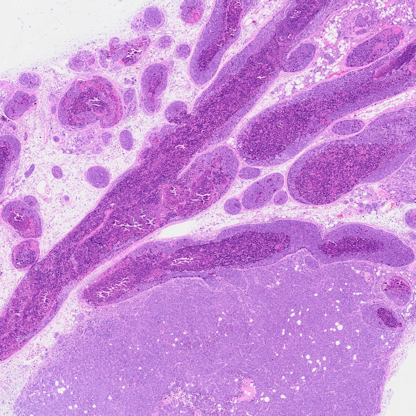

Supplement: Supplementary file 13 — Source data Fig. 5 [file 44321_2024_161_MOESM13_ESM.zip › Fig5/5B/HE_HCC1599_1.tiff]

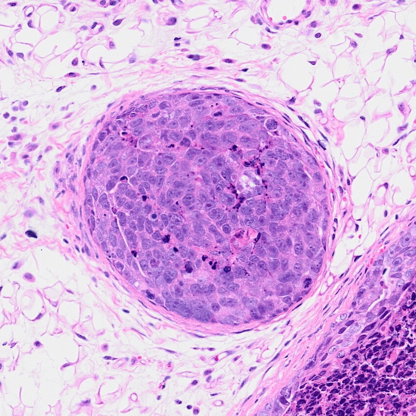

Supplement: Supplementary file 13 — Source data Fig. 5 [file 44321_2024_161_MOESM13_ESM.zip › Fig5/5B/HE_HCC1599_2.tiff]

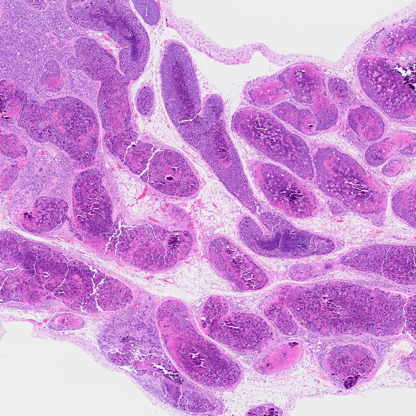

Supplement: Supplementary file 13 — Source data Fig. 5 [file 44321_2024_161_MOESM13_ESM.zip › Fig5/5B/HE_MB157_1.tiff]

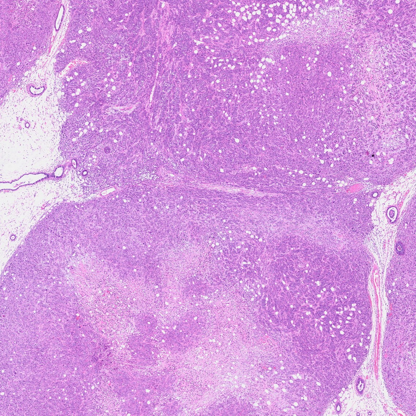

Supplement: Supplementary file 13 — Source data Fig. 5 [file 44321_2024_161_MOESM13_ESM.zip › Fig5/5B/HE_HCC1806_1.tiff]

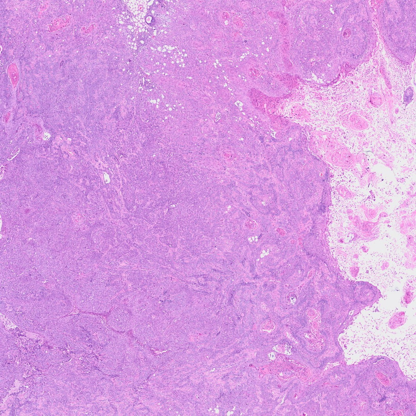

Supplement: Supplementary file 13 — Source data Fig. 5 [file 44321_2024_161_MOESM13_ESM.zip › Fig5/5B/HE_MB157R_1.tiff]
