# Supplementary material for: Reciprocal inhibition of NOTCH and SOX2 shapes tumor cell plasticity and therapeutic escape in triple-negative breast cancer
Source: EMBO Mol Med. 2024 Oct 30;16(12):9. doi: 10.1038/s44321-024-00161-8 (PMC11628624; doi:10.1038/s44321-024-00161-8)
Supplement: Supplementary file 15 — Expanded View Figures [file 44321_2024_161_MOESM15_ESM.pdf]

Expanded View Figures

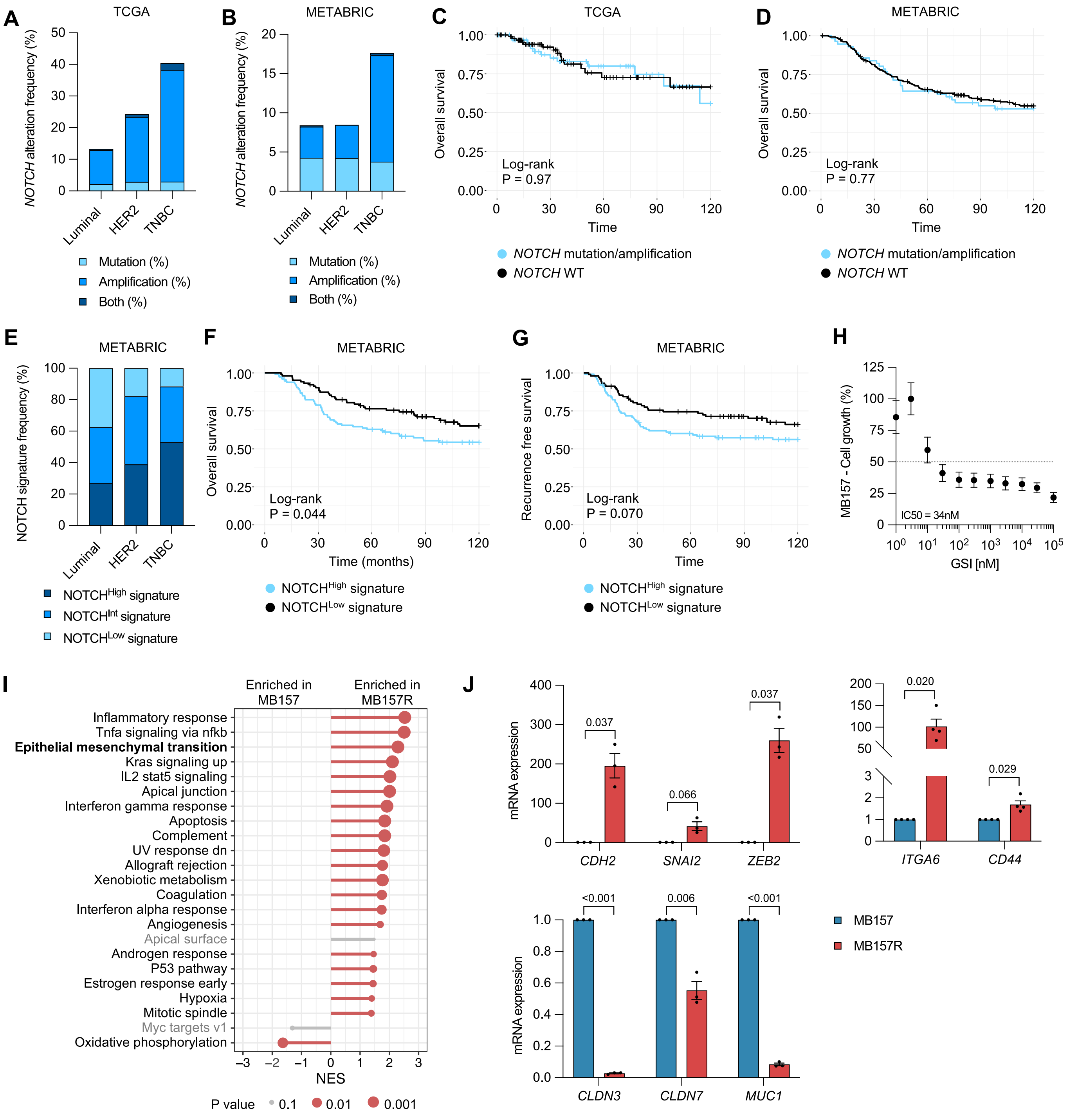

Figure EV1. Chronic exposure of NOTCH-driven TNBC cells to GSI induces drug resistance which is associated with EMT and CSC features.

(**A**) *NOTCH* mutation and amplification frequency in luminal ($n = 686$), HER2 ($n = 103$) and TNBC ($n = 168$) patients from TCGA dataset ($n = 957$). (**B**) *NOTCH* mutation and amplification frequency in luminal ($n = 1332$), HER2 ($n = 212$) and TNBC ($n = 317$) patients from METABRIC dataset ($n = 1861$). (**C**) OS of TNBC patients from TCGA dataset with *NOTCH* wild-type ($n = 100$) or altered ($n = 68$). (**D**) OS of TNBC patients from METABRIC dataset with *NOTCH* wild-type ($n = 261$) or altered ($n = 56$). (**E**) Frequency of $NOTCH^{High}$, $NOTCH^{Int}$ or $NOTCH^{Low}$ signature in luminal ($n = 1395$), HER2 ($n = 26$) and TNBC ($n = 347$) patients from METABRIC dataset ($n = 1968$). (**F**) OS of TNBC patients from METABRIC dataset with $NOTCH^{High}$ ($n = 114$) or $NOTCH^{Low}$ ($n = 103$) signature. (**G**) RFS of TNBC patients from METABRIC dataset with $NOTCH^{High}$ ($n = 114$) or $NOTCH^{Low}$ ($n = 103$) signature. (**H**) Cell growth inhibition of MB157 cells treated with GSI (1 μM) for 6 days, $n = 4$. (**I**) Hallmark GSEA from RNAseq analysis of MB157R compared to MB157 cells, $n = 3$. (**J**) Relative mRNA expression of EMT and stemness markers in MB157 and MB157R cells, $n = 3$. Data from biological replicates are represented as mean ± SEM. Log-rank test (**C**, **D**, **F**, **G**), permutation test (**I**) or Student *t* test (**J**) were used to determine *P* values.

 © The Author(s)

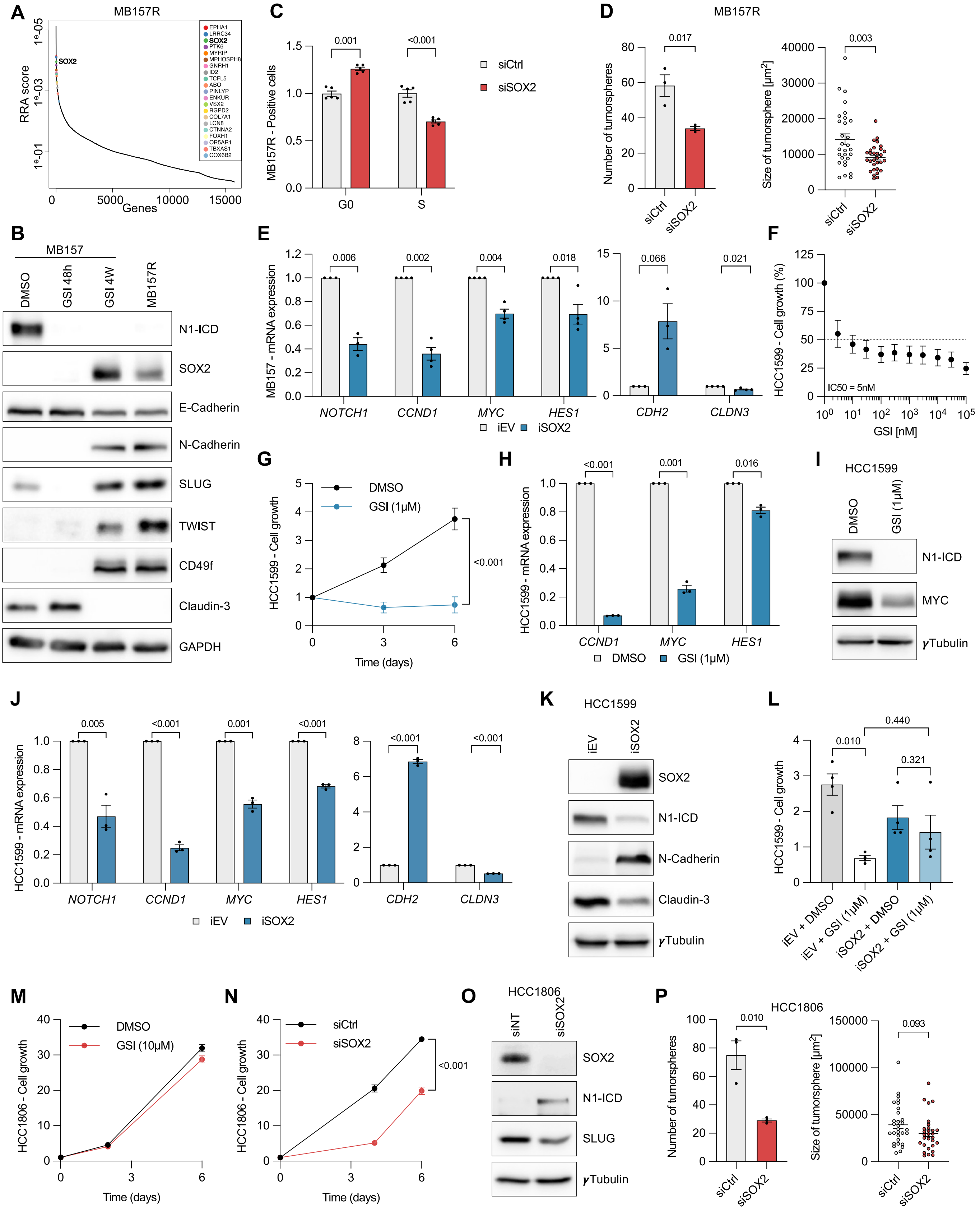
A
MB157R
RRA score
Genes
SOX2
B
MB157
DMSO
GSI 48h
GSI 4W
MB157R
N1-ICD
SOX2
E-Cadherin
N-Cadherin
SLUG
TWIST
CD49f
Claudin-3
GAPDH
C
MB157R - Positive cells
siCtrl
siSOX2
G0
S
D
MB157R
Number of tumorspheres
Size of tumorsphere [µm²]
E
MB157 - mRNA expression
NOTCH1
CCND1
MYC
HES1
CDH2
CLDN3
iEV
iSOX2
F
HCC1599 - Cell growth (%)
IC50 = 5nM
GSI [nM]
G
HCC1599 - Cell growth
DMSO
GSI (1µM)
Time (days)
H
HCC1599 - mRNA expression
I
HCC1599
MYC
γTubulin
J
HCC1599 - mRNA expression
K
HCC1599
L
HCC1599 - Cell growth
iEV + DMSO
iEV + GSI (1µM)
iSOX2 + DMSO
iSOX2 + GSI (1µM)
M
HCC1806 - Cell growth
GSI (10µM)
N
siCtrl
siSOX2
O
HCC1806
siNT
P
HCC1806

Figure EV2. SOX2 mediates resistance to GSI in TNBC inhibiting Notch signaling, promoting EMT and CSC features.

(**A**) Robust rank aggregation (RRA) of genes negatively selected in CRISPR-Cas9 screen of MB157R cells treated with GSI (10 μM) for 14 days. (**B**) Representative immunoblotting of N1-ICD, SOX2 and EMT/Stemness markers derived from MB157 or MB157R cells as indicated. (**C**) Cell cycle analysis of MB157R cells 72 h after transfection with siRNA SOX2 or Ctrl, $n = 5$. (**D**) Number and size of tumorspheres derived from MB157R cells with siRNA SOX2 or Ctrl, $n = 3$. (**E**) Relative mRNA expression of *NOTCH1* and its target genes (*CCND1*, *MYC*, *HES1*) and EMT/Stemness markers in iSOX2 or iEV control MB157 cells, 72 h after DOX induction, $n = 3$. (**F**) Cell growth inhibition of HCC1599 treated with GSI (1 μM) for 6 days, $n = 4$. (**G**) Cell proliferation assay of HCC1599 cells treated with GSI (1 μM) for 6 days, normalized to day 0, $n = 3$. (**H**) Relative mRNA expression of *CCND1*, *MYC* and *HES1* in HCC1599 treated with GSI (1 μM) for 24 h, $n = 3$. (**I**) Representative immunoblotting of N1-ICD and MYC derived from HCC1599 cells treated with GSI (1 μM) for 24 h, $n = 3$. (**J**) Relative mRNA expression of *NOTCH1* and its target genes (*CCND1*, *MYC*, *HES1*) and EMT/Stemness markers, $n = 3$ and (**K**) Representative immunoblotting of N1-ICD, SOX2, N-Cadherin and Claudin-3 derived from iSOX2 or iEV control HCC1599 cells, 72 h after DOX induction, $n = 3$. (**L**) Cell proliferation assay of iSOX2 or iEV control HCC1599 cells treated with GSI (1 μM) or VHC. Cell growth was assessed 6 days post treatment and normalized to day 0, $n = 4$. (**M**) Cell proliferation assay of HCC1806 cells treated with GSI (10 μM) for 6 days, normalized to day 0, $n = 3$. (**N**) Cell proliferation assay of HCC1806 cells 72 h after transfection with siRNA SOX2 or Ctrl, normalized to day 0, $n = 3$. (**O**) Representative immunoblotting of N1-ICD, SOX2 and SLUG derived from HCC1806 cells 72 h after transfection with siRNA SOX2 or Ctrl, $n = 3$. (**P**) Number and size of tumorspheres derived from HCC1806 cells with siRNA SOX2 or Ctrl, $n = 3$. Data from biological replicates are represented as mean ± SEM. Student *t* test (**C–E**, **H**, **J**, **P**) two-way ANOVA (**G**, **N**) or one-way ANOVA (**L**) were used to determine *P* value.

© The Author(s)

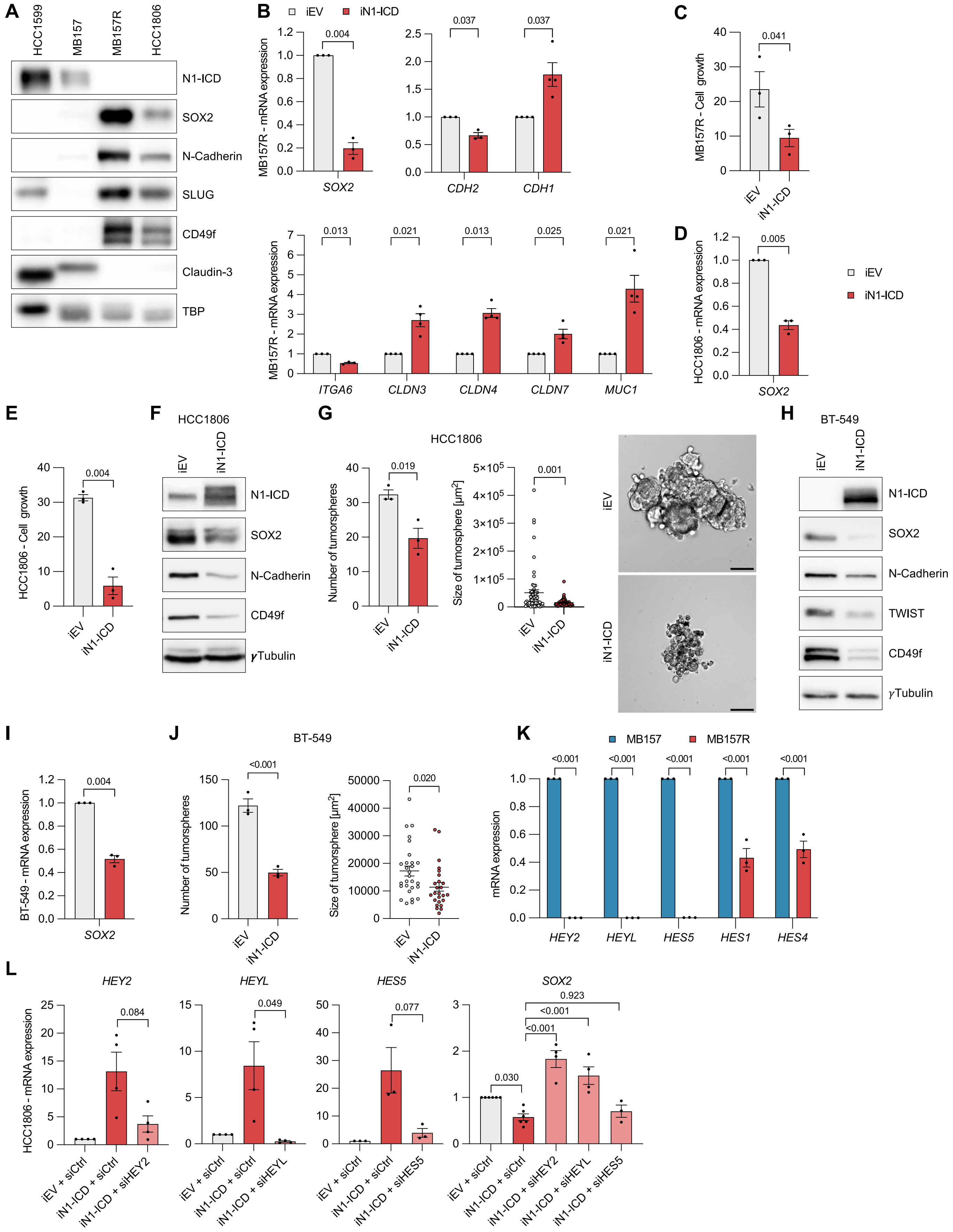
A
HCC1599
MB157
MB157R
HCC1806
N1-ICD
SOX2
N-Cadherin
SLUG
CD49f
Claudin-3
TBP
B
iEV
iN1-ICD
MB157R - mRNA expression
SOX2
CDH2
CDH1
ITGA6
CLDN3
CLDN4
CLDN7
MUC1
C
MB157R - Cell growth
D
HCC1806 - mRNA expression
E
HCC1806 - Cell growth
F
HCC1806
γTubulin
G
Number of tumorspheres
Size of tumorsphere [μm²]
H
BT-549
TWIST
I
BT-549 - mRNA expression
J
K
MB157
MB157R
mRNA expression
HEY2
HEYL
HES5
HES1
HES4
L
iEV + siCtrl
iN1-ICD + siCtrl
iN1-ICD + siHEY2
iN1-ICD + siHEYL
iN1-ICD + siHES5

◀ **Figure EV3. Reciprocal SOX2 inhibition is mediated through Notch downstream transcriptional repressors of the HEY family.**

(**A**) Representative immunoblotting of N1-ICD, SOX2 and EMT/Stemness markers derived from HCC1599, MB157, MB157R and HCC1806 cells. (**B**) Relative mRNA expression of *SOX2* and EMT/Stemness markers in iN1-ICD or iEV control MB157R cells, 72 h after DOX induction $n = 3$–4. (**C**) Cell proliferation assay of iN1-ICD or iEV control MB157R cells. Cell growth was assessed 6 days after DOX induction and normalized to day 0, $n = 4$. (**D**) Relative mRNA expression of *SOX2* in iN1-ICD or iEV control HCC1806 cells, 72 h after DOX induction, $n = 3$. (**E**) Cell proliferation assay of iN1-ICD or iEV control HCC1806 cells. Cell growth was assessed 6 days after DOX induction and normalized to day 0, $n = 3$. (**F**) Representative immunoblotting of N1-ICD, SOX2 and EMT/Stemness markers derived from iN1-ICD or iEV control HCC1806 cells, 72 h after DOX induction. (**G**) Number and size of tumorspheres with representative pictures derived from iN1-ICD compared to iEV control HCC1806 cells after 14 days, $n = 3$. Scale = 100 µm. (**H**) Representative immunoblotting of N1-ICD, SOX2 and EMT/Stemness markers and (**I**) Relative mRNA expression of *SOX2* in iN1-ICD or iEV control BT-549 cells, 72 h after DOX induction $n = 3$. (**J**) Number and size of tumorspheres derived from iN1-ICD compared to iEV control BT-549 cells after 14 days, $n = 3$. (**K**) Relative mRNA expression of *HEY/HES* family genes in MB157 and MB157R cells, $n = 3$. (**L**) Relative mRNA expression of *HEY2*, *HEYL*, *HES5* and *SOX2* in iN1-ICD or iEV control HCC1806 cells 72 h after transfection with siRNA HEY2, HEYL, HES5 or Ctrl $n = 3$–4. Data from biological replicates are represented as mean ± SEM. Student *t* test (**B–E**, **G**, **I–L**) or one-way ANOVA (**L**) were used to determine *P* value.

 © The Author(s)

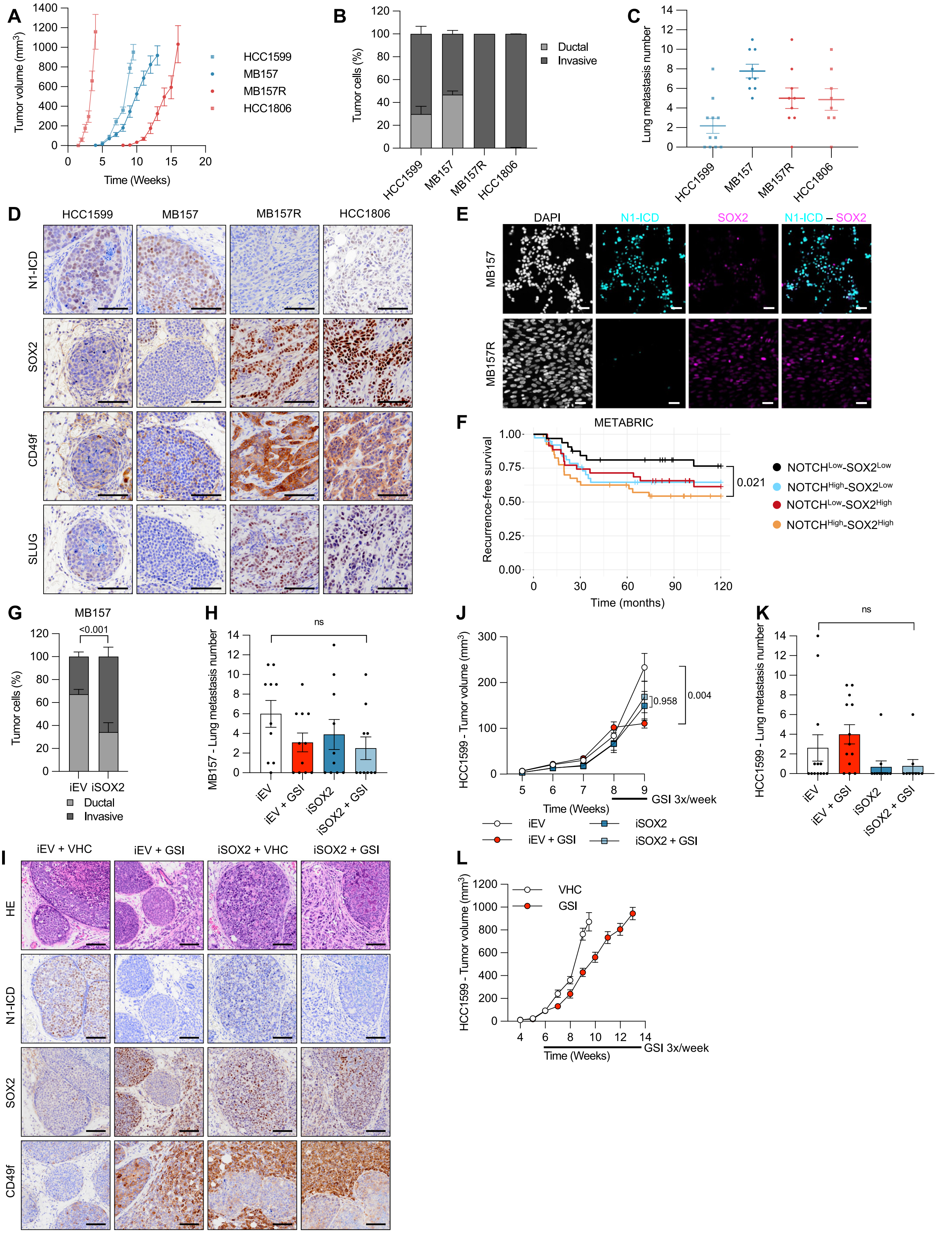
A
Tumor volume (mm3)
Time (Weeks)
HCC1599
MB157
MB157R
HCC1806
B
Tumor cells (%)
Ductal
Invasive
C
Lung metastasis number
D
HCC1599
MB157
MB157R
HCC1806
N1-ICD
SOX2
CD49f
SLUG
E
DAPI
N1-ICD
SOX2
N1-ICD – SOX2
MB157
MB157R
F
METABRIC
Recurrence-free survival
Time (months)
0.021
NOTCHLow-SOX2Low
NOTCHHigh-SOX2Low
NOTCHLow-SOX2High
NOTCHHigh-SOX2High
G
MB157
<0.001
Tumor cells (%)
iEV iSOX2
Ductal
Invasive
H
MB157 - Lung metastasis number
ns
iEV
iEV + GSI
iSOX2
iSOX2 + GSI
J
HCC1599 - Tumor volume (mm3)
0.958
0.004
Time (Weeks)
GSI 3x/week
iEV
iEV + GSI
iSOX2
iSOX2 + GSI
K
HCC1599 - Lung metastasis number
ns
I
iEV + VHC
iEV + GSI
iSOX2 + VHC
iSOX2 + GSI
HE
N1-ICD
SOX2
CD49f
L
HCC1599 - Tumor volume (mm3)
VHC
GSI
Time (Weeks)
GSI 3x/week

© The Author(s)

Figure EV4. Escape of GSI-mediated in vivo tumor growth control due to TNBC tumor cell plasticity.

(**A**) Tumor growth of HCC1599, MB157, MB157R and HCC1806 MIND xenografts ($n = 9$–11). (**B**) Quantification of tumor cells in invasive or ductal areas from HE coloration in HCC1599, MB157, MB157R and HCC1806 MIND xenografts at endpoint (~ 1000 mm^3) ($n = 3$). (**C**) Lung metastasis number in HCC1599, MB157, MB157R and HCC1806 MIND xenografts at endpoint, $n = 9$–11. (**D**) Representative pictures of N1-ICD, SOX2, CD49f and SLUG IHC stainings for HCC1599, MB157, MB157R and HCC1806 MIND xenograft tumors at endpoint, scale = 100 µm. (**E**) Representative images of co-immunofluorescence staining of N1-ICD – SOX2 for MB157 and MB157R cell lines in vitro, scale = 50 µm. (**F**) RFS of TNBC patients from METABRIC dataset with $NOTCH^{High/Low}$ signature and $SOX2^{High/Low}$ expression. TNBC patients are divided in 4 groups: $NOTCH^{High}/SOX2^{High}$ ($n = 40$), $NOTCH^{High}/SOX2^{Low}$ ($n = 38$), $NOTCH^{Low}/SOX2^{High}$ ($n = 35$) and $NOTCH^{Low}/SOX2^{Low}$ ($n = 32$). (**G**) Quantification of tumor cells in invasive or ductal areas from HE coloration in MB157-iSOX2 xenografts, $n = 3$. (**H**) Lung metastasis number in SOX2-expressing or EV control MB157 MIND xenografts treated with GSI (8 mg/kg, 3×/week) or VHC for 1 week, $n = 10$–11. (**I**) Representative pictures of HE coloration, N1-ICD, SOX2 and CD49f IHC stainings for iSOX2 or iEV control MB157 MIND xenografts treated with GSI (8 mg/kg, 3x/week) or VHC for 1 week, scale = 100 µm. (**J**) Tumor growth of iSOX2 or iEV control HCC1599 MIND xenografts treated with GSI (8 mg/kg, 3×/week) or VHC for 1 week, $n = 9$–13. (**K**) Lung metastasis number in iSOX2 or iEV control HCC1599 MIND xenografts treated with GSI (8 mg/kg, 3×/week) or VHC for 1 week, $n = 9$–13. (**L**) Tumor growth of HCC1599 xenografts treated with GSI (8 mg/kg) or VHC for 8 weeks, $n = 9$–13. Data from biological replicates are represented as mean ± SEM. Log-rank test (**F**), Cochran–Mantel–Haenszel test (**G**), one-way ANOVA (**H**, **K**) or two-way ANOVA (**J**), were used to determine *P* value.

 © The Author(s)

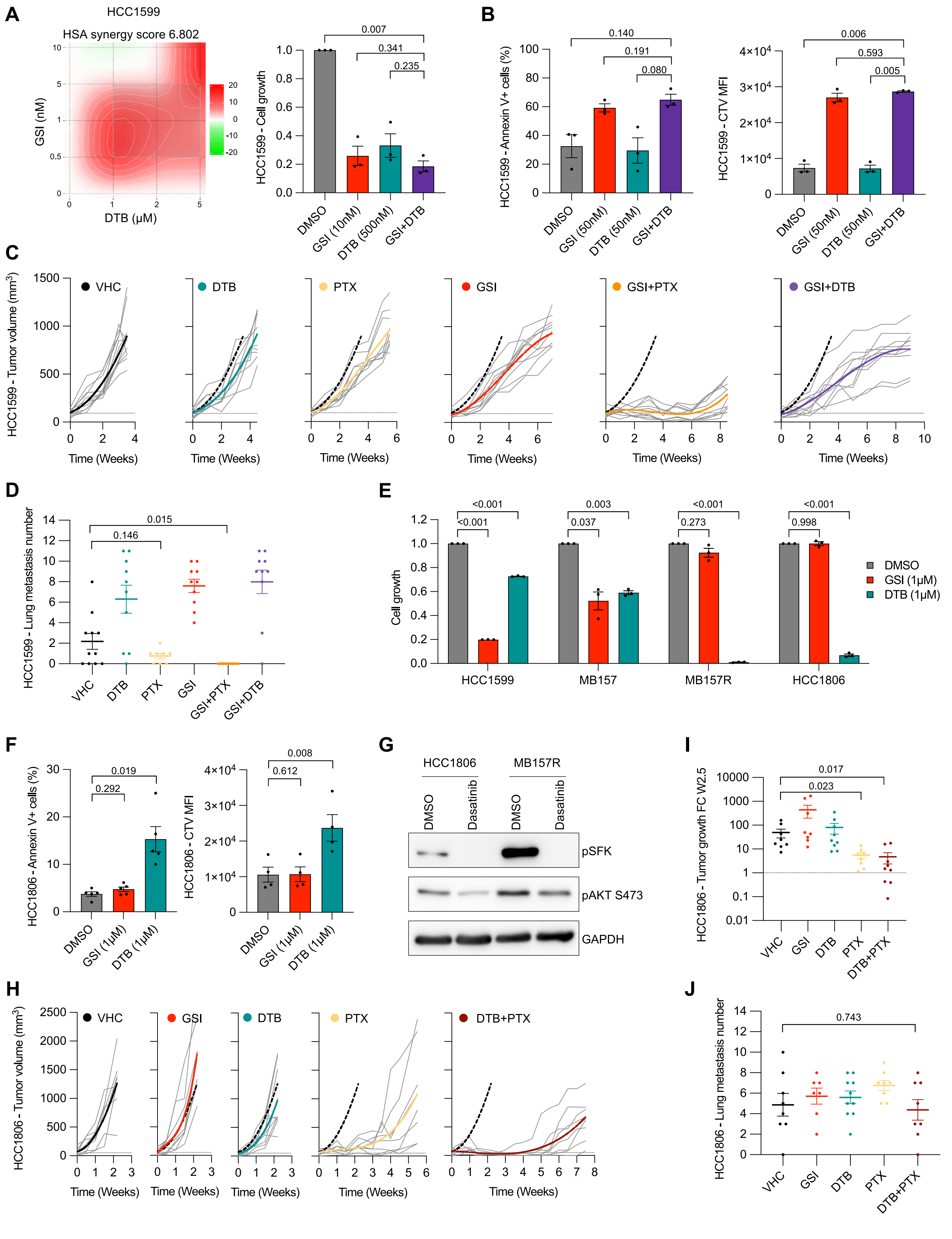
A
HCC1599
HSA synergy score 6.802
GSI (nM)
DTB (μM)
HCC1599 - Cell growth
DMSO
GSI (10nM)
DTB (500nM)
GSI+DTB
0.007
0.341
0.235
B
HCC1599 - Annexin V+ cells (%)
DMSO
GSI (50nM)
DTB (50nM)
GSI+DTB
0.140
0.191
0.080
HCC1599 - CTV MFI
0.006
0.593
0.005
C
HCC1599 - Tumor volume (mm³)
VHC
DTB
PTX
GSI
GSI+PTX
GSI+DTB
Time (Weeks)
D
HCC1599 - Lung metastasis number
0.015
0.146
VHC
DTB
PTX
GSI
GSI+PTX
GSI+DTB
E
Cell growth
<0.001
<0.001
0.003
0.037
<0.001
0.273
<0.001
0.998
HCC1599
MB157
MB157R
HCC1806
DMSO
GSI (1μM)
DTB (1μM)
F
HCC1806 - Annexin V+ cells (%)
0.019
0.292
DMSO
GSI (1μM)
DTB (1μM)
HCC1806 - CTV MFI
0.008
0.612
G
HCC1806
MB157R
DMSO
Dasatinib
DMSO
Dasatinib
pSFK
pAKT S473
GAPDH
I
HCC1806 - Tumor growth FC W2.5
0.017
0.023
VHC
GSI
DTB
PTX
DTB+PTX
H
HCC1806 - Tumor volume (mm³)
VHC
GSI
DTB
PTX
DTB+PTX
Time (Weeks)
J
HCC1806 - Lung metastasis number
0.743
VHC
GSI
DTB
PTX
DTB+PTX

Figure EV5. Combination therapies to treat GSI-sensitive and -resistant TNBC xenografts.

(**A**) HAS-synergy heatmap derived from HCC1599 cells treated with a concentration matrix of GSI-DTB for 6 days and cell growth inhibition of HCC1599 cells treated with GSI (10 nM) and/or DTB (500 nM) for 6 days, $n = 3$. (**B**) Proportion of Annexin V+ cells ($n = 4$) and Cell Trace Violet mean fluorescence intensity (MFI) ($n = 3$) in HCC1599 cells treated with GSI (50 nM) and/or DTB (50 nM) for 6 days, by flow cytometry analysis. (**C**) Tumor growth of HCC1599 xenografts treated with GSI (8 mg/kg, 3×/week), DTB (15 mg/kg, 5×/week), PTX (15 mg/kg, 1×/week) or VHC, $n = 9$–11. (**D**) Lung metastasis number in HCC1599 xenografts treated with GSI (8 mg/kg, 3x/week), DTB (15 mg/kg, 5x/week), PTX (15 mg/kg, 1×/week) or VHC, $n = 8$-11. (**E**) Cell growth inhibition of HCC1599, MB157, MB157R and HCC1806 cells treated with GSI and/or DTB for 6 days, $n = 3$. (**F**) Proportion of Annexin V+ cells ($n = 5$) and Cell Trace Violet MFI (n = 4) in HCC1806 cells treated with GSI (1 μM) or DTB (1 μM) for 3 days, by flow cytometry analysis. (**G**) Representative immunoblotting of pSFK and pAKT S473 derived from MB157R and HCC1806 cells treated with Dasatinib 1 μM for 18 h (**H**) Tumor growth of HCC1806 MIND xenografts treated with GSI (8 mg/kg, 3x/week), DTB (15 mg/kg, 5×/week), PTX (15 mg/kg, 1×/week) or VHC, $n = 8$–10. (**I**) Tumor growth fold change (2.5 weeks after treatment) of HCC1806 MIND xenografts treated with GSI (8 mg/kg, 3×/week), DTB (15 mg/kg, 5x/week), PTX (15 mg/kg, 1×/week) or VHC, $n = 6$–9. (**J**) Lung metastasis number in HCC1806 MIND xenografts treated with GSI (8 mg/kg, 3×/week), DTB (15 mg/kg, 5×/week), PTX (15 mg/kg, 1×/week) or VHC, $n = 8$–10. Data from biological replicates are represented as mean ± SEM. One-way ANOVA (**A**, **B**, **D**–**F**, **I**, **J**) was used to determine *P* value.

 © The Author(s)
